# How Many Genes Are Expressed in a Transcriptome? Estimation and Results for RNA-Seq

**DOI:** 10.1371/journal.pone.0130262

**Published:** 2015-06-24

**Authors:** Luis Fernando García-Ortega, Octavio Martínez

**Affiliations:** Laboratorio Nacional de Genómica para la Biodiversidad (Langebio), Centro de Investigación y de Estudios Avanzados del Instituto Politécnico Nacional (Cinvestav-IPN), Irapuato, Guanajuato, México; Swiss Federal Institute of Technology (ETH Zurich), SWITZERLAND

## Abstract

RNA-seq experiments estimate the number of genes expressed in a transcriptome as well as their relative frequencies. However, an undetermined number of genes can remain undetected due to their low expression relative to the sample size (sequence depth). Estimation of the true number of genes expressed in a transcriptome is essential in order to determine which genes are exclusively expressed in specific tissues or under particular conditions. A reliable estimate of the true number of expressed genes is also required to accurately measure transcriptome changes and to predict the sequencing depth needed to increase the proportion of detected genes. This problem is analogous to ecological sampling problems such as estimating the number of species at a given site. Here we present a non-parametric estimator for the number of undetected genes as well as for the extra sample size needed to detect a given proportion of the undetected genes. Our estimators are superior to ones already published by having smaller standard errors and biases. We applied our method to a set of 32 publicly available RNA-seq experiments, including the evaluation of 311 individually sequenced libraries. We found that in the majority of the cases more than one thousand genes are undetected, and that on average approximately 6% of the expressed genes per accession remain undetected. This figure increases to approximately 10% if individual sequencing libraries are analyzed. Our method is also applicable to metagenomic experiments. Using our method, the number of undetected genes as well as the sample size needed to detect them can be calculated, leading to more accurate and complete gene expression studies.

## Introduction

### The transcriptome

The transcriptome can be considered as the set of all RNA molecules, including mRNAs, rRNAs, tRNAs, and other non-coding RNAs such as small RNAs, present in a cell under specific conditions (see for example [1]). In the present work, we specifically refer to the mRNA transcriptome, but the ideas and methods discussed are applicable to other types of RNA or, in fact, to any situation where a similar sampling scheme is employed. RNA-seq [2] is a method to explore and quantitate the transcriptome, usually by high-throughput sequencing. In these experiments, total RNA is isolated from a cell population and then the mRNA fraction is converted to cDNA which is fragmented and sequenced massively in parallel, obtaining a large number of small gene tags, that are associated with specific gene transcripts. These sequences are then mapped to a reference (generally a reference genome or a *de novo* assembled reference transcriptome), obtaining quantitative data concerning transcript abundance for genes in the reference. The final result of an RNA-seq experiment performed over a given sample of mRNA is a vector, such that **y** = (*y*_1_, *y*_2_, ⋯, *y*_*g*_), where each *y*_*i*_ > 0; *i* = 1, 2, ⋯, *g* is the count of the number of tags found for the *i* − *th* gene and *g* is the total number of genes detected or estimated in the sample of effective size *N* = ∑*y*_*i*_. It is important to underline that the number of genes detected in a given RNA-seq experiment, *g*, is only an estimate of the true number of genes that are being expressed in that case, say *G*; *G* ≥ *g*. Thus, after performing an RNA-seq experiment we can only affirm that there are at least *g* genes expressed for that case, but we cannot rule out the possibility that more genes are being expressed, but were missed by our sample of size *N*, i.e., the case when *G* > *g*. An important goal of transcriptomics research is to obtain complete transcriptome-level information for each cell type that comprises the organism being studied. However, what is generally feasible is to extract mRNA from a large number of cells. Under these conditions sampling is conducted with replacement from a conceptually infinite population of molecules. In other words, the probability of mapping a tag to a specific gene does not alter the probabilities of mapping tags for each gene as the sampling proceeds. Although single-cell RNA-seq is becoming feasible [3], the majority of RNA-seq experiments performed to date use RNA extracted from heterogeneous mixtures of cells such as tissues, organs or even complete individuals. This increases the complexity of the population sampled by increasing the number of distinct transcripts present, for example derived from the set of genes whose expression is restricted to only a particular cell type(s).

When a gene is not detected by RNA-seq in a particular treatment, it can be due to the fact that it is not expressed or, alternatively, it was expressed but was not detected because the sample size was too small. In the former case no error is committed, but the later leads to an incorrect conclusion, if the lack of detection is taken as absolute evidence of no expression. RNA-seq literature is full of cases where the authors claim that some genes are ‘exclusively expressed’ in a particular condition. For example, in some cancer related studies [4–7] the authors affirm that a set of genes are exclusively expressed in the malignant tissues, while in many more cases the same claim is done about exclusive expression at some treatment or condition. We estimated, by searching the literature, that in around 600 papers such claims are made; see [8–18] for particular examples. To claim that a particular gene is exclusively expressed under a given condition, the researchers must show that the collection of expressed genes is reasonable complete and thus there is unlikely that the undetected gene was missed as a consequence of a small sample size.

### Sampling genes is analogous to sampling species

The problem studied here, namely the estimation of undetected genes in a transcriptome, is analogous to the problem of estimating the number of species (in ecology) or classes (in statistics) [19]. Concrete examples include the ecological question of how many species exist in a delimited area, or to the estimation of the number of words known by a writer [20]. This is a difficult problem because it involves an estimate of how many things (classes, species, genes) are missing in a sample, using only the information contained in the sample itself.

To present the problem, as well as putative solutions in a formal framework, denote as *f*_*r*_ the number of cases in which the counts of the class *i*, i.e., the *y*_*i*_’s have exactly the value of *r*; *r* = 1, 2, ⋯. Thus, for example, if 5 of the *y*_*i*_’s are equal to 1, then *f*_1_ = 5. In this way, and without losing any relevant information, the original data **y** can be represented by the vector **f** = (*f*_1_, *f*_2_, ⋯) and the sample size, *N*, can be expressed as

$$\begin{array}{c} \sum_{r=1}^{r=\infty }rf_{r}=N \end{array}$$

This notation was apparently proposed in [21], in which it was used to estimate the number of classes in a population of known size. This notation was followed by [22], in which *f*_*r*_ was defined as the frequency of the frequency *r*. Note that ∑_*i*_ *y*_*i*_ = ∑_*r*_ *rf*_*r*_ = *N* and in any particular case the maximum value of *r* is finite, however we use ‘*r* = ∞’ above simply to indicate that the sum must be performed for all values of *r*. ‘*f*_0_’ can then be used to represent the number of genes that are not present in a sample, or in more general terms, the number of classes that are missing in a particular sample (see Section A of S1 File for more details).

Consider a sampling experiment in which a biologist is interested in knowing how many species of fish live in a pond. After catching a fish and noting its species, say *i*, the frequency corresponding to that species, *y*_*i*_, increases by one. The fish is sent back to the pond (sampling with replacement considering an ‘infinite’ population) and the procedure is repeated. In this scenario important variables change as *N*, the number of times that the procedure is repeated or sample size, increases. In the first stages the rate of discovery of species is large and *g* (the number of detected species) increases rapidly; at the same time the number of species represented by a single individual, the frequency *f*_1_, is large: *f*_1_ ≈ *N*. As the process continues, the discovery of new species becomes less and less frequent such that *g* tends to stability converging on the true number of species in the pond, *G*. Precisely the same logic applies to the analysis of transcriptome data in an RNA-seq experiment, where the probability of mapping to a previously unsampled gene is large when *N* is small. As *N* increases, the probability of mapping a sequence to a previously unsampled gene decreases. This process can be plotted as a rarefaction curve, and used to estimate *G* (see for example [23]). Intuitively, a ‘stopping rule’ can be established for the sampling procedure; for example, “stop sampling when *f*_1_ = 0”, when all of the *y*_*i*_’s are larger than one. This is a reasonable rule of thumb because when *f*_1_ = 0 it is assumed that the sample has covered the complete population, including all or nearly all of the species present. For increased confidence, “stop sampling when *f*_1_ = 0 and *f*_2_ = 0”, etc. In summary, the values of *f*_*r*_ when *r* is small, say *r* = 1, 2, ⋯, 6 contain most of the information about the ‘completeness’ of the sample. In the case of transcriptome data, note that the total number of genes, *G*, is equal to the number of genes detected in the sample, *g*, plus the number of genes missing, *f*_0_,

$$\begin{array}{c} G=g+f_{0} \end{array}$$

### The need for an estimate of the number of missing genes (*f*_0_)

To model the frequencies of expression of genes, the number of classes must first be fixed to a given value *G* = *c*. After doing this, a multinomial distribution with *c* parameters, or a negative binomial or a set of *c* independent Poisson distributions, etc. can be assumed. In fact, all current RNA-seq analysis algorithms such as edgeR [24] and DEGseq [25] assume that the number of genes expressed in a sample is equal to the number of genes found in the sample, *G* = *g*, and only then model the frequencies under a specific distribution. This is a rational assumption, given the impossibility of estimating frequencies of expression for genes that are not detected in the sample. However, there are important statistical and biological repercussions to this problem that have been under-appreciated in the literature (see for example [26]). From a statistical point of view, when the true value of G is unknown, the parameter space is open. In other words, we do not know how many parameters need to be estimated, and thus the method of maximum likelihood fails to give proper estimators [22]. On the practical side, if information concerning the completeness or richness of a sample is unknown, then it is impossible to evaluate the possibility that a gene was indeed expressed but was missed during sampling. This implies that for several classes of genes, particularly those that are only weakly expressed, it is impossible to determine whether their expression is restricted to a particular cell type, developmental stage, or environmental condition. This is a crucial consideration given that genes with important regulatory roles, as for example those encoding transcription factors, are usually expressed at lower frequencies [27] and thus have larger probabilities to remain undetected in the sample than are other types of genes.

The estimation (using DNA evidence) of the number of microorganism species in metagenomics experiments is a problem with the identical sampling and statistical framework as the one presented here for the estimation of undetected genes, and is amply represented in the literature [28–35]. A solution to the problem of *f*_0_ estimation in RNA-seq is likely to be directly applicable to metagenomic experiments.

### Non-parametric estimators for the number of missing genes (*f*_0_)

The estimation of the number of missing or undetected classes, *f*_0_, can be performed by different methods, depending on the structure of the population and the sampling scheme employed [19]. RNA-seq employs sampling with replacement, thus assuming a population of infinite size in which *G* is unknown. In this case, selecting a discrete distribution, such as multinomial or negative binomial, is impossible without conditioning to a known value of *G*, for example *G* = *g*. However, it is important to note that *G* itself is a random variable in that the realized value *G* = *g* holds only after the sample had been obtained; assuming a priori a particular distribution for *G*, as for example log-normal [23], is risky and without empirical foundations given that RNA-seq samples arise from a wide range of situations (heterogeneous mixtures of distinct cell types as tissues or organs in distinct environmental conditions or developmental stages, etc. [26]). For this wide range of possibilities it appears unrealistic to impose a given statistical law in the form of a distribution for the number of classes. At least for RNA-seq, it appears safer to use non-parametrical estimation procedures, assuming very little about the distribution, as has been done for example in [22]. Here we present only the most common non-parametric estimators for *f*_0_ proposed in the literature. A more comprehensive list, including different methods of estimation can be found in [19].

In a seminal work, I. J. Good [22] studied the problem of the estimation of the relative frequency of occurrence of species, which as previously described, is directly applicable to the estimation of the relative frequency of detection of genes in a transcriptome. He showed that the usual relative frequency in a sample, *y*_*i*_/*N* or *r*/*N*, is a sensible estimator of the corresponding relative frequencies only when the true number of classes *G* is known. In that case those are Maximum Likelihood Estimators (MLE) of the corresponding parameters. However, when *G* is unknown, which is the case in all RNA-seq experiments, these estimators are inappropriate for small *r*, i.e., for genes only weakly expressed. In [22] Good presented an approximate recurrent expression for the expected value of *r*,

$$\begin{array}{c} r^{*}\approx \left(r+1\right)f_{r+1}/f_{r} \end{array}$$ (1)

This relation was first discovered by Alan M. Turing [36], and has been the basis of estimators for the coverage of a sample and, in particular, for estimators of the number of classes under different frameworks. Anne Chao in [37] used an approximate and asymptotic result to propose as estimator of *f*_0_, the function

$$\begin{array}{c} {\hat{f}}_{0}=\frac{f_{1}^{2}}{2f_{2}} \end{array}$$ (2)

This estimator is generally called ‘*Chao1*’ in the literature. This estimator set the foundation for variants to estimate *f*_0_ under distinct sampling schemes in the framework of species richness estimation, as for example the ‘*Chao2*’ estimator,

$$\begin{array}{c} {\hat{f}}_{0}=\frac{f_{1}\left(f_{1}-1\right)}{2(f_{2}+1)} \end{array}$$

which is bias corrected and is always obtainable [38]. Chao1 is a lower bound and thus a biased estimator of *f*_0_, a fact already noted in [37]. However, resampling procedures, such as Jackknife or bootstrap [39] can be employed to reduce the bias and obtain non-parametric confidence intervals, as proposed in [37, 38]. The Chao1 and Chao2 estimators have been used to derive nonparametric lower bounds for the number of species shared by multiple communities [40], and evaluated taking into account the influence of rare species [41] as well as in comparisons of these estimators’ performance [42, 43].

Recently the group of Anne Chao developed an improved estimator for the number of missing classes in the framework of species estimation [44]. This estimator, also based in the Good–Turing recurrent expression (Eq 1), is called ‘*iChao1*’ and given by

$$\begin{array}{c} {\hat{f}}_{0}=\frac{f_{1}^{2}}{2f_{2}}+[(\frac{f_{3}}{4f_{4}})\times \max(f_{1}-(\frac{f_{2}f_{3}}{2f_{4}}),0)] \end{array}$$ (3)

The problem of the estimation of the number of expressed genes has been previously studied for the sampling of ‘Expressed Sequence Tags’ or ‘EST’ libraries [45]. Experiments quantifying the relative expression of genes based on the frequencies of their corresponding ESTs are similar in this respect to RNA-seq experiments but differ in that EST experiments usually involve much smaller sample sizes (many fewer ESTs in a sample library) and longer gene tags. The estimation of species richness in this framework has been treated in [46–48]. The probability of discovering a new class (gene) in this framework is presented in [46]. In [47] the concept of gene capture prediction and overlap estimation is expanded from one to multiple libraries and [48] gives a penalized non-parametric maximum likelihood estimator for species richness while [49] discusses which sequencing depth might be sufficient to interrogate gene expression profiling in chicken libraries by RNA-Seq.

In the context of uniquely expressed genes (or mRNAs) in specific cells and tissues, [50] presents an estimator for *f*_0_. This estimator, named the ‘Medial’ estimator is given by

$$\begin{array}{c} {\hat{f}}_{0}=(\frac{N-1}{N})(\frac{f_{1}\left(f_{1}-1\right)}{f_{2}+1})\approx \frac{f_{1}\left(f_{1}-1\right)}{f_{2}+1} \end{array}$$ (4)

Note that the factor (*N* − 1)/*N*, is not relevant in the context of RNA-seq where the sample sizes, *N*, are in the order of millions. Asymptotic expression for the variances of the Chao1 (Eq 2) and Medial (Eq 4) estimators are presented in [51] and [50], respectively. However, we consider that the bootstrap approach [39] gives a more robust approach for the estimation of the variance of these estimators than the asymptotic approximations. The uncorrected forms of the Chao1, iChao1 and Medial estimators (Eqs 2, 3 and 4) do not have a finite expectation. This is due to the fact that the denominators of the equations can take a value of zero with non-zero probabilities, and thus the sum that defines the corresponding expectations diverges.

Here we propose and evaluate a set of new non-parametric estimators for *f*_0_. We present an estimator of *f*_0_ that is superior to the Chao1, iChao1 and Medial estimators in the framework of RNA-seq. We use the selected function to estimate the number of missing genes in a set of RNA-seq experiments, demonstrating that, in many cases, a substantial number of genes is not represented in these studies. We also propose and test estimators for the extra sample size needed to complete the estimated gene set to include an arbitrary large proportion of the genes expressed.

## Results and Discussion

A possible answer to the question that titles this paper ‘How many genes are expressed in a transcriptome?’ is simply the number of genes detected in the sample(s). This naive answer generates an estimator (the *naive* estimator ${\hat{f}}_{0}\equiv 0$ for any sample) that will almost always underestimate the true value of the parameter, because the probability of missing one or more genes can be very large, approaching 100% in almost all real cases (see Section B of S1 File). We sought to identify more robust estimators for *f*_0_ than the Chao and Medial estimators, at least for the framework of RNA-seq studies.

### Better estimators of *f*_0_ for RNA-seq

As noted among others by Good [22] and Chao [37, 38], the frequencies of rare classes (*f*_1_, *f*_2_, ⋯, *f*_*r*_ with *r* *small*) carry most of the information about the number of missing classes, *f*_0_. This leads to the Chao1 estimator (Eq 2), which uses only the singletons (*f*_1_) and doubletons (*f*_2_) to estimate the number of missing classes [37], and very recently to the iChao1 estimator (Eq 3), which apart from *f*_1_ and *f*_2_ uses the information from *f*_3_ and *f*_4_[44]. In the ecological framework of species estimation, there is no point in exploring estimators that use the information of frequencies of frequencies with larger order, say, *f*_*r*_ with *r* > 4, because in that context the sample sizes are limited to relatively small values, say *N* ≤ 1000, and thus the observed values of *f*_*r*_, *r* > 4 are very frequently equal to zero. In contrast, in RNA-seq experiments the sample sizes are much larger; from hundreds of thousands to tens of millions of mapped gene tags. As a consequence, in RNA-seq datasets the observed values of *f*_*r*_, *r* = 4, 5, ⋯, 10 are, in most of the cases, larger than zero and thus can be used for the estimation of *f*_0_. We heuristically explored the use of functions that employ, apart from the observed values of *f*_1_ and *f*_2_, the values of *f*_3_, *f*_4_, ⋯, *f*_10_. We reasoned that these small frequencies carry information about *f*_0_. In particular we explored, among others, functions of the form

$$\begin{array}{c} {\hat{f}}_{0}=u\;\frac{f_{1}^{2}}{c(f_{2},f_{3},\cdots ,f_{10})} \end{array}$$ (5)

where the constant *u* is an scalar to be determined and the function *c*() is a measure of central tendency for *f*_2_, *f*_3_, ⋯, *f*_10_ or a subset of these quantities. As putative functions of central tendency, *c*(), we used the Pythagorean means, i.e., the arithmetic mean or average as well as the geometric and harmonic means.

To evaluate putative estimators of *f*_0_ we required to have an RNA-seq dataset that could be considered ‘complete’ in the sense that every gene expressed was detected by one or more tags, i.e., a sample with not missing genes. As evidence that a dataset could be considered complete, we employed a rule that all genes must be represented by at least two tags, such that *f*_1_ = 0. This criterion has been proposed as a ‘stopping rule’ for sampling in various studies, for example in [52]. Note that in such cases the Chao1, Medial and iChao1 estimators (Eqs 2, 3 and 4, respectively), as well as any estimator defined by (5) return values of zero as estimates of the number of missing genes.

Accepting a given sample as complete is equivalent to assuming that the true value for the number of expressed genes is equal to the number of genes observed in that sample, say, *G* = *g*, and this implies that *f*_0_ = 0 because *G* = *g* + *f*_0_. A complete sample can be used to take sub-samples of smaller size in which we know the true value of the number of missing genes, *f*_0_, and this implies that we can test different estimators of the parameter and study their statistical properties by repeating the process of sub-sampling.

Many RNA-seq datasets are deposited in the GEO [53] and ArrayExpress [54] public databases of gene expression profiles. We explored these datasets by downloading the auxiliary files that include the counts for each sequenced library in the accession, i.e., the vectors of gene tag counts **y**. The accession with identifier GSE1581, corresponding to the ‘MPSS mouse transcriptome analysis project’ has been used in several studies (see [55–59]). For our purposes, this dataset fulfilled the criterion *f*_1_ = 0 when adding 35 libraries from different organs, and thus was considered a complete sampling of the mouse transcriptome (see Analysis). This accession comprises data for a total of *g* = 23332 expressed genes with a total sample size of *N* = 160552086 mapped gene tags.

### Selection of an *f*_0_ estimator

Having a complete sample, we evaluated distinct estimators of *f*_0_ by resampling the original distribution *via* the bootstrap procedure and measuring the standard error of each estimator in each pseudo replicate. The formula for the estimated standard error is given by

$$\begin{array}{c} se\left({\hat{f}}_{0}\right)=\sqrt{\frac{1}{B}\sum_{i=1}^{i=B}{\left({\hat{f}}_{0i}-f_{0i}\right)}^{2}} \end{array}$$ (6)

where *B* is the number of pseudo-replicates and ${\hat{f}}_{0i},f_{0i}$ are the estimated and true values of *f*_0_ in the *i* − *th*, replicate, *i* = 1, 2, ⋯, *B*. We considered the best estimator to be the one with the smallest standard error over a large number of pseudo replicates obtained, assuming a wide range of sample sizes. This procedure mimics what happens in reality when sampling the transcriptome, due to the fact that a complete sample allows for the probabilities of expression to be properly estimated by maximum likelihood. To obtain pairs $\left\{{\hat{f}}_{0i},f_{0i}\right\}$ we used the parametric bootstrap procedure under the multinomial (equivalent to non-parametric bootstrap) or Poisson distributions. We assumed a random sample size, *N*_*i*_, uniformly distributed in the interval [*m* **N**, **N**], where **N** was the sample size in the complete sample, i.e., **N** = 160552086 and the constant *m* was set to *m* = 1/160.552086 ≈ 0.006228508, in such a way that the minimum sample size tested was *m* **N** ≈ 1*e*6, or one million. This minimum sample size was decided after pilot tests indicated that the behavior of the estimators was erratic for smaller samples. A large number, *B* = 100000, bootstrap samples was used to test all putative estimators, including varying the functions *c*() and empirically estimating the best value of the constant *u*. The statistical behavior of the error, ${\hat{f}}_{0}-f_{0}$, as well as correlations between the sample size, estimated values, errors etc. for all estimators were tested (see details in Section C of S1 File).

The best estimator of *f*_0_, obtained by the procedure outlined above, and presented in detail in Section C of S1 File, was

$$\begin{array}{c} h_{6}=\frac{6}{10}\;\frac{f_{1}^{2}}{H(f_{2},f_{3},\cdots ,f_{6})} \end{array}$$ (7)

where the function *H*(*f*_2_, *f*_3_, ⋯, *f*_6_) is the harmonic mean of *f*_2_ up to *f*_6_, i.e.,

$$\begin{array}{c} H\left(f_{2},f_{3},\cdots ,f_{6}\right)=\frac{5}{\sum_{r=2}^{r=6}\left(1/f_{r}\right)} \end{array}$$

thus we call this estimator *h*_6_ or *harmonic estimator of degree 6 of *f*_0_*.

As for the the Chao1, Medial and iChao1 estimators, the expectation of *h*_6_ do not exist, because the harmonic mean, *H*(*f*_2_, *f*_3_, ⋯, *f*_6_), can take a value of zero with non-zero probability and a value of zero in the denominator leads to indeterminacy. However, for large sample sizes, the probability *P*[*H*(*f*_2_, *f*_3_, ⋯, *f*_6_) = 0] is negligible, and thus we can approximate the expectation by the mean of a large number of bootstrap replicates.

Table 1 presents a numerical comparison of the Chao1, iChao1, Medial and *h*_6_ estimators of *f*_0_ (Eqs 2, 3, 4 and 7, respectively), evaluated in *B* = 100000 bootstrap replicates of the complete dataset (GSE1581). Details of the comparisons evaluated in an independent set of replicates can be consulted in Section C of S1 File.

**Table 1:** Comparison of estimators.

Estimator of *f*_0_ (${\hat{f}}_{0}$)	Standard Error		*r* ^2^	Error $\left({\hat{f}}_{0}-f_{0}\right)$			
	$se({\hat{f}}_{0})$	%*se*(*Ch*1)	(${\hat{f}}_{0},f_{0}$)	Min.	Median	Mean	Max.
Chao1	384	100.00	0.9664	-3723	-22	-140	57
iChao1	306	79.57	0.9651	-3268	-7	-91	111
Medial	141	36.59	0.9665	-1857	54	61	551
*h*_6_	85	22.04	0.9897	-1563	3	-3	438

Comparison of Chao1, iChao1, Medial and *h*_6_ estimators of *f*_0_ evaluated in *B* = 100000 bootstrap replicates of the complete dataset (accession GSE1581) using random sample sizes uniformly distributed between 1 and 160.5 million tags. Estimated standard error, $se({\hat{f}}_{0})$, percentage of standard error compared with the standard error of Chao1, %*se*(*Ch*1), estimated coefficient of determination between ${\hat{f}}_{0}$ and *f*_0_ (*r* ^2^), and statistics for the errors ${\hat{f}}_{0}-f_{0}$ (minimum, median, mean and maximum) are presented for each one of the four estimators.

From Table 1 we can see that *h*_6_ exhibits better behavior than the other three estimators in an ample interval of sample sizes, going from 1 to 160.5 million tags (this last figure is the sample size of the complete RNA-seq dataset). The *h*_6_ estimator is superior to Chao1, iChao1 and Medial in having an estimated standard error much smaller than either of the three, a raw value of 85 representing only 22%, 28% and 60% of the standard errors of the Chao1, iChao1 and Medial estimators, respectively. The value of Pearson’s determination coefficient between ${\hat{f}}_{0}$ and *f*_0_, *r* ^2^, is ≈ 0.99 for *h*_6_, while it is smaller, ≈ 0.97, for the Chao1 and Medial estimators. This means that, on average, *h*_6_ explains a larger proportion of the variance of ${\hat{f}}_{0}$ as a linear function of *f*_0_ than either the Chao1, iChao1 or Medial estimators. Importantly, the statistics for the estimated errors of the estimators, $\left({\hat{f}}_{0}-f_{0}\right)$, are better centered around zero for *h*_6_ than for Chao1, iChao1 or the Medial, having values of 3 and -3 for the median and mean in the case of *h*_6_ and values much farther from zero for Chao1, iChao1 and the Medial estimators. The minimum and maximum of the estimated errors are also both smaller for the *h*_6_ than for Chao1 and Medial estimators.

**Fig 1:**
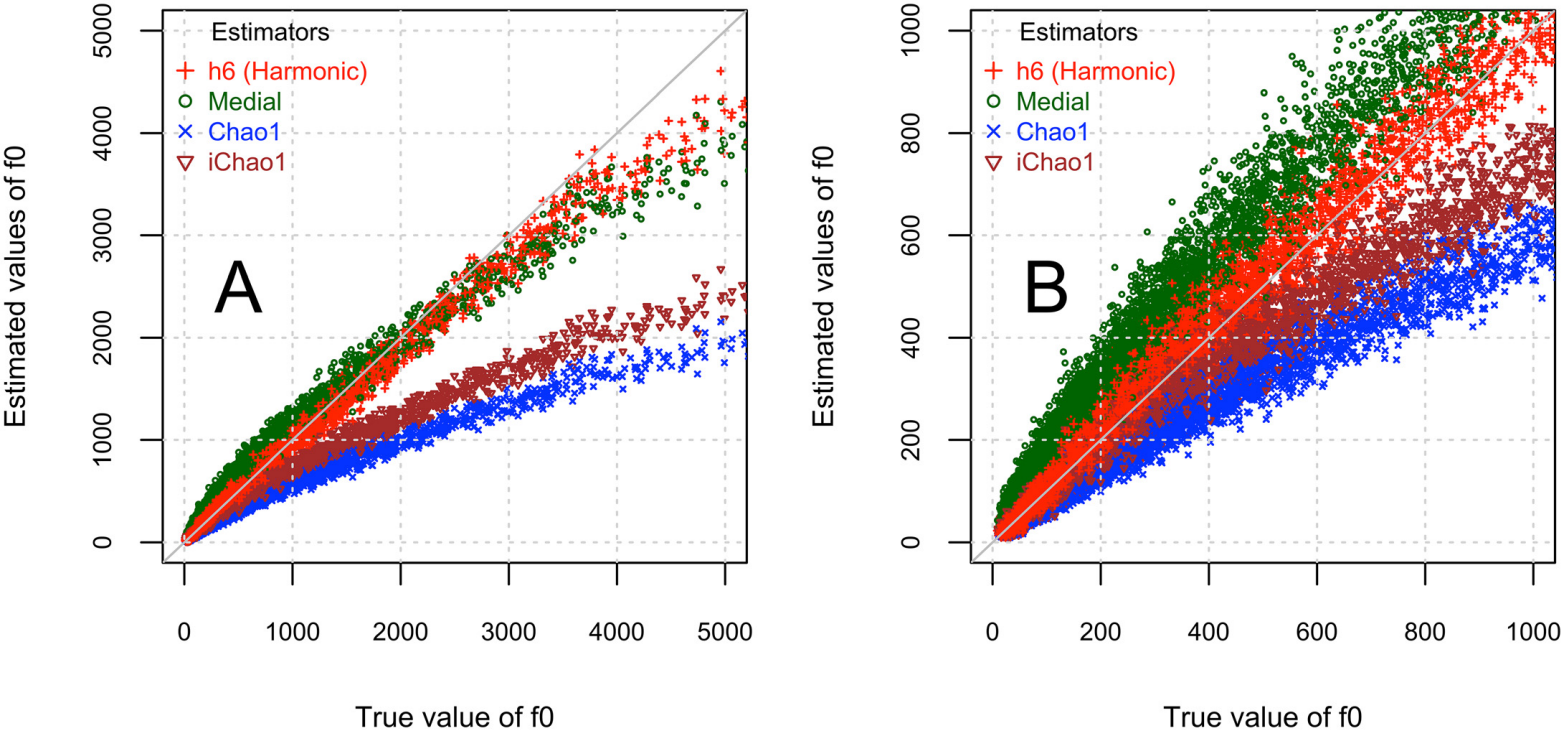
Scatterplot of true (*f*_0_;*X* axis) and estimated (${\hat{f}}_{0};Y$ axis) values for four estimators. Values of 10000 true and estimated values, ($f_{0},{\hat{f}}_{0}$) using four estimators (harmonic of degree 6, *h*_6_ in red, Medial in dark green Chao1 in blue and iChao1 in brown), in random samples of the complete dataset (accession GSE1581). Sample sizes vary uniformly between 1 and 160.5 million tags. Panel **A** presents the plot in the complete intervals, while panel **B** presents a close-up including only the values $f_{0}\leq 1,000;{\hat{f}}_{0}\leq 1,000$.

To appreciate the behavior of the estimators, Fig 1 presents a scatter plot of the true value of *f*_0_ *versus* the estimated values, ${\hat{f}}_{0}$, using the four estimators in a random subset of 10000 of the 100000 points analyzed. Panel **A** presents the full range of true *f*_0_ values, while panel **B** presents only values up to 1000 for the pairs $\left\{f_{0},{\hat{f}}_{0}\right\}$. All four estimators tend to underestimate the value of *f*_0_ when this value is large, say, when the number of missing genes is larger than approximately 3000 (value of 3000 in the X-axis of Fig 1). This happens when the sample size, say, *N*_*i*_, is relatively small in comparison to the size of the complete sample, **N** = 160552086. For example, values of *f*_0_ ≥ 3000 were obtained by sample sizes *N*_*i*_ ranging from a minimum of approximately one million (0.6% of **N**) up to 3.5 million (2% of **N**) and a mean of 2.3 million (1.4% of **N**). The complete range of variation in *N*_*i*_ extends from up to 160.5 million, with a mean of 81 million. Even in such small sample sizes, for example between 0.6 and 2% of the complete sample, the least biased estimator is *h*_6_ when comparing (Fig 1). In Panel **B** of Fig 1 we examine the behavior of the estimators in large sample sizes, when the true value of *f*_0_ ≤ 1000. These points correspond to cases where the sample size *N*_*i*_ is between 14 and 160.5 million, representing between 9% and 100% of the original sample size **N**. In these cases, *h*_6_ behaves consistently better than the Chao1, iChao1 and Medial estimators, by having estimated values closer to the value ${\hat{f}}_{0}=f_{0}$ which is indicated in both panels of Fig 1 by a grey line. In summary, from Table 1 and Fig 1 we conclude that the *h*_6_ estimator is more effective than the Chao1, iChao1 and Medial estimators. More detailed analyses, including comparisons with other putative estimators, are found in Sections C and F of S1 File.

### Validation of the *h*_6_ estimator in independent datasets

It could be argued that the estimator *h*_6_ was tailored for a specific (complete) dataset, and thus *a priori* there is no guarantee that the behavior of *h*_6_ will be preserved in different RNA-seq datasets. Although the sequencing depth (**N**) of RNA-seq experiments has been growing due to advances in high throughput sequencing technologies, we were unable to discover additional examples of complete RNA-seq samples in the public databases in order to further test the estimators. In other words, we did not find other publicly available data in which the criterion *f*_1_ = 0 was fulfilled. However, we found three datasets near completion in which *f*_1_ was small, and consequently the estimated number of missing genes, ${\hat{f}}_{0}$, is likely to be small by any of the estimators proposed. We repeated the evaluation of the Chao1, Medial and all putative *f*_0_ estimators -including *h*_6_, in these three datasets.

The three almost complete datasets selected to verify the behavior of the *h*_6_ estimator were a comprehensive study of the human transcriptome by MPSS [60] which has been also re-analyzed by us when defining parameters including transcriptome diversity and specialization [61], and the accessions E-GEOD-38298 (using the fungus *Candida albicans*) and E-GEOD-46953 (using *Mus musculus*). In each case the datasets were subjected to the same procedure as the one explained above with the complete sample; details of the procedure as well as extra analyses can be consulted in Section C of S1 File.

Table 2 presents the main characteristics of the almost complete accessions employed to validate the *h*_6_ estimator.

**Table 2:** Statistics for three RNA-seq datasets.

Accession (dataset)	*N*	*g*	*f*_1_	${\hat{f}}_{0}$ Estimates			Standard Errors of ${\hat{f}}_{0}$		
				Chao1	Medial	${\hat{h}}_{6}$	Chao1	Medial	${\hat{h}}_{6}$
Human MPSS	31,411,949	22,935	3	0	1	0	929	498	358
E-GEOD-38298	35,973,307	6,096	9	1	2	7	41	27	25
E-GEOD-46953	415,562,392	18,752	40	8	16	25	248	111	95

Statistics for three RNA-seq datasets including estimated standard errors, $se({\hat{f}}_{0})$ for the Chao1, Medial and *h*_6_ estimators. Table presents sample sizes, *N*; observed number of genes, *g*; values of *f*_1_ as well as values of *f*_0_ estimated in the datasets (columns 5 to 7; ${\hat{f}}_{0}$ for each estimator, rounded figures) and values of the standard errors of $\hat{f}$ for Chao1, Medial and *h*_6_, obtained from *B* = 100,000 bootstrap replicates (columns 8 to 10); see text and details in S1 Table.

We also examined the standard error of the estimators, evaluated in each case using *B* = 100000 bootstrap samples, exploring a range of uniformly distributed sample sizes going from approximately one million up to the corresponding values of the sample sizes, **N**, in the original accessions. The sample sizes employed in the three studies are heterogeneous in that the first two (human MPSS and E-GEOD-38298) employed from 31 to 36 million tags, while the mouse accession E-GEOD-46953 employed a sample more than 12 times larger, of approximately 415 million tags. Conversely, the number of genes detected, *g*, is approximately 23000 and 19000 in the human MPSS and mouse accessions (E-GEOD-46953) respectively, while *g* is approximately 6000 for the fungus *Candida albicans* (accession E-GEOD-38298). The values of the numbers of singletons, *f*_1_, in the accessions are relatively small: 3, 9 and 40 for each accession. The estimated values for the number of missing genes in Table 2, ${\hat{f}}_{0}$, vary depending of the estimator employed. For the human MPSS accession, we have *f*_1_ = 9, *f*_2_ = 27, *f*_3_ = 15, *f*_4_ = 23, *f*_5_ = 439, *f*_6_ = 346, which result in the values 3^2^/(2 × 9) = 0.5 ≈ 0, 3^2^/9 = 1 and (6/10)3^2^/*H*(9, 27, 15, 23, 439, 346) = 0.2814167 ≈ 0 for the Chao1, Medial and *h*_6_ estimates respectively. In general, we confirm that the three accessions are near completion, in that they fail to report on the expression of only a small number of genes: 0, 7 and 25 for the human MPSS, E-GEOD-38298 and E-GEOD-46953 respectively, using the *h*_6_ estimate (column 7). In Table 2 the estimated standard errors of the estimators, $se({\hat{f}}_{0})$, are consistently smaller for the *h*_6_ estimator when compared with the Chao1 and Medial estimators in the same accession. In the human MPSS accession the $se({\hat{f}}_{0})$ of the Chao1 and Medial estimators are 929/358 ≈ 2.59 and 498/358 ≈ 1.39 larger than the one for *h*_6_, while for the accession E-GEOD-38298 these figures are 41/25 ≈ 1.64 and 27/25 ≈ 1.08 and in the E-GEOD-46953 accession we have 248/95 ≈ 2.61 and 111/95 ≈ 1.17. The reductions in standard error when employing *h*_6_ instead of the Chao1 or Medial estimators imply that the *h*_6_ estimator results in a less biased and more robust estimation of the parameter of interest over a large range of sample sizes and conditions (see Section C in S1 File for more details of the comparisons). Our estimator, *h*6, also resulted better than the iChao1 estimator in all comparisons performed; see details in Section F of S1 File.

### Discussing the validity and optimality of *h*_6_

A possible objection to the *h*_6_ estimator is that the procedure to obtain it was purely heuristic, i.e., without employing analytical statistical theory either exact or asymptotic. Our modeling approach can be justified by the intractability of the exact moments for *f*_*r*_; *r* > 0 without making assumptions about the distribution or even under any reasonable assumed distribution. Technical difficulties arise from the impossibility of deciding on a single reasonable distribution for *G*, the number of expressed genes, in each and every RNA-seq experiment that can be performed. From the pioneering work of Fisher [62], who proposed the Poisson series and negative binomial distributions, to modern approaches [47] that use the log normal distribution or even [23] which proposes mixtures of distributions, particular samples do not always follow a specific parametric model, and thus the non-parametric framework appears more sensible. Within transcriptomes, as well as in ecological communities, a few transcripts (or species) are particularly abundant, whereas most are rare. In large assemblages such as the complete samples used here, there are more rare species than the log normal model predicts (see [23] for an ecological example). Another interesting model is presented in [63], where the authors postulate a Pareto-like probability function for gene expression, which appears to be invariant among eukaryotic cell types. However, this model predicts an unlimited increase in the number of species (i.e. distinct genes) as the sample size approaches infinity, and thus this empirical parametric approach is not useful for the estimation of *f*_0_.

Our approach to obtain the estimator *h*_6_ explored a limited number of functional forms, given in (Eq 5) and motivated by the Chao1 estimator (Eq 2). We demonstrate that the harmonic mean of *f*_2_, *f*_3_, ⋯, *f*_6_ includes valuable additional information that is not used by the Chao1, iChao1 or Medial estimators, giving a less biased and more robust estimator. Logically, we cannot guarantee that *h*_6_ is the best of all putative estimators for all possible RNA-seq samples; its optimality is naturally restricted to the functions explored, and was validated with independent RNA-seq datasets.

### Estimation of the number of undetected expressed genes in public datasets

The problem of estimating the number of expressed genes that remain undetected in RNA-seq experiments is largely irrelevant if this figure is either zero or very small in most RNA-seq experiments. This is an intuitive possibility, given that the number of gene tags obtained with current high-throughput sequencing technologies is large, ranging from hundreds of thousands up to hundreds of millions. However, the estimated number of missing genes in public RNA-seq experiments can be in the order of thousands, even when large sample sizes are employed. This source of uncertainty can lead researchers to falsely conclude that some genes are not expressed in a given tissue or condition.

We explored RNA-seq experiments deposited in the GEO [53] and ArrayExpress [54] public databases, and downloaded a total of 31 accessions which consisted of files with counts of reads per gene. Additionally, we included files from a sunflower experiment conducted in our laboratory [64]. Each accession included a variable number of libraries that in total yielded 311 vectors of gene counts (see Methods). In RNA-seq experiments there exists no homogeneous criterion for what constitutes a ‘gene’; i.e., what is to be taken as the unit of expression. For example, some studies take different splicing variants of transcripts derived from the same locus as different ‘genes’ for measuring expression, while in other cases all splicing variants are taken as a single ‘gene’. Alternatively, all close paralogs can be grouped as the same ‘gene’ [1]. It is important to take into account that when we are estimating undetected genes we are doing so in the particular framework of a given RNA-seq experiment and that it is difficult to make a general inference, even for the same species, using different datasets. Given the different definitions of ‘gene’ in different studies, it could be more precise to talk about the estimation of ‘undetected classes’; however, for consistency, we will keep discussing the concept as ‘undetected genes’.

To study the number of undetected genes not only in each one of the individual libraries, but also in the full accession or total library, we collapsed the data for all libraries in each accession in a single sample, adding the tags by gene; i.e., the count for gene *i* in the total library, $y_{i}^{t}$, was calculated as

$$\begin{array}{c} y_{i}^{t}=\sum_{j=1}^{j=v}y_{ij} \end{array}$$

where *y*_*ij*_; *i* = 1, 2, ⋯, *g*; *j* = 1, 2, ⋯, *v* are the counts for gene *i* in library *j* and *v* is the total number of libraries for the accession. This procedure was possible for 31 of the 32 accessions, given that one of the accessions had not the same genes identifiers in the library files, and thus these libraries were analyzed only independently. By collapsing all libraries of one accession in a single total library, we are analyzing all genes that were detected in such a collection, some of which could be present only in some of the individual libraries. This total library is not usually analyzed by the researchers, given that the aim of many experiments is to detect differential gene expression between treatments (sets of libraries). However the total library contains all genes detected in the experiment, and by estimating the number of undetected genes in this library we can estimate the total number of relevant entities that were missing.

Table 3 presents the results of the analysis of undetected genes for 31 total libraries in the same number of accessions.

**Table 3:** Statistics for the ‘total’ libraries for 31 accessions from different organisms.

							95% Conf. Int. *h*_6_		
Row	Accession	Organism	*N*	*g*	${\hat{h}}_{6}$	$se({\hat{h}}_{6})$	Lower	Upper	%*h*_6_/*G*
1	GSE1581	*Mus musculus*	160.6	23,332	0	5	0	9	0
2	HumanMPSS	*Homo sapiens*	31.4	22,935	1	2	0	5	0
3	E-GEOD-38298	*Candida albicans*	36.0	6,096	7	6	0	20	0
4	Sunflower	*Helianthus annuus*	579.7	36,314	23	5	13	33	0
5	E-GEOD-46953	*Mus musculus*	415.6	18,752	25	8	9	41	0
6	E-GEOD-48862	*Sus scrofa*	404.8	22,534	38	17	5	71	0
7	E-GEOD-38435	*Drosophila melanogaster*	150.5	24,293	39	8	23	55	0
8	E-GEOD-43667	*Sus scrofa*	258.0	22,419	53	23	8	98	0
9	E-MTAB-1178	*Mus musculus*	496.9	27,982	137	12	113	161	0
10	E-GEOD-51091	*Neurospora crassa*	101.4	9,269	289	21	248	331	3
11	E-GEOD-34914	*Homo sapiens*	314.0	20,422	291	25	242	340	1
12	E-GEOD-27971	*Tetrahymena thermophila*	64.6	23,770	383	24	336	429	2
13	E-GEOD-44171	*Sus scrofa*	228.5	20,857	521	31	460	583	2
14	E-GEOD-48147	*Bos taurus*	108.8	17,677	1,250	44	1,163	1,337	7
15	E-GEOD-40285	*Mus musculus*	30.8	19,885	1,576	53	1,471	1,680	7
16	E-GEOD-45474	*Mus musculus*	371.0	20,998	1,613	52	1,511	1,715	7
17	E-GEOD-37544	*Bos taurus*	38.2	16,920	1,680	57	1,569	1,791	9
18	E-GEOD-53024	*Homo sapiens*	141.9	32,471	1,760	56	1,651	1,870	5
19	E-GEOD-56890	*Mus musculus*	53.2	17,424	1,761	54	1,656	1,867	9
20	E-GEOD-42960	*Homo sapiens*	89.9	18,593	1,881	56	1,772	1,991	9
21	E-GEOD-47735	*Mus musculus*	54.4	21,370	1,914	61	1,794	2,034	8
22	E-MTAB-651	*Homo sapiens*	191.6	18,429	2,050	61	1,931	2,168	10
23	E-GEOD-29992	*Mus musculus*	28.1	21,446	2,429	63	2,305	2,553	10
24	E-GEOD-29162	*Glycine max*	31.9	39,013	2,752	66	2,624	2,881	7
25	E-GEOD-16868	*Zea mays*	10.0	21,602	3,421	76	3,272	3,571	14
26	E-GEOD-16789	*Zea mays*	5.4	24,743	4,270	83	4,107	4,433	15
27	E-GEOD-29163	*Glycine max*	257.3	54,644	4,295	84	4,130	4,460	7
28	GSE54123	*Capsicum annuum*	8.0	34,066	4,786	113	4,565	5,008	12
29	E-GEOD-29134	*Glycine max*	103.8	48,306	5,403	95	5,217	5,588	10
30	E-GEOD-33793	*Physarum polycephalum*	2.4	16,331	5,588	111	5,370	5,807	25
31	E-GEOD-44384	*Homo sapiens*	546.2	31,375	7,131	111	6,913	7,349	19
							**95%*h*_6_ Conf. Int.**		
		**Statistic**	***N***	***g***	**${\hat{h}}_{6}$**	**$se({\hat{h}}_{6})$**	**Lower**	**Upper**	**%*h*_6_/*G***
		Minimum	2.39	6,096	0	2	0	5	0
		Median	103.84	21,602	1,613	53	1,511	1,715	7
		Average	171.44	24,331	1,851	48	1,757	1,944	6
		Maximum	579.73	54,644	7,131	113	6,913	7,349	25
		Standard deviation	172.31	10,065	1,980	34	1,915	2,043	6

*N*—Sample size in millions, *g*—Number of genes detected, ${\hat{h}}_{6}$—Estimated number of missing genes, $se({\hat{h}}_{6})$—Estimated standard error for ${\hat{h}}_{6}$, 95% approximated confidence intervals for ${\hat{h}}_{6}$ (lower and upper bounds) and estimated percentage of missing genes, %*h*_6_/*G*.

Representatives were included from a wide range of living organisms, from protozoa (*Tetrahymena thermophila*), fungi (*Candida albicans*, *Neurospora crassa*), slime molds (*Physarum polycephalum*), a brown alga (*Saccharina japonica*, Table 4), plants (*Zea mays*, *Glycine max*, *Capsicum annuum*, *Helianthus annuus*), insects (*Drosophila melanogaster*) up to mammals (*Homo sapiens*, *Mus musculus*, *Sus scrofa* and *Bos taurus*). The sample size, *N*, varied in the range of 2.39 to 579.73 million tags, with a median of approximately 100 million tags. The number of detected genes, *g*, varied from approximately 6 thousand in the fungus *Candida albicans* to a very large value of more than 54 thousand in soybean (accession E-GEOD-29163). This wide variation in the number of genes estimated can be explained not only by differences between the genome sizes of the organisms, but also by the lack of homogeneity in the definition of the unit of measure for expression, as commented above. For the accessions GSE1581, HumanMPSS and E-GEOD-38298 (rows 1 to 3), where the number of undetected genes, ${\hat{h}}_{6}$, is estimated as 0, 1 and 7 respectively, 95% confidence intervals for ${\hat{h}}_{6}$ include zero. These were the accessions used to test and validate the *h*_6_ estimator. In contrast, for the majority of the accessions (rows 14 to 31) the estimated number of undetected genes is larger than one thousand, indicating that a substantial proportion of the expressed genes, ranging from 7% to 25% of the total number of expressed genes, remained undetected by RNA-seq. The last column of Table 3 (%*h*_6_/*G*) presents the percentage of undetected genes with reference to the total, say $100\times {\hat{h}}_{6}/\hat{G}=100\times {\hat{h}}_{6}/(g+{\hat{h}}_{6})$. This percentage ranges from approximately zero for nine accessions (rows 1 to 9) up to 25% for the accession E-GEOD-33793 (row 30), and has an estimated median and average of 7% and 6%, respectively. From these analyses we can infer that, on average, existing RNA-seq experiments fail to detect approximately 7% of the genes expressed in the organisms studied with the sample sizes usually employed. Note that there is no positive correlation between the sample size, *N*, and the estimated number of undetected genes, ${\hat{h}}_{6}$, the estimated value being *r* = −0.1721. For example, one of the accessions with a large sample size (*N* > 546 million, row 31) is one with a large number of undetected genes (${\hat{h}}_{6}=7,131$). Conversely, two of the samples considered to be complete (rows 2 and 3) were obtained with small samples, *N* = 31.4 and 35 million, respectively. The discordance between the number of undetected genes estimated in the two human accessions, HumanMPSS and E-GEOD-44384, (rows 2 and 31 in Table 3) can be explained by the fact that different units of measure were taken as ‘genes’. In the first case (HumanMPSS, row 2, [60]) canonical human genes were used as units of expression, while in the second (E-GEOD-44384, row 31, [65]), a study of RNA methylation targets, small RNAs are the units of measure, i.e., the ‘genes’. Finally, from Table 3 we note that the standard errors of ${\hat{h}}_{6}$ (column $se({\hat{h}}_{6})$), estimated by the bootstrap procedure are relatively small, $2\leq se({\hat{h}}_{6})\leq 34$, leading to small 95% approximate confidence intervals for this parameter (but see Analysis and Section D in S1 File).

**Table 4:** Statistics for individual libraries of 32 accessions group by organism.

Row	Organism	#Acc.	#Lib.	*avg*(*g*)	*h*_6_ Estimates			%*h*_6_/*G* Estimates		
					min.	avg.	max.	min.	avg.	max.
1	*Heliantdus annuus*	1	7	32,735	524	775	1,235	2	2	4
2	*Saccharina japonica*	1	2	65,645	336	1,430	2,523	2	2	4
3	*Candida albicans*	1	4	6,059	108	136	158	2	2	3
4	*Capsicum annuum*	1	8	21,168	19,809	21,482	23,145	50	50	52
5	*Tetrahymena thermophila*	1	6	20,518	559	1,291	1,510	6	6	7
6	*Drosophila melanogaster*	1	8	23,639	3,056	3,680	4,020	13	13	15
7	*Neurospora crassa*	1	5	8,868	247	396	474	4	4	5
8	*Physarum polycephalum*	1	3	7,747	265	3,115	6,887	22	22	38
9	*Bos taurus*	2	35	12,733	1,360	1,804	3,817	13	13	33
10	*Zea mays*	2	4	21,492	3,086	4,063	4,863	16	16	18
11	*Sus scrofa*	3	36	18,876	44	1,108	2,619	6	6	15
12	*Glycine max*	3	17	40,901	2,663	5,136	7,280	11	11	15
13	*Homo sapiens*	6	77	15,391	0	1,747	10,247	7	7	29
14	*Mus musculus*	8	99	14,317	14	1,871	11,466	10	10	50
	Total	32	311	17,501	0	2,433	23,145	10	10	52

#Acc.—Number of accessions, #Lib.—Number of libraries, *avg*(*g*)—Average number of detected genes per library, and minimum (min.), average (avg.) and maximum (max.) for the values of missing genes, ${\hat{h}}_{6}$, and estimated percentage of missing genes, %*h*_6_/*G*.

Table 4 presents a summary of the statistics for undetected genes in the individual libraries of each accession, grouped by organism.

This table includes data derived from 14 organisms, 8 of them (rows 1 to 8) represented by a single accession and the remaining represented by a minimum of 2 and up to 8 accessions. The number of libraries analyzed by organism ranges from 2 for *Saccharina japonica* (a brown algae, row 2), up to 99 for mouse (*Mus musculus*, row 14). The minimum, average and maximum values for *h*_6_ and %*h*_6_/*G* represent the variation within an organism. For the cases where more than one accession was analyzed (rows 9 to 14), the variation between accessions can be very large, as discussed above. From the sets of libraries representing a single accession (rows 1 to 8), the one with the largest average number of undetected genes is the one corresponding to chili pepper (*Capsicum annuum*, row 4, [1]), having an average of 21482 missing genes that represent approximately 50% of the total number of estimated genes. However, the number of genes detected in this accession when the individual libraries were amalgamated (total; row 28 of Table 3) was 34066 and the estimated number of undetected genes in that total library was 4786, representing only 12% of the total genes. This large difference between analyses of total and individual libraries regarding undetected genes is explained by the presence of specific genes that are expressed only in one of the libraries or conditions studied within an accession.

### Estimation of the extra sample needed for a comprehensive coverage

Having an estimation of the number of genes that remain undetected in a given sample, say ${\hat{f}}_{0}$, we can calculate the extra sample size (increased sequencing depth), say *m*_*ψ*_ (given in gene tags), needed to increase the number of observed genes from the value of *g* in the current sample up to $g+\psi {\hat{f}}_{0}$, where *ψ* is a proportion 0 < *ψ* < 1. In [52] the authors propose that the size of the extra sample, *m*_*ψ*_, can be obtained by a numerical procedure including bootstrap, and yields as approximate solutions to the formula

$$\begin{array}{c} m_{\psi }\approx N\frac{f_{1}}{2f_{2}}\log(\frac{{\hat{f}}_{0}}{\hat{G}\left(1-\psi \right)}) \end{array}$$ (8)

where *f*_1_, *f*_2_ are, as before, the estimated numbers of singletons and doubletons in the sample, ${\hat{f}}_{0}$ is the estimated number of undetected genes (Chao1, iChao1 or Chao2 in this context), and $\hat{G}=g+{\hat{f}}_{0}$ represents the estimated total number of genes or classes.

We found that a more realistic value for *m*_*ψ*_ in the case of RNA-seq is given by

$$\begin{array}{c} m_{\psi }^{\prime }=N\frac{h_{6}}{f_{1}}\log(\frac{h_{6}}{\hat{G}\left(1-\psi \right)}) \end{array}$$ (9)

where *h*_6_ is our harmonic estimator of degree 6 for *f*_0_ and consistently, $\hat{G}=g+h_{6}$, is the estimate of the total number of genes using the estimator *h*_6_. Eqs 8 and 9 are subjected to the condition ${\hat{f}}_{0}/(\hat{G}(1-\psi ))>1$ to give positive values of the extra sample size. In general the researcher will be interested in values of *ψ* near 1, for example *ψ* = 0.95, 0.99, etc.

Note that Eqs 8 and 9 return a value of *m*_*ψ*_ = 0 when the sample is complete, i.e., when *f*_1_ = 0 and thus ${\hat{f}}_{0}=0$, and diverge to infinity when *ψ* = 1, indicating the impossibility to obtain a sample in which can be assured that there will be no undetected genes. Section B in S1 File presents a derivation of the probability of non-missing genes for samples of different sizes under different conditions.

To compare the performance of *m*_*ψ*_ and $m_{\psi }^{\prime }$ (Eqs 8 and 9), we obtained a large set of bootstrap replicates from the complete sample (accession GSE1581), with sample sizes ranking from 0.5 to 10 million tags and calculated the predicted and realized gain in number of extra genes observed. We found that $m_{\psi }^{\prime }$ is much more accurate and precise than *m*_*ψ*_ to calculate the extra sample size needed. The weighted square error for $m_{\psi }^{\prime }$ was approximately 50% smaller that the one for *m*_*ψ*_ in the same samples, thus we conclude that $m_{\psi }^{\prime }$ must be preferred over *m*_*ψ*_ for the estimation of the extra sample size. Details of the process to compare these estimators are presented in Section E of S1 File.

S1 Table presents the extra sample needed to obtain 95% of the number of undetected genes employing *m*_*ψ*_ and $m_{\psi }^{\prime }$ (columns “m_Chao” and “m_h6”, respectively) for the cases where ${\hat{f}}_{0}/(0.05\times \hat{G})>1$, i.e., when the condition to use the functions is fulfilled. In all comparable cases the estimation of undetected genes by Chao1 (column “Chao1”) is smaller than the estimation using ${\hat{h}}_{6}$ (column “h6”) and consequently the estimation of the extra sample size using the Chao1 estimator (column “m_Chao”) is always smaller that the estimation of extra sample size using $m_{\psi }^{\prime }$ (column “m_h6”). On average, the ratio of extra sample sizes, $m_{\psi }^{\prime }/m_{\psi }$ is approximately 18, while the ratio of estimated undetected genes ${\hat{h}}_{6}$/Chao1 is around 2. Given that the Chao1 estimator of undetected genes frequently underestimates the target parameter, our estimator of extra sample size needed to complete the sample, $m_{\psi }^{\prime }$, returns a more realistic value (see Section E of S1 File).

Current methods of RNA-seq analysis allow researchers to carry out one or more sequencing runs for the same library. A reliable estimate of the number of undetected genes and extra sample needed to observe a given proportion of the undetected genes can be employed to decide, in an informed way, whether additional sequencing runs of existent libraries are needed or not. The first sequencing run of a RNA-seq library can be used as a ‘pilot’ test to decide if more sequencing runs are needed. For example, in our laboratory we performed an RNA-seq experiment exploring changes in the transcriptome of chili pepper fruit during development [1]. In that case we estimated that there are between 4565 and 5008 genes that remained undetected in the sequencing libraries (Accession GSE54123, see Table 3). We calculated that, to observe 95% of those undetected genes, approximately 9 million additional sequences (see S1 Table) would be required. In contrast, for a gene expression study in sunflower that was also performed in our laboratory [64], only a small number of genes remained undetected (between 13 and 33 by a 95% confidence interval for ${\hat{h}}_{6}$ in row “Sunflower” in Table 3).

The analyses presented in S1 Table and summarized in tables 3 and 4 can be used as a guide for sequencing depths required by RNA-seq experiments. In particular, if the experimental aims and design, as well as sequencing technology and bioinformatic pipeline are similar to the ones used in the datasets we analyzed, our results provide guidelines for the sample size needed in future studies.

## Conclusions

The problem studied here is to decide if the genes observed in an RNA-seq library are in fact all the ones expressed, or if there is certain number of expressed genes that were not observed in the sample (missing or undetected genes). The estimation of the number of undetected genes is an essential question, both, to conclude that an unobserved gene is in fact not expressed in a given condition, as well as to predict the sample size (sequencing depth) needed for an RNA-Seq experiment.

We present a non-parametric estimator, *h*_6_, of the number of genes that remain undetected in RNA-seq experiments that is superior to the estimators previously reported. We demonstrate that *h*_6_ is less biased and consequently has a smaller standard error than the Chao1, iChao1 and Medial estimators for a wide range of sample sizes in the context of RNA-seq. We also present a function to estimate the extra sample size needed to observe a given proportion of the undetected genes that is more precise and accurate than the function presented in [52].

By analyzing a total of 342 vectors of gene counts from 32 accessions (311 individually sequenced libraries plus the total vectors for each one of 31 accessions) we conclude that there are very few RNA-seq studies that can be considered as complete, defined as experiments in which all genes are detected. On average we estimate that, given the sequencing depths currently employed in most RNA-seq studies, approximately 6% of genes per accession and 10% of the genes per library within an accession are undetected.

The statistical tools presented here will help to evaluate the inferences of RNA-seq analyses by estimating the completeness of the samples obtained and helping to decide if extra sampling is needed.

## Analysis

### Datasets

The RNA-seq data analyzed here was downloaded from the NCBI GEO [53, 66] and EMBL ArrayExpress [54, 67] repositories. The inclusion criterion for the data consisted of raw data for gene tag counts ordered by ‘gene’ (where ‘gene’ was an identifier). Accessions found with count data for sequences (instead of ‘genes’) or in which the counts were normalized were rejected. This resulted in the selection of 30 accessions from 14 different organisms with a total of 272 gene count vectors. Additionally we included two more RNA-seq experiments, the previously reported set of human MPSS data [60, 61] comprising gene counts for 32 human tissues, and a study of the sunflower transcriptome, which comprised 7 libraries [64]. The full dataset therefore included 32 RNA-seq experiments with a total of 311 individual libraries (see Tables 3 and 4 and S1 Table). All these data were input into a relational database and processed with R [68] to form ‘data frame’ objects in which genes are presented in rows and columns represent individual libraries.

### Design and selection of the *f*_0_ estimators

The functional form of the putative *f*_0_ estimators, presented in Eq 5, was motivated by the estimator Chao1, presented in [37], which uses information contained in only *f*_1_ and *f*_2_. We reasoned that additional information about undetected genes exist in the frequencies of frequencies *f*_*r*_ where *r* = 3, 4, ⋯, 10. To specify putative estimators we systematically substituted the function *c*() in Eq 5 by the arithmetic, geometric or harmonic means of *f*_2_ to *f*_*r*_; *r* = 3, 4, ⋯, 10. This yielded a set of 3 × 8 = 24 putative estimators to be evaluated (see Section C in S1 File). Other functional forms were also evaluated, but the results were unfavorable, and thus they are not presented.

To test the putative estimators of *f*_0_ we employed the total count of the accession GSE1581 [55–59], which has a sample size **N** = 160, 552, 086 gene tags. In this experiment, the number of expressed genes detected was 23332 and can be considered complete by having *f*_1_ = 0; i.e., all genes were represented by at least two gene tags. In this complete sample we set *G* = 23332 and thus if we take a subsample and observe the number of genes obtained, *g*, the true number of missing genes in that subsample, say, *f*_0_ = *G* − *g* = 23332 − *g*, and using the observable frequencies *f*_1_, *f*_2_, ⋯,, we can try all putative estimators, ${\hat{f}}_{0}$, calculating in each case the error of each estimator, ${\hat{f}}_{0}-f_{0}$ and, by repeating this process a large number of times, estimate the standard error of each estimator using Eq 6. The process of resampling was carried out in *B* = 100,000 subsamples obtained by assuming the multinomial distribution and sample sizes, *N*, uniformly distributed between 1 and 160.5 million tags. The estimator with better statistical properties, including a smaller standard error, was the harmonic estimator of degree 6, *h*_6_ (Eq 7). The use of *h*_6_ was validated using other nearly complete datasets. Details of the selection and validation process, including extra tables and figures, are presented in Sections C of S1 File. All analyses were performed in R [68].

### Design and testing of the estimator of extra sample size $m_{\psi }^{\prime }$

To design an estimator for the extra sample size, $m_{\psi }^{\prime }$, required to observe a proportion *ψ* of the estimated missing genes, $\psi {\hat{f}}_{0}$, we first tried substituting the estimator of *f*_0_ (Chao1) in Eq 8 by our estimator *h*_6_. However, by trying other functional forms of the quotient *f*_1_/2*f*_2_ in Eq 6 we obtained the quotient *h*_6_/*f*_1_ which is part of Eq 9 and yielded better results than the original equation presented in [52]. To test different functional forms of the estimator of extra sample size we used the weighted squared error, defined as

$$\begin{array}{c} se\left(m_{\psi }\right)={\left(\frac{g+\psi {\hat{f}}_{0}|N-E\left[G|N+m_{\psi }\right]}{{\hat{f}}_{0}}\right)}^{2} \end{array}$$

where $g+\psi {\hat{f}}_{0}|N$ is the number of genes predicted by the estimator and *E*[*G*∣*N*+*m*_*ψ*_] is the expected number under the distribution of the data. *se*(*m*_*ψ*_) was evaluated for a large set of bootstrap samples with sample sizes, *N*, uniformly distributed between 0.5 and 10 million tags. Section E in S1 File presents the details of the results obtained.

### Analyzes of public RNA-seq datasets

All R datasets containing the gene counts (see ‘Data’ above) were processed in R [68] to obtain the basic statistics of the samples, punctual estimations of missing genes by the Chao1, iChao1, Medial and *h*_6_ estimators, as well as standard error and 95% approximate confidence limits for ${\hat{h}}_{6}$ (see Section D of S1 File for details of the method to obtain the approximate confidence limits). S1 Table presents the full results.

### Software to calculate *h*_6_ and related statistics

An R [68] function, ‘h6’, included here as S1 Text, was programed and tested, implementing the estimation of ${\hat{f}}_{0}$ by our estimator *h*6, as well as related statistics, including approximate standard error, bias and confidence intervals for ${\hat{f}}_{0}$, as well as the estimate of the extra sample size needed to estimate a proportion, *ψ*, of the undetected genes, $m_{\psi }^{\prime }$. The R package ‘UndetectedGenes’, containing the function ‘h6’ as well as examples of analysis is available at **Computational Biology, Langebio**. To install the package in R type ‘R CMD install file_name’ (where ‘file_name’ is the name of the downloaded file) at the command line and in the directory where ‘file_name’ is located. After installation, to use the package in R type ‘library(UndetectedGenes);? UndetectedGenes’ at the R prompt (>). See also Section G of S1 File.

## Supporting Information

S1 File Supporting results with additional tables and figures. Sampling framework and notation (Section A). The probability of missing genes (Section B). Comparing *f*_0_ estimators (Section C). Approximate confidence intervals for *f*_0_ (Section D). Calculating extra sample needed to estimate some of the missing genes (Section E). Comparing *h*_6_ with iChao1 and other estimators (Section F). R functions (Section G).
(PDF)

S1 Table Missing genes statistics for all analyzed datasets. Presents all statistics relevant to missing genes estimation in all datasets analyzed in an Excel file.
(XLSX)

S1 Text ‘h6’ R function. A documented R function (in plain text format) for the estimation of undetected genes and related statistics.
(TXT)
