# Supplementary material for: How Many Genes Are Expressed in a Transcriptome? Estimation and Results for RNA-Seq
Source: PLoS One. 2015 Jun 24;10(6):e0130262. doi: 10.1371/journal.pone.0130262 (PMC4479379; doi:10.1371/journal.pone.0130262)
Supplement: S1 File — Sampling framework and notation (Section A). The probability of missing genes (Section B). Comparing f 0 estimators (Section C). Approximate confidence intervals for f 0 (Section D). Calculating extra sample needed to estimate some of the missing genes (Section E). Comparing h 6 with iChao1 and other estimators (Section F). R functions (Section G). (PDF) [file pone.0130262.s001.pdf]

# - SUPPORTING INFORMATION 'S1 FILE' - (STATISTICAL RESULTS, TABLES AND FIGURES) FOR '*HOW MANY GENES ARE EXPRESSED IN A TRANSCRIPTOME? ESTIMATION AND RESULTS FOR RNA-SEQ*'

LUIS FERNANDO GARCÍA-ORTEGA AND OCTAVIO MARTÍNEZ*
LANGEBIO - CINVESTAV - MÉXICO
*CORRESPONDING AUTHOR `OMARTINE@LANGEBIO.CINVESTAV.MX`

ABSTRACT. The estimation of missing genes, or in general missing classes, is a very interesting and difficult problem given that we are trying to calculate the number of undetected parameters from the data observed in the current sample. Here we present details of the derivations for the statistical formulas, as well as simulations and other analyses performed to support the results and conclusions of our paper. All calculations were performed in R [11], and all functions, data and results are available from the corresponding author (OM) upon request. We also present the '`UndetectedGenes`' R package.

Contents

A. Sampling framework and notation 1
A.1. Difficulties to evaluate $P[f_r = j]$ 2
A.2. Data 3
B. The probability of missing genes 3
B.1. The homogeneous model 4
B.2. Heterogeneous model: real transcriptomes 5
B.3. Sample size needed to obtain $P[f_0 > 0] = \alpha$ in real cases 6
C. Comparing f_0 estimators 9
C.1. Testing the estimators of f_0 using a complete sample 10
C.2. Comparing the selected estimators 13
C.3. Validating h_6 as estimator of f_0 22
C.4. Conclusions about the validation of h_6 30
D. Approximate confidence intervals for f_0 33
D.1. Properties of bootstrap estimates of f_r 33
D.2. Unsuitability of Bootstrap Percentile Confidence Intervals for h_6 35
E. Calculating extra sample needed to estimate some of the missing genes 38
E.1. Comparing m_ψ with m'_ψ in subsamples of a complete dataset 38
F. Comparing h_6 with iChao1 and other estimators 40
G. R functions 44
G.1. sample.Pf0 44
G.2. The R package '`UndetectedGenes`' 46
References 46

A. Sampling framework and notation

We are interested in the calculation of the number of *undetected (or missing) genes* in an RNA-seq sample. For this we will assume that the source of RNA is in a steady state in which there is a fixed number of mRNA molecules, each one belonging to a particular gene in a set indexed by $i = 1, 2, \cdots, G$ where G is the total number of different genes expressed in the source. The '*source*' could be a single cell, or a set of cells of the same kind, or a tissue or an organ, etc. The number of m-RNA molecules

for each gene i is given by the vector $\mathbf{X} = (X_1, X_2, \cdots, X_G)$ and $L = \sum_i X_i$ denote the total number of mRNA molecules in the source, thus the probability of obtaining a gene of the class i when sampling at random is given by $p_i = X_i/L;\ i = 1, 2, \cdots, G$.

The methodology employed in RNA-seq experiments implies that we will sample *with replacement* a large number, N, of *gene tags*, which are small DNA molecules, which can be univocally related with their gene of origin, say i. To simplify the framework, but without loss of generality, we will assume that all mRNA are of the same size, and thus the probability of obtaining a gene tag from gene i is p_i and it stay constant during the sampling procedure. We define $\mathbf{p} = (p_1, p_2, \cdots, p_G)$ as the vector of probabilities. If we want to consider the length of each mRNA molecule and the constant size of the gene tags, then this framework can be modified, but this do not alter the statistics involved[1].

Let's begin by assuming that the total number of genes being expressed, G, is known. By sampling at random and without replacement a fixed number of gene tags, N, and classifying (mapping) each tag to its gene of origin, we obtain a random vector $\mathbf{Y} = (Y_1, Y_2, \cdots, Y_G)$ where each Y_i is the count obtained for a gene i and the realized values y_i could be zero, and are constrained by the condition $\sum_i Y_i = N$. Under these conditions it is clear that the distribution of $\mathbf{Y}$ is multinomial of parameters $\{N, \mathbf{p}\}$ and the maximum likelihood estimators (MLE) of p_i are $p_i = Y_i/N$.

However, if we do not know the value of G, then we can only assure that a gene of the class i is expressed in the source when the observed value $y_i > 0$; otherwise this class remains invisible in a given sample. Keeping the notation of the observed vector as $\mathbf{y}$, but taking into account the fact that G is unknown, for a particular sample we obtain observed values $\mathbf{y} = (y_1, y_2, \cdots, y_g)$ where now all observed values $y_i > 0$ and the number of observed classes, g, is the realization of a random variable G. Note that even when using the same letter for the subindex, i, the vectors of realizations when G is known and unknown are indexed differently. Also note that the distribution of G depends in a complex way on N and $\mathbf{p}$, and also that, as shown by Good in [6], the values $p_i = y_i/N$ are no longer MLE of the corresponding parameters.

In the main text we explained the change of notation to consider the observed values of f_r, the frequency of frequency r or, more clearly, the number of cases where $y_i = r$. Using an indicator function this can be written as

$$f_r = \sum_{i=1}^{i=g} I_r(y_i)$$

where $I_r(y_i) = 1$ if $y_i = r$ and $I_r(y_i) = 0$ otherwise, $i = 1, 2, \cdots, g;\ r = 1, 2, \cdots$, and it is clear that

$$N = \sum_{i=1}^{i=g} y_i = \sum_{r=1}^{r=\infty} r f_r$$

We denot as f_0 the number of undetected or *missing* genes; genes that are being expressed with a probability larger than zero, but that are not present in the sample. Clearly then

$$f_0 = G - g$$

where G is the total number of genes and g is the observed value in the sample, but G is unknown and f_0 is no directly observable from the sample.

A.1. **Difficulties to evaluate** $P[f_r = j]$**.** The change of notation from the counts of observed in the sample, $y_i,\ i = 1, 2, \cdots, G$ to the frequencies of frequencies, $f_0, f_1, f_2, \cdots$, is done by convenience, given that criteria for 'completeness' of the sample are better explained, as stoping rules, in this notation. This notation makes also possible to write down the estimators of f_0. However, if one wants to treat f_r as random variables, an extra parameter must be taken into account apart from $\mathbf{p}$ (which includes G), and this is a fixed sample size, N. In other words, for fixed values of all $\mathbf{p}$ the probability function of f_r is

[1] If the length of each gene, say $n_1, n_2, \cdots, n_G$, is taken into account then the probabilities must be weighted by the corresponding lengths, becoming $p_i = n_i X_i/R;\ R = \sum_i n_i X_i$.

dependent on $\{\mathbf{p}, N\}$. We will see later that it is easy to calculate $P[f_0 > 0|\mathbf{p}, N]$; however, in general to write down the exact probabilities $P[f_r = j|\mathbf{p}, N]$, $r = 0, 1, 2, \cdots,\ ; j = 0, 1, 2, \cdots$, is difficult because it involves large combinatory calculations which are numerically specific for each one of the many possible pairs $\{r, j\}$. To see this difficulty consider the calculation of $P[f_r = j|\mathbf{p}, N]$ for a single fixed pair of natural numbers $\{r, j\}$ when the probability function of the vector $\mathbf{Y}$ is multinomial, which is the natural choice of distribution. The calculation of $P[f_r = j|\mathbf{p}, N]$ involves to find the set Υ of all possible cases where exactly j of the Y_i variables are equal to r and all other $G - j$ variables take any value different to r, but always fulfilling the condition $\sum_i Y_i = N$.

$$\Upsilon = \{\mathbf{Y} \in \mathbb{N}^G | r(Y_i \equiv j), \sum_i Y_i = N\}$$

where $r(Y_i \equiv j)$ means '*exactly r of the Y_is are equal to j and all permutations are allowed*'. Then we can write

$$P[f_r = j|\mathbf{p}, N] = \sum_{\mathbf{y} \in \Upsilon} P[\mathbf{Y} = \mathbf{y}|N, \mathbf{p}]$$

where

$$P[\mathbf{Y} = \mathbf{y}|N, \mathbf{p}] = \frac{N!}{\prod_i y_i!} \prod_i p_i^{y_i}$$

is the multinomial probability. The problem for numerical evaluation is, of course, to find for each $\{r, j\}$ the set of values $\mathbf{y} \in \Upsilon$. The cardinality of Υ is of course finite, but very large for even moderate values of G and N; we have that

$$\Upsilon \subset \mathbb{N}^G | \sum_i Y_i = N$$

and the number of elements in $\mathbb{N}^G | \sum_i Y_i = N$ is given by the combinatorial

$$\begin{pmatrix} N + G - 1 \\ G - 1 \end{pmatrix}$$

and thus exact numerical calculations are out of reach.

A.2. **Data.** Data in RNA-seq are vectors of gene counts in which each element is a natural number larger than zero. However, in many cases more than one library is sequenced, and then the data can be re-arranged from the previous notation into a matrix $\mathbf{y} = \left[\mathbf{y}^1, \mathbf{y}^2, \cdots, \mathbf{y}^v\right]$, where each one of the $\mathbf{y}^j$, $j = 1, 2, \cdots v$ are the columns corresponding the the gene count in the j library. This matrix of order $r \times v$ contains the counts for all genes detected in all libraries. Note that in that case an element $y_{ij} \in \mathbf{y}$ can be equal to zero, denoting that the gene i was not detected in library j. Adding the rows of the matrix $\mathbf{y}$ we obtain the vector of total counts for each gene, say $\mathbf{y}^t$ where

$$y_i^t = \sum_{j=1}^{v} y_{ij}$$

We can perform the analysis of missing genes for each one of the individual libraries, $\mathbf{y}^j$, as well as for the vector of totals, $\mathbf{y}^t$, or on any convenient sum of columns, depending at what level the inference is wanted. For example, in many cases there are libraries which are replicates of the same 'treatment' (condition, tissue, etc.). In that case, if the researcher is interested in the number of missing genes in that particular treatment, the analysis must be performed on the sum of all (technical or biological) replicates of the treatment, etc. The use of a vector or matrix of data will be made clear by the context.

B. The probability of missing genes

To calculate the probability of obtaining missing genes in a sample, say $P[f_0 > 0]$, or more exactly $P[f_0 > 0|N,\ \mathbf{p}]$ we will assume that G is known, and thus f_0, the number of counts that are zero is observable. We could consider a fixed sample size N, however it is more convenient, and realistic, to assume that N is not fixed, but that it is a random variable which expectation is N. In fact, in any RNA-seq experiment the researcher do not know in advance the value of N, but only an approximate value, determined by the sequencing technology. Thus, instead of considering our random variable $\mathbf{Y}$ to have a multinomial distribution of parameters $\{N,\ \mathbf{p}\}$ we will consider G independent random variables with Poisson distribution, each one with parameter $\lambda_i = Np_i = NX_i/L$, i.e.,

$$P[Y_i = y] = \frac{e^{-\lambda_i}\lambda_i^y}{y!}$$

and we have that $E[Y_i] = \lambda_i = Np_i$ and $E[\sum_i Y_i] = \sum_i \lambda_i = N\sum p_i = N$.

We can write

$$P[f_0 > 0|N,\ \mathbf{p}] \approx P[f_0 > 0|\lambda, N] = 1 - P[f_0 = 0|\lambda, N]$$

where $\lambda = (\lambda_1, \lambda_2, \cdots, \lambda_G)$ is the vector of parameters of the Poison distributions and we have changed from a multinomial to a set of independent Poisons distributions. Setting the sample size N in the conditionals of the probability simply means that we stop counting after obtaining a total sample of size N, even when in practice this never happens. We can think of this value more in terms of $N = E[N]$.

By notting that the event '$f_0 = 0$' happens if and only if all realized values of Y_i are larger than zero, and using the independence of the Poisson variables, we obtain

$$P[f_0 > 0|\lambda, N] = 1 - P[Y_1 > 0 \cap Y_2 > 0 \cap \cdots \cap Y_G > 0|\lambda] = 1 - \prod_{i=1}^{i=G} P[Y_i > 0|\lambda_i]$$

and we obtain

(B-1) $$P[f_0 > 0|\lambda, N] = 1 - \prod_{i=1}^{i=G}(1 - e^{-\lambda_i})$$

B.1. **The homogeneous model.** To see how N affects $P[f_0 > 0] = P[f_0 > 0|\lambda, N]$ in practical cases, we must give values to the G parameters of the vector λ. To do so we can consider *homogeneous* cases where for each i we have $\lambda_i = 1/G$; i.e., cases where all classes (genes) have the same probability of expression, or equivalently, they are being expressed at the same rate. This is completely unrealistic for RNA-seq (as well as for almost any ecological setting), because a rule of the thumb is that very frequently there are 'many rare genes (species) and few very abundant ones'; however the homogeneous model will serve as a first approximation to the behavior of $P[f_0 > 0]$.

Under this homogeneous case we have only two parameters, G and $\lambda_i = \lambda = 1/G$ for all values of i, and if we sample N tags we have $E[Y_i] = N\lambda = N/G$ for each $i,\ i = 1, 2, \cdots, G$ and our expression simplifies to

(B-2) $$P[f_0 > 0|G, N] = 1 - (1 - e^{-N/G})^G$$

If we want to simplify even more, we can assume that we are obtaining samples of exactly the same size than the number of classes, say that we have $N = G$, then

$$P[f_0 > 0|G = N] = 1 - (1 - e^{-1})^G$$

It is evident that this function very quickly converges to 1 as G increases; for example for $G = N = 20$ we have $P[f_0 > 0|G = N] = 0.9998962 \approx 1$. Figure B-1 presents the plot for the first 20 values of $P[f_0 > 0|G = N], G = 1, 2, \cdots, 20$.

The parameterization given in equation B-2 for the homogeneous model, shows that the convergence of $P[f_0 > 0]$ to its asymptotes (zero and one) is governed by the ratio N/G; i.e., by the relative sample size

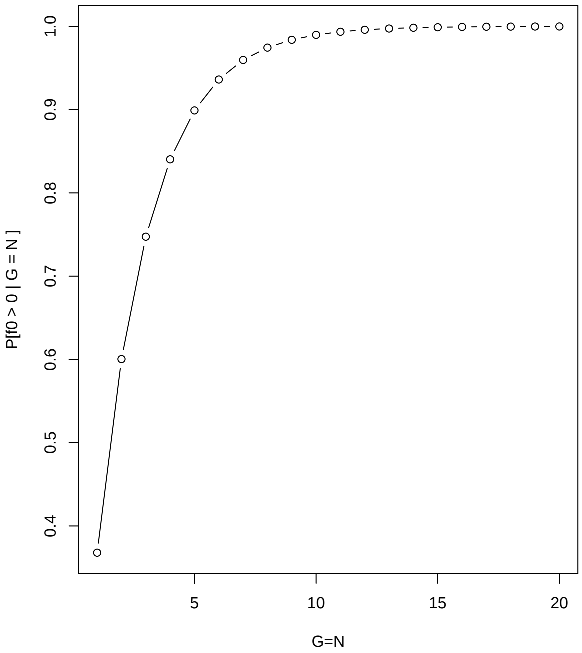

FIGURE B-1. Values of the probability of more than zero missing genes, $P[f_0 > 0]$ for $G = 1, 2, \cdots, 20$ in the case where the number of tags sampled, N, is the same than the number of genes expressed, G; $P[f_0 > 0|G = N]$.

with reference to the number of equally expressed genes. Figure B-2 presents the family of curves for $P[f_0 > 0]$ obtained by varying the ratio N/G in the homogeneous model between 0.1 and 20 and G in arbitrary steps between 10 and 40,000.

From Figure B-2 we can see how the convergence of $P[f_0 > 0]$ to zero when N/G increases depends on G; as G increases, a larger value of N/G is needed for $P[f_0 > 0]$ to be near zero. In practical cases, we want to have a sample large enough to decrease the probability of missing genes to an acceptable value, say for example $P[f_0 > 0] \approx 0.05, 0.01 \cdots$. In RNA-seq the number of expressed genes vary roughly between 5,000 and 30,000. Figure B-3 presents a zoom of Figure B-2 in the more interesting neighborhood (ratio N/G between 9 and 16 and G between 1,000 and 40,000). From Figure B-3 we can see that for $G = 1,000$ in ratios $N/G \geq 12$ we have $P[f_0 > 0] \leq 0.05$ while for $G = 40,000$ a ratio $N/G > 14$ is needed to achieve $P[f_0 > 0] < 0.05$

B.2. **Heterogeneous model: real transcriptomes.** It is important to underline that the homogeneous model, where $p_i = 1/G$, is the extreme case which gives the smallest sample size needed to obtain $P[f_0 > 0] = \alpha$. In real cases, where there are many *rare* genes, i.e., genes with $p_i \ll 1/G$, the sample size needed to obtain $P[f_0 > 0] = \alpha$ (α small) are much larger. A useful concept introduced by us in [10] is the *number of effective genes*, $\mathcal{G} = 2^H$, where $H = -\sum_i p_i \log(p_i)$ is the estimated diversity of the transcriptome defined in [9] for transcriptomes. $\mathcal{G}$ represents the number of genes equally expressed required to produce a given diversity value H. In general, real transcriptomes are far from the homogeneous model of maximum H, by having many rare genes and a few with large probabilities of expression. For example the complete mouse sample (GSE1581) has $g = 23,332$ expressed genes. The maximum diversity attainable with this number of genes is $\max(H) = \log_2(23,332) = 14.51$; however this sample has an estimated diversity of $h = 8.1$, around 55.83% of the total, and this sample has an estimated number of effective genes $\mathcal{G} = 2^{8.1} \approx 275$. The percentage of the estimated value of diversity, H with reference to the putative maximum, $\max(H) = \log_2(g)$, can be defined as the *Informatics efficiency* of the transcriptome, and denoted by $\mathcal{I} = H/\max(H)$. We will see how the large number of rare genes in real transcriptomes

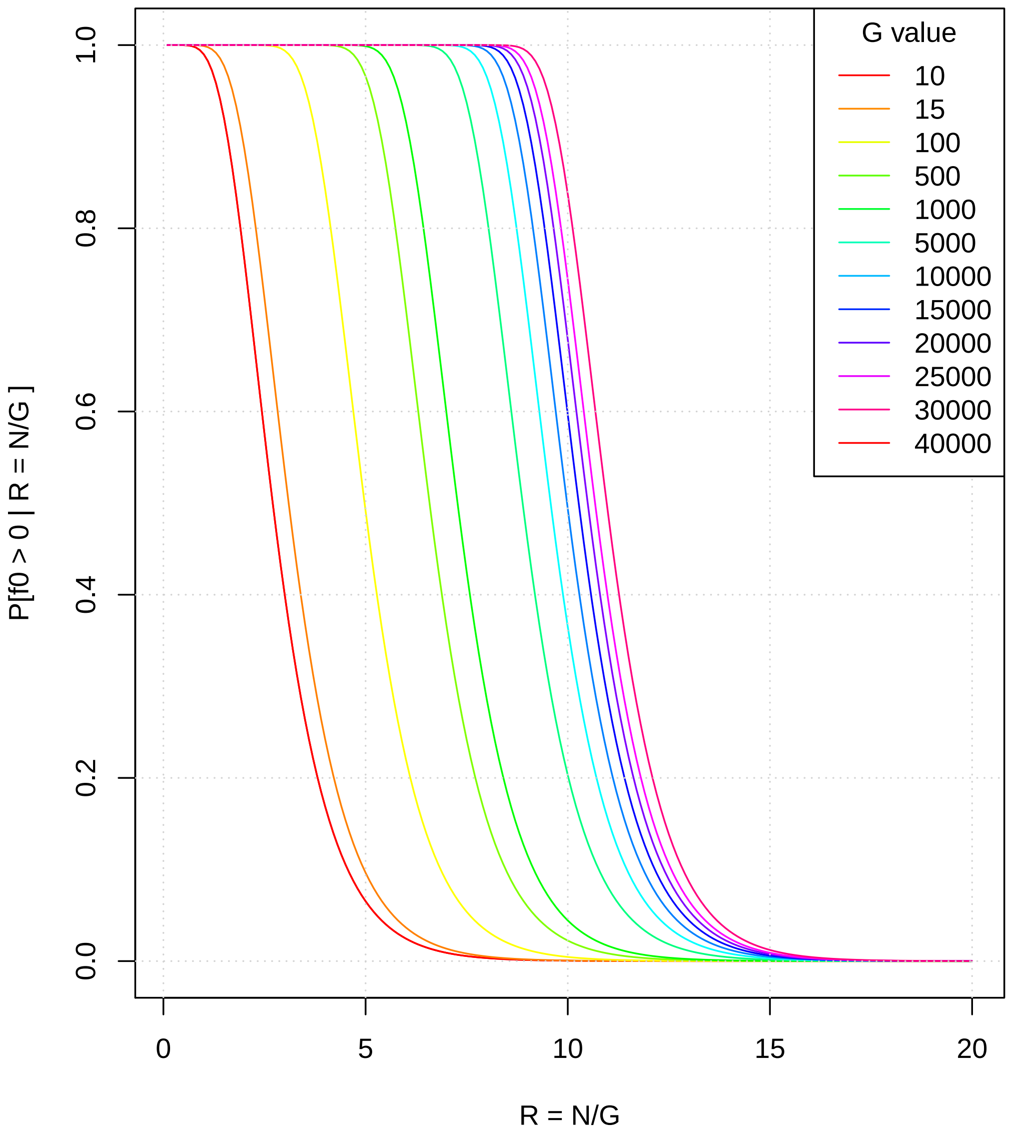

FIGURE B-2. Values of the probability of more than zero missing genes, $P[f_0 > 0]$ for $G = 1, 2, \cdots, 20$ when the value of the ratio N/G vary between 0.1 and 20 for different values of genes expressed, G.

implies that much larger samples than the ones needed under the (unrealistic) homogeneous model are needed to obtain small probabilities of having missing genes, i.e., small values of $P[f_0 > 0]$.

Table B-1 presents the statistics for the accessions studied. This table was obtained for the '*total*' tags per gene; i.e., for the vectors obtaining by adding by gene all libraries sequenced in each accession (see Analysis in main text).

From Table B-1 we can see that the sample sizes employed in the accessions, N, vary from less than 3 up to more than 579 million of gene tags. The accessions are highly variable in the number of detected genes, g, that goes from around 6 up to more than 54 thousand, while the number of effective genes, $\mathcal{G}$, goes from 17 up to 773 determining an informatics efficiency, $\mathcal{I}$, that ranks between 28.37 up to 62.25% with a median of 55.83% and an average of 54.57%. From these statistics we can conclude that the real transcriptomes are very far from the homogeneous model, having a large proportion of genes that are expressed at very low frequencies. By this, the homogenous model is not at all suitable to study

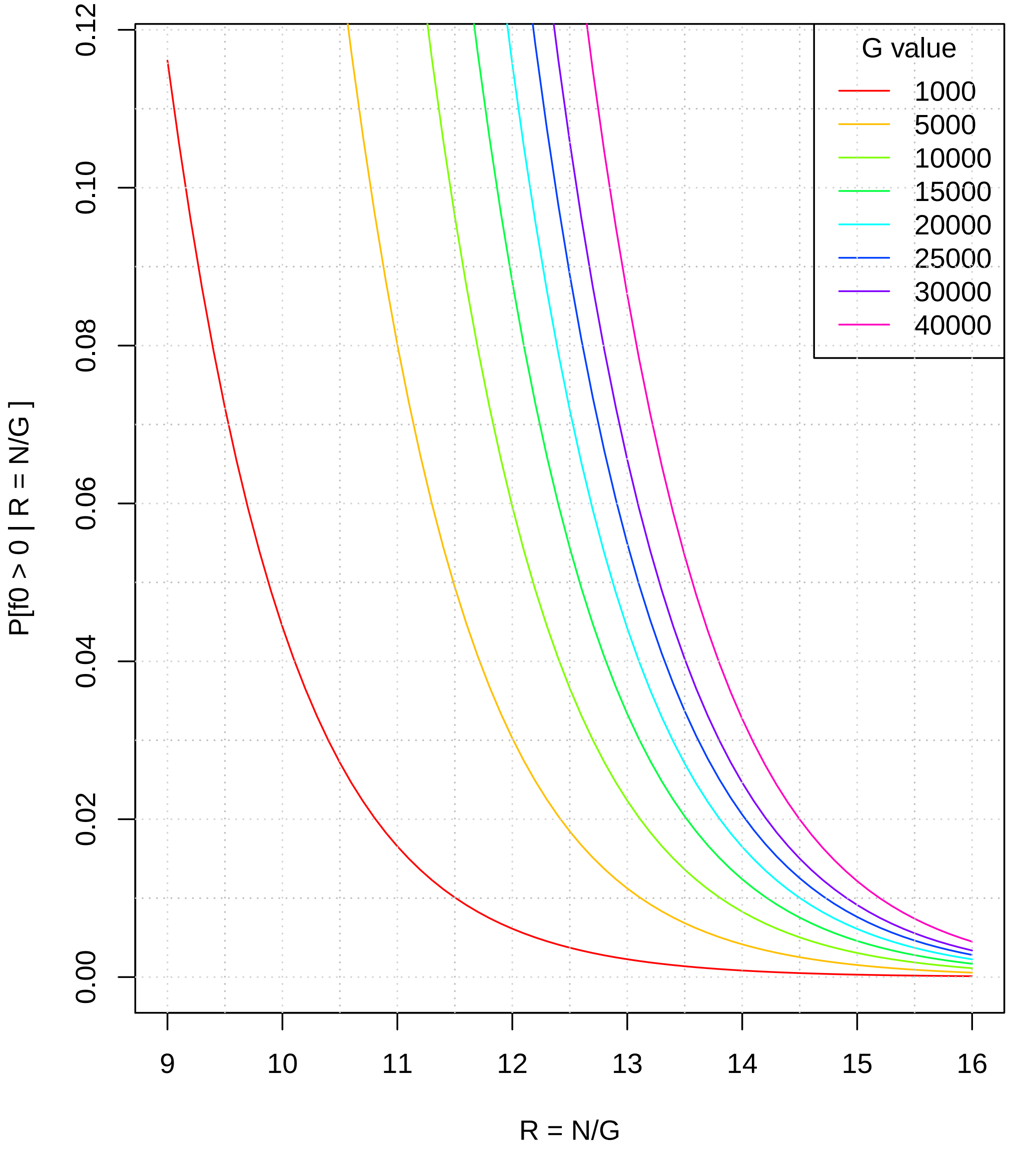

FIGURE B-3. Zoom of Figure B-2. Values of the probability of more than zero missing genes, $P[f_0 > 0]$ for $G = 1, 2, \cdots, 20$ when the value of the ratio N/G vary between 9 and 16 for different values of genes expressed, G.

transcriptomes, and thus, we must calculate the probabilities of having missing genes, $P[f_0 > 0]$, directly from the estimated probabilities of each transcriptome, without doing assumptions.

B.3. **Sample size needed to obtain $P[f_0 > 0] = \alpha$ in real cases.** In real cases, we can employ numerical methods to obtain the value of sample size, say T, that satisfies $P[f_0 > 0|\mathbf{y}, T] = \alpha$ where $\mathbf{y}$ is the original vector of counts, which estimate the probabilities of each gene, $p_i = y_i/N$, and $N = \sum_i y_i$ is the original sample size. We can calculate T by minimizing the square difference $(P[f_0 > 0|\mathbf{y}, T] - \alpha)^2$ given by

(B-3) $$m(T, \mathbf{y}, \alpha) = (1 - \prod_{i=1}^{i=G}(1 - e^{-Tp_i}) - \alpha)^2$$

Table B-1. Statistics for the total tags per accession. N - sample size (in millions), g - Observed number of genes, $\mathcal{G}$ - Number of effective genes, $\mathcal{I}$ - Informatics efficiency, T/N - Ratio of the sample size needed to achieve $P[f_0 > 0] = \alpha$ ($\alpha = 0.05$). General statistics are given in the last rows.

Accession	N	g	$\mathcal{G}$	$\mathcal{I}$ (%)	T/N
GSE1581	160.55	23,332	275	55.83	3.7
humanMPSS	31.41	22,935	217	53.60	5.2
E-GEOD-38298	35.97	6,096	118	54.76	5.2
Sunflower	579.73	36,314	284	53.81	6.1
E-GEOD-46953	415.56	18,752	406	61.05	6.7
E-GEOD-48862	404.76	22,534	379	59.25	7.4
E-GEOD-38435	150.49	24,293	295	56.33	7.5
E-GEOD-43667	257.95	22,419	313	57.36	7.7
E-GEOD-51091	101.43	9,269	201	58.04	8.0
E-MTAB-1178	496.92	27,982	284	55.18	8.2
E-GEOD-34914	313.96	20,422	418	60.82	8.5
E-GEOD-27971	64.58	23,770	225	53.77	8.9
E-GEOD-44171	228.53	20,857	163	51.20	9.0
E-GEOD-48147	108.79	17,677	146	50.99	9.6
E-GEOD-37544	38.24	16,920	429	62.25	9.7
E-GEOD-42960	89.87	18,593	130	49.51	9.8
E-GEOD-56890	53.21	17,424	109	48.09	9.8
E-GEOD-45474	370.97	20,998	235	54.85	9.8
E-MTAB-651	191.62	18,429	399	60.99	9.9
E-GEOD-53024	141.9	32,471	527	60.33	10.0
E-GEOD-29992	28.07	21,446	256	55.58	10.1
E-GEOD-40285	30.82	19,885	318	58.22	10.2
E-GEOD-47735	54.35	21,370	17	28.37	10.2
E-GEOD-16868	10.00	21,602	427	60.70	10.3
E-GEOD-29162	31.86	39,013	437	57.51	10.4
E-GEOD-29163	257.34	54,644	773	60.96	10.6
E-GEOD-16789	5.41	24,743	416	59.60	10.7
E-GEOD-29134	103.84	48,306	330	53.76	10.9
E-GEOD-44384	546.15	31,375	127	46.81	11.0
E-GEOD-33793	2.39	16,331	32	35.85	11.1
GSE54123	8.02	34,066	351	56.17	11.2
Statistic	N	g	$\mathcal{G}$	$\mathcal{I}$ (%)	T/N
Minimum	2.39	6,096	17	28.37	3.7
Median	103.84	21,602	284	55.83	9.8
Average	171.44	24,331	292	54.57	9.0
Maximum	579.73	54,644	773	62.25	11.2
S	172.31	10,065	157	7.26	2.0

In the section '*R functions*' below ,we present the function to obtain the minimum of equation B-3 while in Table B-1 we can see the results of the minimum sample size needed to have a value $P[f_0 > 0] = 0.05$, expressed as the ratio T/N.

Note that in all these calculation we are *not* including any estimate of the number of missing genes, i.e., we are assuming that the sample is 'complete', and the calculated sample sizes, T, are under this (unrealistic) assumption.

From Table B-1 we can see that even for the more '*complete*' samples, where the number of missing genes is estimated to be very low (see main text), the sample sizes needed to obtain a small probability of missing genes are very large, going from 3.7 up to 11.2 times the original sample employed in the studies. The average sample size employed in the accession samples is around 171.44 million tags, while the average of the ratio T/N is around 9, thus, the average sample size needed in RNA-seq studies to have a low probability of missing genes, $P[f_0 > 0] = 0.05$, can be roughly estimated to be around $171 \times 9 \approx 1,539$ million tags. Even when this figure is not too large for the current sequencing technologies, it is certainly much larger than the number of tags regularly employed in sampling a single library or even a set of libraries in RNA-seq; for example, the maximum number of tags employed in a single accession is of around 580 millions (Table B-1).

A putative use of the ratio T/N in RNA-seq studies is to indicate to the researcher how much larger the sample size need to be to have an almost complete sample, in which the probability of missing genes is small. Note however, that the ratio T/N do not uses estimates of the number of missing genes, $\hat{f}_0$, and thus refers only to the current realized sample. In fact, when calculating T/N we are assuming that the sample is *complete*, i.e., that there are not missing genes and we are calculating T/N based on that assumption, which can be considered realistic only if the sample is near completion, i.e., if in fact the number of missing genes is relatively small. In the practical side, if the estimated ratio T/N is '*large*', say > 5, the researcher need to be aware of the fact that the probability of be missing genes in his/her sample is very large, i.e., near 1 and thus could consider to increase the sample size or at least be careful in the inferences for genes not found in a given sample. For example, only in 4 accessions (GSE1581, humanMPSS, E-GEOD-38298 and Sunflower) the probability of having $f_0 > 0$ with the employed sample size, say $P[f_0 > 0|\mathbf{y}, N]$, was slightly smaller than one; i.e., in all libraries studied (342) of all accessions (32), we calculated $P[f_0 > 0|\mathbf{y}, N] \approx 1$.

C. Comparing f_0 estimators

In the main text we propose for putative estimators of the number of missing genes the functional form

(C-4)
$$\hat{f}_0 = u \, \frac{f_1^2}{c(f_2, f_3, \cdots, f_{10})}$$

where the constant u is an scalar to be determined and the function $c()$ is a measure of central tendency for $f_2, f_3, \cdots, f_{10}$ or a subset of these quantities (equation 5 in main text). This functional form is based in the Chao1 estimator, $f_1^2/2f_2$ [1], under the rational that the small frequencies of frequencies $f_3, f_4, \cdots, f_{10}$ contain extra information about f_0 which is not captured by f_2, and thus in the denominator of the f_0 estimator we will try to summarize that information by means of a measure of central tendency. As measures of central tendency, $c()$, we will use the Pythagorean means of $\{f_2, f_3, \cdots, f_k\}$ for $k = 2, 3, \cdots, 10$, i.e., we will evaluate the function with the arithmetic, geometric or harmonic means of the small frequencies. We will use the following notation; the arithmetic mean of degree r is given by

$$\bar{f}_r = \frac{1}{r-1} \sum_{k=2}^{k=r} f_k$$

for $r = 2, 3, \cdots, 9$, thus $\bar{f}_r$ is the average of the frequencies f_2 up to f_r. In a similar way the geometric mean of order r is defined as

$$\hat{f}_r = \left(\prod_{k=2}^{k=r} f_k \right)^{\frac{1}{r-1}}$$

while the harmonic mean of order r is given by

$$\tilde{f}_r = (r-1) \left(\sum_{k=2}^{k=r} \frac{1}{f_k} \right)^{-1}$$

Taking into account that r can take 9 different values (from 2 to 10) we can define in principle $3 \times 9 = 27$ different putative estimators, substituting $c()$ in B-1 by each one of the measures of central tendency $\bar{f}_r, \hat{f}_r, \tilde{f}_r;\ r = 2, 3, \cdots, 10$. However, $\bar{f}_2 = \hat{f}_2 = \tilde{f}_2 = f_2$ (because we have a single value to estimate the 'central tendency'), and thus for $r = 2$ we have the same estimator, say f_1^2/f_2, which is the Medial estimator presented in [12]. Thus, we have a total of $1 + (3 \times 8) = 25$ different forms of B-1 to test. We will denote the estimators that use the arithmetic average of order r as 'a_r', the ones that use the geometric mean as 'g_r' and the ones that use the harmonic mean as 'h_r', thus for example

$$h_4 = u\frac{f_1^2}{\tilde{f}_4}$$

and so on.

It is a well known result that, for any set of positive numbers in which at least two are different, the arithmetic mean, $\bar{f}_r$, is always the largest, the harmonic mean, $\tilde{f}_r$, always the smallest and the geometric mean, $\hat{f}_r$, is somewhere between, say $\bar{f}_r \geq \hat{f}_r \geq \tilde{f}_r$, thus, for the proposed functions (general equation B-1) we have that any estimated values in a given sample and with the same order estimator we will have $a_r \leq g_r \leq h_r$. Since the harmonic mean tends strongly toward the smallest elements of the set, it tends (compared to the arithmetic mean) to mitigate the impact of large outliers and aggravate the impact of small ones. Given that with the values of $f_r, r = 2, 3, \cdots$, we are measuring frequencies of occurrences of the values r in a dataset, it is doubtful that the arithmetic mean could give the best representation of "central tendency" by these kind of data, and likely the harmonic mean can give a better result representing the *speed* with which the frequencies decrease as r increases.

Here we will select an estimator for f_0 from the set proposed above by taking into account desirable statistical properties.

C.1. **Testing the estimators of f_0 using a complete sample.** The analytical evaluation of the proposed estimators is impossible without knowing the true distribution of G, the number of genes expressed in each particular condition (RNA-seq experiment), and even if that distribution could be defined, the distributions of the frequencies of frequencies, $f_0, f_1, f_2, \cdots$ given an observed value of $G = g$ result intractable without doing some asymptotic approximations. Thus, to empirically test different estimators of f_0 we can evaluate the estimated standard error,

(C-5)
$$se(\hat{f}_0) = \sqrt{\frac{1}{B}\sum_{i=1}^{i=B}(\hat{f}_{0i} - f_{0i})^2}$$

(equation 6 in main text) in which the pairs $\{\hat{f}_{0i}, f_{0i}\}$ are the estimated and true values of the parameter in sample i, respectively. The evaluation of B-2 for all the set of putative estimators must be performed in samples that mimic as realistically as possible the process of obtaining gene tags in RNA-seq experiments and using a wide range of sample sizes, N. By evaluating all estimators in the same set of samples, we can compare their statistical properties, including $se(\hat{f}_0)$, and make a selection based on these properties. Knowing the true value of the number of expressed genes, say G, we will know the true value of the number of missing genes in the sample i, say $f_{0i} = G - g_i$, where g_i is the number of genes observed in the sample i, and thus we will be able to evaluate the standard error (equation C-5), as well as any other statistical properties of the estimators. For this we need a complete sample. We are going to use a dataset that can be considered to be 'complete' in the sense that there is evidence that all expressed genes, G, were observed and as a consequence we have estimation of the frequencies of expression of each gene, $p_i = y_i/N$ that are maximum likelihood estimators (MLE) of the parameters of interest. As evidence of the completeness of the sample we used the 'stoping rule' proposed in [2], that consider a sample complete when $f_1 = 0$. This complete dataset is from accession GSE1581 (`http://www.ncbi.nlm.nih.gov/geo/query/acc.cgi?acc=GSE1581`) and consist of counts for a total of $G = 23,332$ genes and a sample size $\mathbf{N} = 160,552,086$ resulting from adding the gene counts from 35 libraries (see Analysis in the main text).

For sampling the complete dataset we can assume two different models, the Multinomial distribution of G classes with parameters $p_i = y_i/\mathbf{N};\ i = 1, 2, \cdots, G$ or a set of G independent Poisson variables, each one with parameter $\lambda_i = Np_i$. In each case the value of the size of the sample, N, can be specified and the sample can be obtained by pseudorandom numbers using the corresponding R functions. The Multinomial model arise directly from applying the assumption of sampling N times with replacement a population having G classes in relative proportions $\{p_i\}$, while the Poisson model assume that each gene is expressed at a constant rate $\lambda_i = Np_i$ during the sampling. The main difference between the models is that with the Multinomial we fix N, while for the Poisson we only know the expected value of the sample, N, but the sample size is a random variable of parameter $\lambda = \sum_i \lambda_i$. The Poisson model is more 'realistic' in the sense that in practice the researcher do not know, in advance, the exact sample size that a sequencing run will provide. Other models, as the Negative Binomial, which includes a parameter for over dispersion are also reasonable, but they were not considered here. We have seen that in practice the estimated distributions of the frequencies of frequencies, $f_0, f_1, f_2, \cdots$ are not strongly affected by the selection of the sampling distribution (Multinomial or Poisson; data not shown), thus any of the two models can be used, depending if it is more convenient to have a fixed or random sample size, N, for the samples.

Note that in equation C-4 each estimator includes a scalar constant u that must be determined to complete the formula for each estimator. This constant can be approximately calculated by estimating the parameter u of the lineal model $f_{0i} = u\hat{r}_{0i} + \epsilon_i$, where $\hat{r}_{0i}$ is the part of the estimator that do not include u, say

$$\hat{r}_0 = \frac{f_1^2}{c(f_2, f_3, \cdots, f_{10})}$$

Evaluating the lineal models $f_{0i} = u\hat{r}_{0i} + \epsilon_i$ corresponding to each one of the 25 proposed estimators we can select the 'best' using statistical criteria, as for example select the one with larger lineal correlation with f_0 and smaller standard error, etc. At the same time we will obtain for each estimator an estimation of u, say $\hat{u}$ obtained by the least squares method.

As a first test of all 25 estimators, we obtained samples from the Multinomial distribution with parameters $\mathbf{p}$ equal to the relative frequencies of each gene in the complete sample and values of N varying from 100,000 to 161,000,000 in steps of 100,000, i.e., a total of 1,610 different values for N, covering a rank from 0.0006$\mathbf{N}$ up to 1.003$\mathbf{N}$, where $\mathbf{N} = 160,552,086$ is the sample size of the complete dataset. For each value of N we simulated 100 samples, giving a total of $B = 161,000$ bootstrap replicates. Table C-2 present the results for the fitting of the lineal models, $f_{0i} = u\hat{r}_{0i} + \epsilon_i$, for each one of the 25 proposed estimators.

From Table C-2 we can see that, in general, by taking into account frequencies other than f_1 and f_2 in the estimators, we improve the estimation of f_0. This can be noticed because the worst model for f_0 is the one that takes into account only f_1, f_2 and corresponds to the estimators $a_2 = g_2 = h_2$ which are identical to the Medial estimator, and lineally related to the Chao1 estimator by a constant equal to 1/2. The model corresponding to this estimator (first row in Table C-2) has the highest REE and lowest estimated values of Adj. r^2 and r. This confirms that residual information about f_0 is present in the frequencies $f_3, f_4, \cdots, f_{10}$ and, possibly in higher order frequencies. On the other hand, from Table C-2 we can see that the geometric and harmonic means give better estimators of f_0 than the estimators using the arithmetic mean; i.e., for estimators of the same order, $r = 3, 4, \cdots, 10$ we see that the values of r and Adj. r^2 are alway larger for the estimators g_r and h_r, compared with the corresponding a_r. Consequently, the values of REE, which measure the degree of fitting, are always larger for a_r when compared with g_r or h_r. Comparing estimators that use the geometric mean (g_r) with the ones that use the harmonic mean (h_r) for the same order, $r = 3, 4, \cdots, 10$, we can see that the ones using the harmonic mean are always better (higher r and Adj. r^2), even when the advantage between harmonic and geometric means is not as large as the one of these to the arithmetic means. Figure C-4 presents a scatter plot for the values of r and r^2 for each one of the estimators proposed.

TABLE C-2. Statistics for the models $f_{0i} = u\hat{r}_{0i} + \epsilon_i$ estimated from $B = 161,000$ samples from the complete sample varying N between 0.1 and 161 million tags. $\hat{u}$ - Estimated slope; $se(\hat{u})$ - Standard error of $\hat{u}$; Adj. r^2 - Adjusted r^2 of the model; REE or $\hat{\sigma}_e$ - Residual standard error of the model; CV - Estimated coefficient of variance for the estimators (standard deviation over mean); r - Pearson's correlation coefficient between f_{0i} and $\hat{r}_{0i}$

Estimator	$\hat{u}$	$se(\hat{u})$	Adj. r^2	REE	CV	r
$a_2 = g_2 = h_2$	1.14526	0.00081	0.92472	265	1.57315	0.96495
a_3	1.01643	0.00063	0.94175	233	1.67300	0.97199
a_4	0.91632	0.00051	0.95241	210	1.74431	0.97658
a_5	0.83682	0.00043	0.95987	193	1.79911	0.97990
a_6	0.77181	0.00036	0.96556	179	1.84409	0.98250
a_7	0.71752	0.00031	0.97001	167	1.88227	0.98457
a_8	0.67140	0.00028	0.97359	157	1.91557	0.98625
a_9	0.63158	0.00024	0.97657	148	1.94536	0.98767
a_{10}	0.59678	0.00022	0.97908	140	1.97235	0.98888
g_3	1.00641	0.00061	0.94408	228	1.68325	0.97315
g_4	0.89644	0.00048	0.95669	201	1.76706	0.97869
g_5	0.80815	0.00038	0.96569	179	1.83491	0.98278
g_6	0.73537	0.00031	0.97264	160	1.89352	0.98602
g_7	0.67432	0.00025	0.97799	143	1.94552	0.98855
g_8	0.62231	0.00021	0.98211	129	1.99280	0.99051
g_9	0.57729	0.00018	0.98533	117	2.03703	0.99204
g_{10}	0.53778	0.00015	0.98775	107	2.07915	0.99319
h_3	0.99637	0.00059	0.94635	223	1.69363	0.97426
h_4	0.87704	0.00044	0.96059	191	1.78958	0.98060
h_5	0.78098	0.00034	0.97062	165	1.86962	0.98518
h_6	0.60169	0.00026	0.97814	143	1.94083	0.98871
h_7	0.53535	0.00021	0.98351	124	2.00539	0.99123
h_8	0.47906	0.00016	0.98716	109	2.06524	0.99293
h_9	0.33042	0.00014	0.98941	99	2.12264	0.99395
h_{10}	0.28770	0.00012	0.99024	95	2.17920	0.99424

In Figure C-4 we can see that all three kind of estimators increase their linear relation with the increasing order, with a tendency to converge to the value $r = 1$. From this plot we can also confirm that the increase of the values of correlation for the h_r estimators as r increase is faster than for either, the g_r or a_r. From this behavior one can say that the best estimator will be $h10$ -given that it has the largest correlation with the true value of the parameter, f_0. One can also propose (and test) harmonic estimators of larger order, h_r; $r = 11, 12, \cdots$. However, a reason for not increasing further the order of the estimator is that the coefficient of variation of the estimators, CV, increases with the order, as can be seen from Table C-2. A reason to not propose a 'limit estimator', say $h_{\max(r)}$, where $\max(r)$ is the largest value of r observed in the sample, is that for large values of r we have that $P[f_r = 0]$ becomes large, and the vector $(f_r, f_{r+1}, f_{r+2}, \cdots)$ becomes sparse (data not shown). Also, the quantity of information about f_0 which exist in the values of f_r; $r > 10$ appears to be negligible in the sense that increasing r above $r = 6$ produces only marginal increases in the correlation between $f_0, \hat{f}_0$.

Figure C-5 present an scatterplot of the values of the coefficient of variation, CV, for the three kinds of estimators as function of their order. From Figure C-5 we can see that the CV off all three kinds of estimators increases with the order r of the estimator; this increase is larger for the h_r estimators than for g_r or a_r. With the limit of $r = 10$ shown in this plot, it can be seen that the increase of CV as

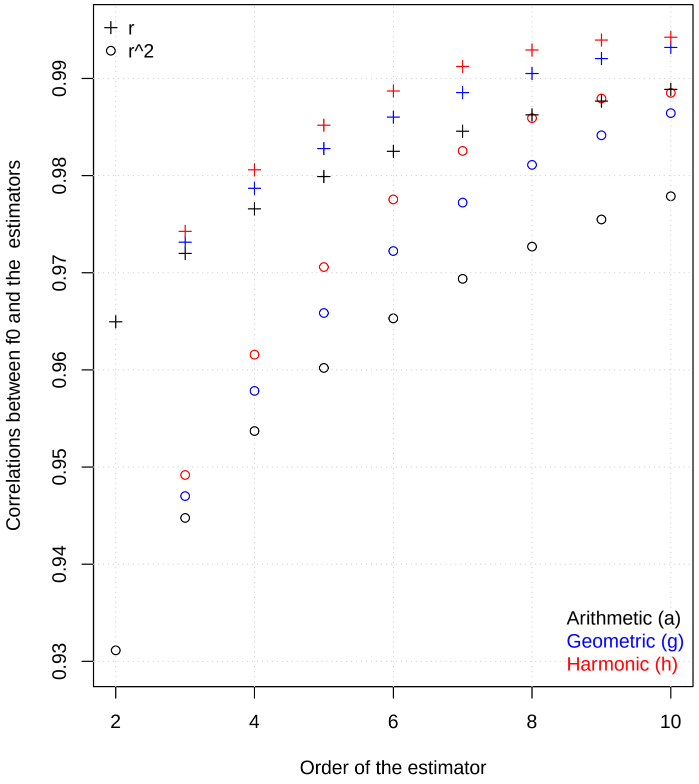

FIGURE C-4. Scatter plot of the values of the coefficients of correlation (r, plus signs) and determination (r^2, circles) between the values of f_0 and the estimators of different order (X-axis) using arithmetic (black) geometric (blue) or harmonic (read) means.

function of r for h_r is almost a straight line, while for the arithmetic estimators, a_r, it shows a tendency to asymptotic stability as r increases.

Given the results presented in Table C-2 and figures C-4 and C-4 we think that a reasonable selection for the estimator of f_0 is given by h_6. This function has a large correlation with the true parameter, $r \approx 0.99$, that do not increases very much when the order is further increased (Figure C-4) and also h_6 presents a relatively small coefficient of variance, $CV \approx 2$. As estimator of f_0, h_6 presents a relatively small standard error (measured by REE in Table C-2), and a reasonably small CV, given a good equilibrium between accuracy and precision. From Table C-2 we can see that the estimated value of the constant u for the functional form of this estimator is $\hat{u} = 0.60169 \approx 6/10$, thus the final form of the estimator that we consider as best estimator of f_0 is given by the harmonic estimator of degree 6 of f_0,

$$h_6 = \frac{6}{10}\,\frac{f_1^2}{\tilde{f}_6} \qquad \text{(C-6)}$$

where, as defined above, $\tilde{f}_6$ represents the harmonic mean of $f_2, f_3, \cdots, f_6$, and thus equation C-6 is equivalent to the formula given in the main text (equation 7) because $\tilde{f}_6 = H(f_2, f_3, \cdots, f_6)$.

In the following section we will study with more detail the behavior of h_6 compared with the Chao1 and Medial estimators of f_0.

C.2. **Comparing the selected estimators.** Having selected the harmonic estimator of degree 6, h_6, from the group of putative estimators as the ‘best’ estimator for f_0, we must make a more detailed study of of their statistical properties, comparing them with the published estimators Chao1 [1] and Medial [12], taking into account that the later differs from the first only by a constant.

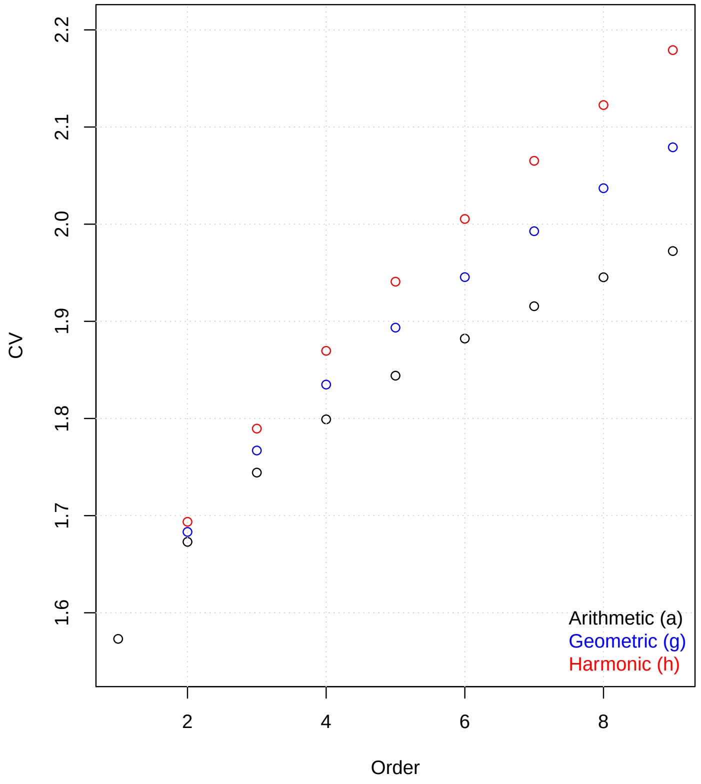

FIGURE C-5. Scatter plot of the values of the coefficients of variation, CV, as function of the order r of the estimators for f_0 using arithmetic (black) geometric (blue) or harmonic (read) means.

With this aim we will use again the complete sample, derived from accession GSE1581 and in which we have $G = 23,332$ genes and a sample size $\mathbf{N} = 160,552,086$. To simulate a more realistic situation we will employ the Poisson instead of the Multinomial model, given that the first consider the counts for each gene, say $\mathbf{Y}_i$, $i = 1, 2, \cdots, G$ as independent random variables with Poison distribution of parameters $\lambda_i = Np_i$, and thus it takes into account the variation in sample size. Now N is not fixed, but represents the expected sample size. As before, the parameters p_i are assumed to be known from the corresponding relative frequencies in the complete sample, say $p_i = y_i/\mathbf{N}$. Thus in this model, in contrast with the Multinomial, we let the sample size be a random variable, mimicking more realistically what happens in RNA-seq experiments in which the researcher does not know the final sample size in advance.

C.2.1. *Statistical properties of the quantities employed in the estimation.* Here we study the properties of the quantities $f_0, f_1, f_2, \cdots, f_6$; $g = G - f_0$ when we vary the sample size N. Given that there is a direct relation between sample size, N, and the number of missed genes (unobserved classes, f_0) it is convenient to obtain samples with an expected value of N varying in an ample range of values. This will give the opportunity to test different functions as estimators of f_0 by having large variation of sample sizes and thus large variation in values of f_0, which are known, because we know how many genes are in the complete dataset, $G = 23,332$. For these new simulations we used sample sizes uniformly distributed between 1 million and the total sample $\mathbf{N} = 160,552,086$. This rank is realistic for RNA-seq experiments employing current sequencing technologies (see Table 3 and Supporting file 'S2 Excel' in main text). We performed a total of $B = 100,000$ simulations and Table C-3 presents the statistics for the complete dataset ('*Full*' row) as well as for the 100,000 bootstrap replicates.

From Table C-3 we can see that the realized values of N in the simulation go from around 1 million up to 160.6 millions in a well uniform distribution; the mean is close to the median and the histogram of

TABLE C-3. Statistics for the complete dataset (row *Full*) and a set of 100,000 parametric bootstrap replicates. N - sample size, g - observed number of genes (classes), f_0 - Number of genes missed, and $f_1, f_2, \cdots, f_6$ - Frequencies of frequencies.

Statistic	N	g	f_0	f_1	f_2	f_3	f_4	f_5	f_6
Full	160,552,086	23,332	0	0	74	139	169	146	148
Minimum	1,012,519	17,690	8	38	68	94	99	96	93
Mean	81,109,353	22,989	343	357	334	304	274	249	226
Median	81,214,362	23,225	107	207	245	243	226	208	192
Maximum	160,557,134	23,324	5642	2595	1578	1115	897	745	623
S (sd)	45,943,974	643	643	391	257	184	141	115	97

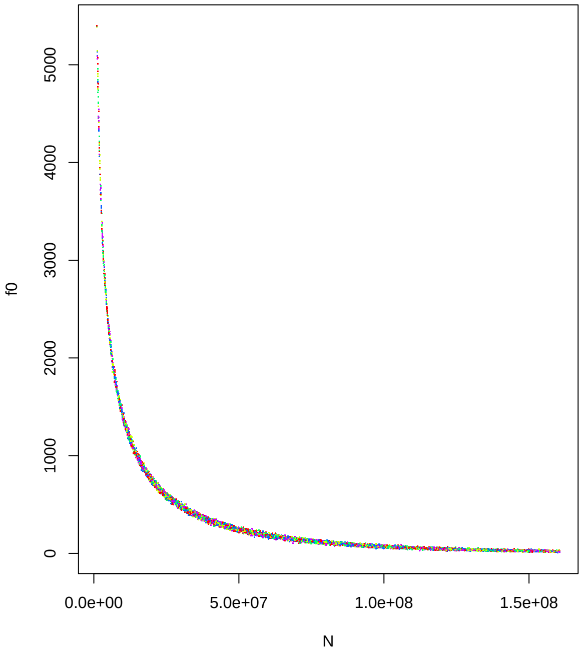

FIGURE C-6. Relation between the sample size, N, and the number of missing genes, f_0, in bootstrap samples of the complete dataset. A random set of 10,000 points, from the total of 100,000 obtained, is plotted .

N shows the uniformity of the distribution (plot not shown). The variation of N produced a large and not uniform variation in the observed number of missing genes (f_0), from a minimum of 8 to a maximum of 5,642 with a median of 107. The distribution of f_0 is highly skewed, by the fact that large number of missing genes (large f_0) are only obtained with relatively small sample sizes. Figure C-6 shows the relation between N and f_0. In Figure C-6 we can see that the relation between N and f_0 is a parabolic curve with a vertex around $N \approx 2.5e+07$, i.e., around N of 25 millions, and from that point forward the value of f_0 descends tending asymptotically to zero. On the left side of the Figure C-6 we can see that the values of f_0 will diverge to a value close to G for small values of N (i.e., $N \lessapprox 1e6$).

In Table C-3 we can observe the statistics for the values of $f_0, f_1, \cdots, f_6$, while in Figure C-7 the distributions of these values in the simulated data are shown. In Table C-3 and Figure B-3 we can see that the mean values of the f_r's decrease with r, for $r > 0$, and the same happens with the median for $r > 1$. The standard deviation, S, for the values of the r_i's decrease from $r = 0$; i.e., the most variable distribution is for f_0 with $S = 643$ and the less variable for f_6 with $S = 97$. Note that there were many outliers for f_0, the maximum being 5,642 (Table C-3) and thus the distribution of f_0 is highly skewed and variable (see Figure C-7).

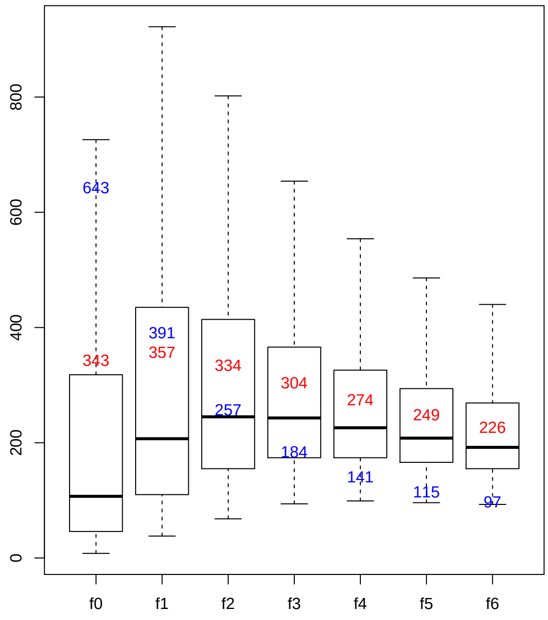

Figure C-7. Distributions as box-plots for the values of $f_0, f_1, \cdots, f_6$ obtained in the simulated samples. Many high outliers for f_0 made the value of S larger for f_0; outliers not shown in the plots. Values of means in red and standard deviations (S) in blue.

Table C-4 presents the values of correlation between some of the variables obtained from the simulation.

Table C-4. Estimated Pearson's linear correlation coefficients (r) in the set of 100,000 parametric bootstrap replicates. N - sample size, g - observed number of genes (classes), f_0 - Number of genes missed, $f_1, f_2, \cdots, f_6$ - Estimated frequencies of frequencies.

	N	g	f_0	f_1
g	0.6363	1.0000	-1.0000	-0.9626
f_0	-0.6363	-1.0000	1.0000	0.9626
f_1	-0.7949	-0.9626	0.9626	1.0000
f_2	-0.8546	-0.9259	0.9259	0.9898
f_3	-0.8724	-0.9055	0.9055	0.9814
f_4	-0.8786	-0.8907	0.8907	0.9743
f_5	-0.8840	-0.8765	0.8765	0.9667
f_6	-0.8913	-0.8617	0.8617	0.9578

From Table C-4 we can see that the sample size, N, is negatively correlated with the values of $f_0, f_1, \cdots, f_6$; however as we have seen this relation is in general parabolic and not a straight line (plot shown only for N versus f_0, Figure C-6). On the other hand, the lineal correlation between f_0 and $f_1, f_2, \cdots, f_6$ is high and decreases with the subindex r, been maximum between f_0 and f_1, 0.9626, and going down to 0.8617 for f_0 with f_6. However, the relation between the values of the f's, even when high, is not well fitted by a straight line, as can be seen in figures C-8 and C-9 for f_0 *versus* f_1.

From figures C-8 and C-9 we can see that the best straight lineal fit between f_1 and f_0 (green dotted line, Adjusted R-squared: 0.9266) has an intercept of 155 and a slope smaller than one (0.59), and in any case gives a poor fit due to the clear curvature present in the relation between f_1 and f_0. Also the strong relation between the sample size, N, and f_0, f_1 is made apparent by the coloring of the plotted points

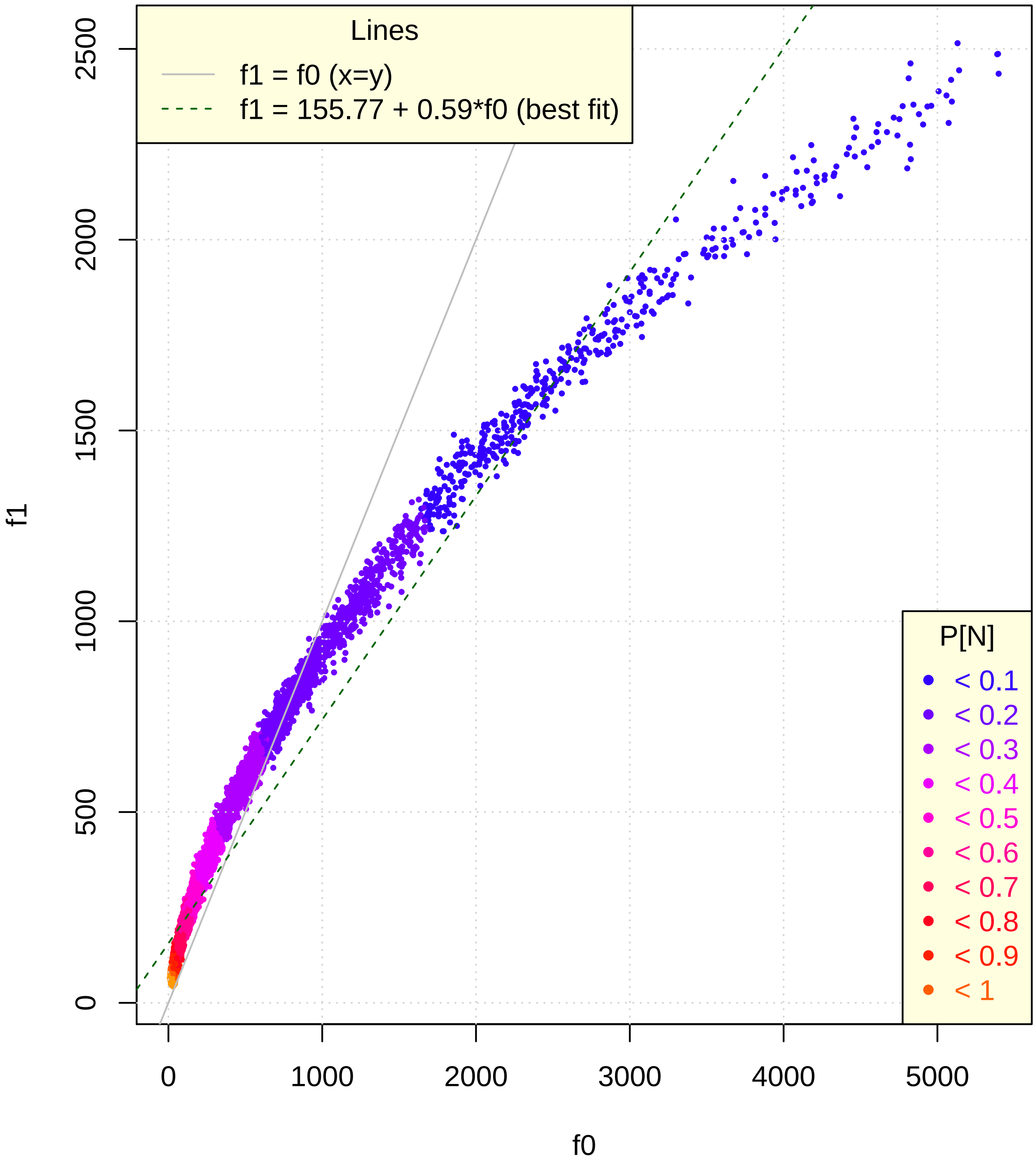

FIGURE C-8. Scatterplot of the values of f_0 and f_1 obtained in the simulated data. Color of the points signals the size of the sample, N, as proportion of the complete sample size of around 160 millions. Gray line signals the points $x = y$. All rank for f_0 and f_1 is shown. Figure C-9 shows an amplification of the low-left region.

dependent on sample size, expressed in the bottom-right legend as proportion of the original sample, $P[N]$. Small sample sizes give large values of f_1 and f_0 (bluish colors) and also the opposite is true; large sample sizes give small values of f_1 and f_0 (reddish colors). This can be better appreciated in Figure C-9. Even when less curvature is present in the relations between the pairs $(f_r, f_j); r < j, r = 1, 2, \cdots, 5; j = 2, 3, \cdots, 6$ (plots not shown), these relations are not well explained by a straight line, and make clear the need of a non-straight relation between f_1 and $f_2, f_3, \cdots$, as the one postulated in the Chao1 estimator as well as in our estimators (equation C-4).

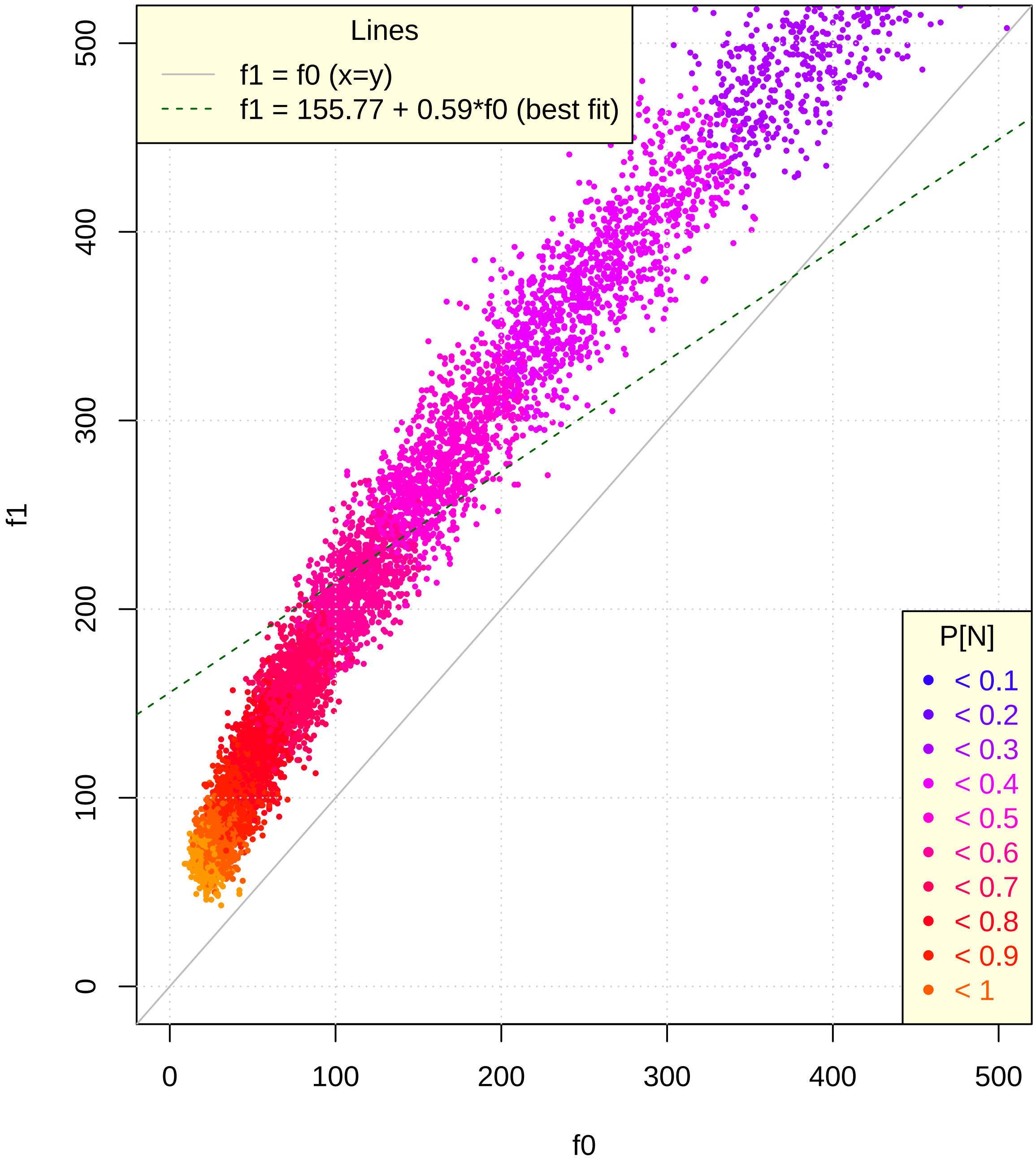

Figure C-9. Subplot from Figure C-8. Only values between 0 and 500 for f_0 and f_1 are shown.

From the lineal correlations between the unobservable number of missing genes, f_0, and the observable frequencies $f_1, f_2, \cdots$, partially presented in Table C-4, we can understand how the first terms, $f_r, r = 1, 2, \cdots, 6$ include almost all relevant information about f_0 and thus using higher order frequencies ($r > 6$) will be insignificant for accurate and precise estimation.

C.2.2. *Comparing h_6 with the Chao1 and Medial.* The high dependence of f_0 with $f_1, f_2, \cdots$, discovered by Turing and Good [6] was exploited by Chao [1] and others [12] to construct non-parametric estimators of f_0, using only singletons and doubletons (f_1 and f_2). Here we propose to use the extra information *via* the harmonic mean to improve the estimation with our estimator h_6. Thus we use the performance

of the Chao1 ($f_1^2/2f_2$) and Medial (f_1^2/f_2) estimators as baselines to compare with the performance of h_6 in the set of $B = 100,000$ samples from the complete dataset previously obtained.

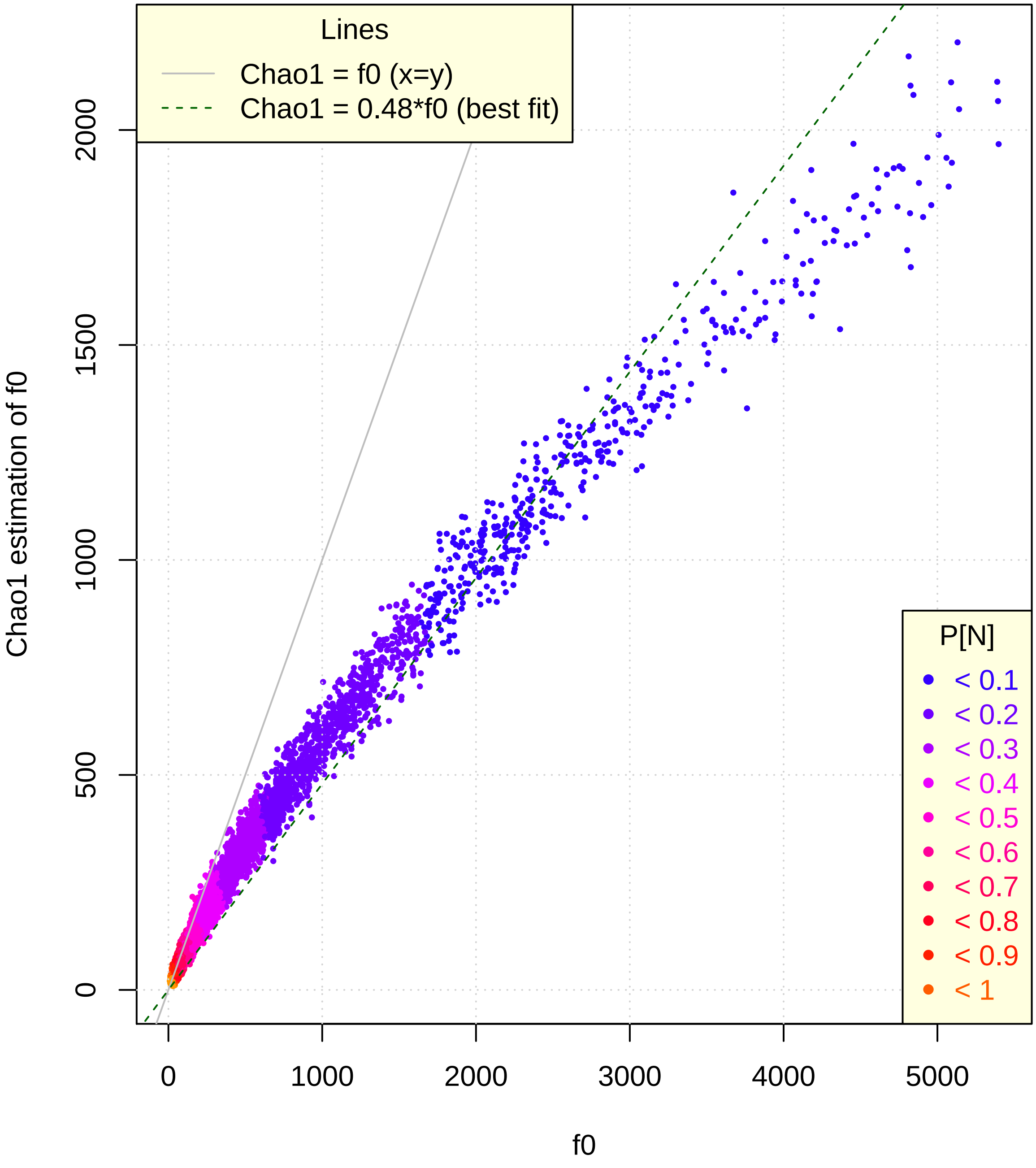

FIGURE C-10. Scatterplot of f_0 *versus* $\hat{f}_0$ estimated with Chao1 estimator.

Figures C-10 and C-11 show scatterplots for the values of f_0 (X-axis) and $\hat{f}_0 = f_1^2/2f_2$, the Chao1 estimations of f_0 (Y-axis). From Figure C-10 we can see that Chao1 underestimate f_0 almost everywhere; in fact, this happens in 85,470 of the 100,000 bootstrap replicates, that is in around 86% of the points. The reverse, i.e., Chao1 been equal or larger than f_0 happens in 14,530 (15%) of the bootstrap replicated with large sample sizes (Median of $P[N]$ equal to 0.7857, corresponding to $N = 126$ millions approximately). The standard error of the estimator, measured by C-5, over all samples has a value of 386 for the Chao1 estimator.

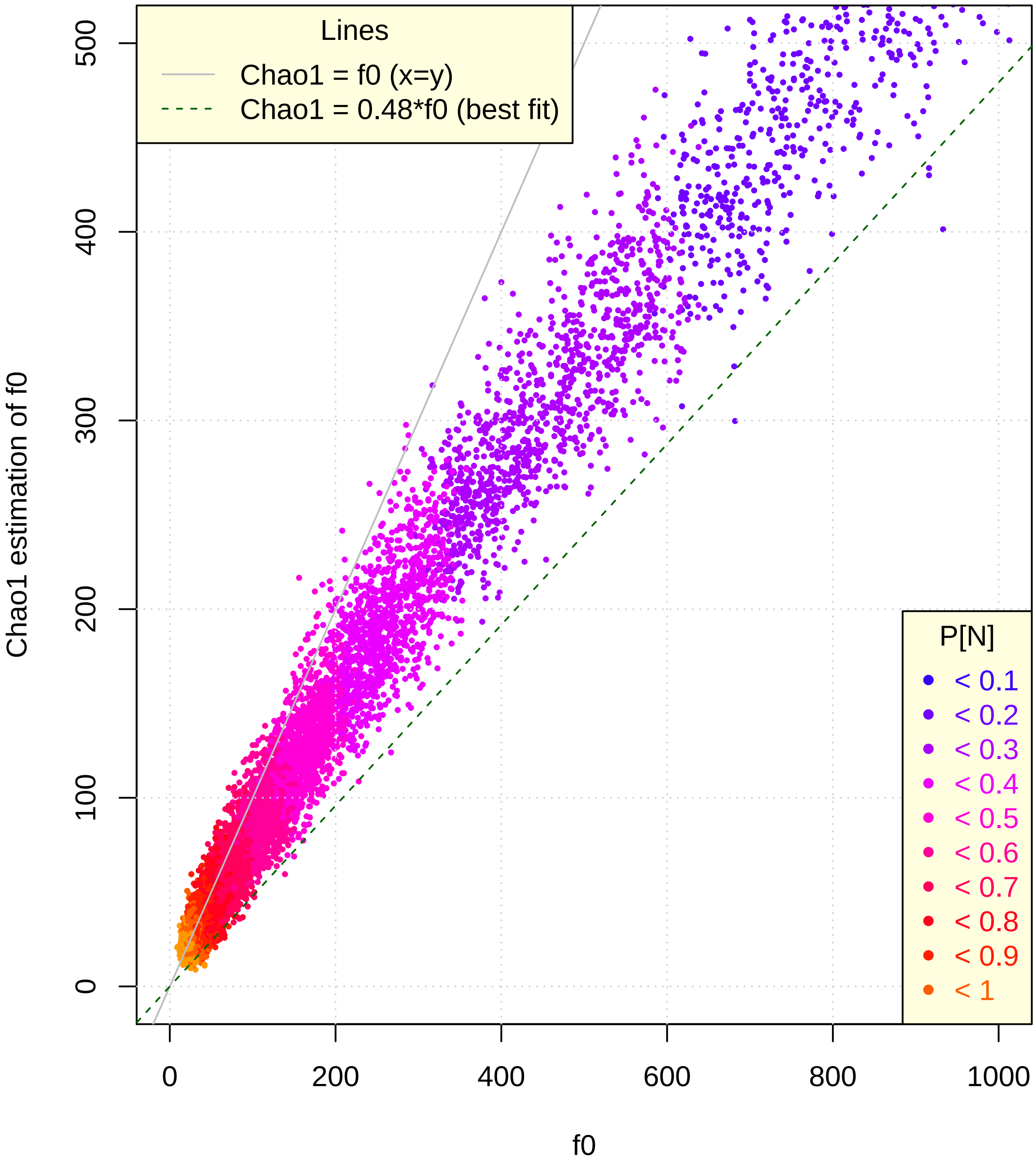

FIGURE C-11. Subplot from Figure C-10. Scatterplot of f_0 *versus* $\hat{f}_0$ estimated with Chao1 estimator.

The fact that Chao1 underestimates f_0 was already noticed by Chao herself, who mention that this estimator is only a lower bound estimator [1]. By fitting a liner model without intercept, $f_0 = \beta \hat{f}_0 + \epsilon$, we obtain an estimate for the slope $\hat{\beta} \approx 1/2$ and a residual standard error equal to 141, much smaller than the value of $se(\hat{f}_0) = 386$. The line corresponding to the fitted model $f_0 \approx \hat{f}_0/2$ for the case of the Chao1 estimator is represented by a green dotted line in Figures C-10 and C-11. The fact that the Chao1 estimator underestimate was corrected by Xu and collaborators [12] that proposed the Medial estimator as twice the value of Chao1. This estimator corresponds, almost exactly, with the one obtained by fitting the linear model and shown in Figures C-10 and C-11.

Table C-5 presents the formulae, standard errors, coefficient of determination r^2 between $\{f_0, \hat{f}_0\}$, and minimum (Min), median, mean and maximum (Max.) for the errors $(f_0 - \hat{f}_0)$ in the set of $B = 100,000$ pseudo samples with varying sample size from the complete dataset. The constants in the estimators formulae where estimated from the corresponding lineal models rounding to one decimal place, and some vary slightly (± 0.05) from the ones previously estimated, from independent samples assuming the Multinomial distribution (column $\hat{u}$ in Table C-2).

Table C-5. Numerical comparison of estimators for f_0 evaluated in B =100,000 bootstrap replicates of the complete dataset using random sample sizes, N. The formulas, estimated standard error, $se(\hat{f}_0)$ (eq. B-2), as well as the standard error compared with the standard error of Chao1, $P[se(Ch1)]$, the estimated coefficient of determination between the estimated values and f_0, r^2 and statistics for the errors $\hat{f}_0 - f_0$ (minimum, median, mean and maximum) are presented for various estimators.

Estimator	Formula	Standard Error		r^2	Error $(\hat{f}_0 - f_0)$			
	$\hat{f}_0 =$	$se(\hat{f}_0)$	$P[se(Ch1)]$	$(\hat{f}_0, f_0)$	Min.	Median	Mean	Max.
Chao1	$f_1^2/2f_2$	386	1.0000	0.9671	-3,717	-22	-6	68
Medial	f_1^2/f_2	141	0.3653	0.9671	-1,861	56	63	519
a_3	$9f_1^2/10\bar{f}_3$	118	0.3058	0.9755	-1,633	41	52	425
a_4	$8f_1^2/10\bar{f}_4$	104	0.2693	0.9804	-1,540	27	34	375
a_5	$7f_1^2/10\bar{f}_5$	104	0.2693	0.9837	-1,656	13	10	282
a_6	$7f_1^2/10\bar{f}_6$	84	0.2183	0.9859	-1,335	20	33	418
g_3	$9f_1^2/10\hat{f}_3$	115	0.2996	0.9765	-1,573	42	54	440
g_4	$8f_1^2/10\hat{f}_4$	98	0.2537	0.9822	-1,399	29	39	389
g_5	$7f_1^2/10\hat{f}_5$	90	0.2322	0.9859	-1,461	15	17	322
g_6	$7f_1^2/10\hat{f}_6$	83	0.2164	0.9883	-1,092	23	43	568
h_3	$9f_1^2/10\tilde{f}_r$	114	0.2945	0.9775	-1,512	42	56	460
h_4	$8f_1^2/10\tilde{f}_r$	94	0.2437	0.9837	-1,271	30	44	435
h_5	$7f_1^2/10\tilde{f}_r$	79	0.2058	0.9876	-1,270	16	25	436
h_6	$6f_1^2/10\tilde{f}_r$	84	0.2172	0.9900	-1,534	3	-3	264

As in Table C-2 (using Multinomial distribution), in Table C-5 (assuming Poisson), we can see that the values of the constants ($\hat{u}$ in Table C-2, and expressed as fractions in Table C-5), generally decrease as function of r for arithmetic (a's), geometric (g's) and harmonic (h's) estimators, indicating that in general, when we take more frequencies into account (larger r) f_0 is a smaller fraction of f_1^2.

We can also see in Table C-5 that the value of r^2 (that do not depends on the constant factor) increases as function of the number of frequencies included, $r = 3, 4, 5, 6$, and this happens for the three types of estimators, i.e., as seen before, the use of the extra information implies a gain from around $r^2 \approx 0.97$ for Chao1 up to $r^2 \approx 0.99$ for a_6, g_6, h_6. Also -and more important, the estimated standard error of the estimator (column $se(\hat{f}_0)$) substantially decreases as function of r for the three types of estimators; the proportion of the standard error with reference to the Chao1 estimator (column $P[se(Ch1)]$) reach around 0.21 for $r = 6$ in a_r, g_r, h_r, demonstrating that the inclusion of f_3, f_4, f_5 and f_6 in the formulae give a better estimator; i.e., one with around 21% of the original standard error of the Chao1 estimator.

None of the estimators proposed (including Chao1 and Medial) could be called strictly unbiased, because the expectation of these functions diverges given that the probabilities of the denominator being zero are not null; i.e., under any sensible distribution it can be shown that $P[f_r = 0] > 0$ for $r = 2, 3, \cdots ,$. However, from the practical point of view and for large enough values of sample size, N, the relative *bias* of the estimators can be measured by the statistics for their estimated errors (minimum, median, mean and maximum). Using this criterium we can see that the estimator h_6 is the less biassed, having a

median and mean error equal to a 3 and -3. This is remarkable, if we remember that these estimates are obtained for a wide variety of sample sizes, going from around 1 up to 160 millions of tags.

Summarizing the results presented in Table C-5, and reinforcing the facts seen in Table C-2, we conclude that the best estimator for f_0 is the harmonic estimator h_6.

Figures C-12 and C-13 present scatter plots of f_0 *versus* h_6 -the estimate using the harmonic estimator of order 6, while Figure C-14 presents a plot of the errors, $h_6 - f_0$ *versus* N (sample size). From Figure C-12 we can see that the estimator h_6 is very well behaved for values of $f_0 \leq 1,000$ (see also Figure C-13) and for values of $f_0 > 2,000$ it begins to underestimate f_0. This underestimation is larger for smaller sample sizes, as can be noted in Figure C-14. However, this underestimation is present mainly in small sample sizes, and overall 98% of all errors have an absolute value of less than 200.

It can be rightly argued that an estimator obtained by a heuristic procedure using a single (complete) dataset could be of small value if the good behavior of the estimator is not preserved in general for every RNA-seq dataset, or an least for a comprehensive set of these. Thus we will see if the better behavior of h_6 is confirmed in other independent and almost complete datasets.

C.3. **Validating h_6 as estimator of f_0.** RNA-seq experiments are performed under a large variety of mixtures of cells, tissues, organs, and under very diverse conditions. For this, it is necessary to test the performance of the estimators in various frameworks. To test the performance of the proposed estimators, mainly the harmonic estimator of order 6, h_6, which was the best in the complete mouse dataset, we will try them into three independent and almost complete datasets.

The selected datasets, MPSS data from humans tissues [7], and accessions E-GEOD-38298[2] (*Candida albicans*) and E-GEOD-46953[3] (*Mus musculus*) were selected by the criterion of small f_1, which indicates that the number of missing genes, f_0, is *small* and thus it could be considered that $f_0 \approx 0$; i.e., these datasets were considered to be *complete* (containing all genes that are in fact being expressed).

The procedure to test the estimators was as previously described for the complete sample, except that the constant for the estimator h_6 was not estimated *de novo*, but was kept to the proposed value of 6/10. In summary,

(1) A sample, $N = (N_1, N_2, \cdots, N_{100,000})$, of 100,000 uniform pseudo-random numbers was generated from the interval $(m, 1)$ where $m\mathbf{N} \approx 1,000,000$ (i.e., the minimum expected sample size will be around 1 million) and the maximum $1 \times \mathbf{N}$ was equal to the sample size of the original sample denoted as $\mathbf{N}$.

(2) For each one of the 100,000 values of expected sample size, N_i, a vector of G random numbers with independent Poisson distributions of parameters $\lambda = N_i p$ was generated, where $p = \mathbf{y}/\mathbf{N}$ is the vector of estimated relative frequencies in the original dataset of length G. Each one of the bootstrap replicates, say $y_i^b; i = 1, 2, \cdots, 100,000$ is a *parametric bootstrap* replicate, for which we know the true value of f_0; i.e., $f_{0i} = G - g_i^b$, where f_{0i} is the number of zeroes observed in the bootstrap replicate i and it is equal to the length of the original vector G minus the number of non-zero values observed in the bootstrap replicate, say g_i^b.

(3) For each bootstrap replicate y_i^b the estimated values of $f_0, f_1, f_2, \cdots, f_6$ were obtained and the estimates of f_0 were calculated with the formulas presented before.

(4) The performance of the estimators was measured by their standard error (se) and their median error. Results are plotted and graphically presented.

[2]E-GEOD-38298 - RNA sequencing revealed novel actors of the acquisition of drug resistance in *Candida albicans.*

[3]E-GEOD-46953 - Default DNA Methylation is Preceded by Broad, Low-Level Transcription in Fetal Male Germ Cells and Is Inversely Patterned by Dynamic H3K4 Methylation (RNA-Seq)

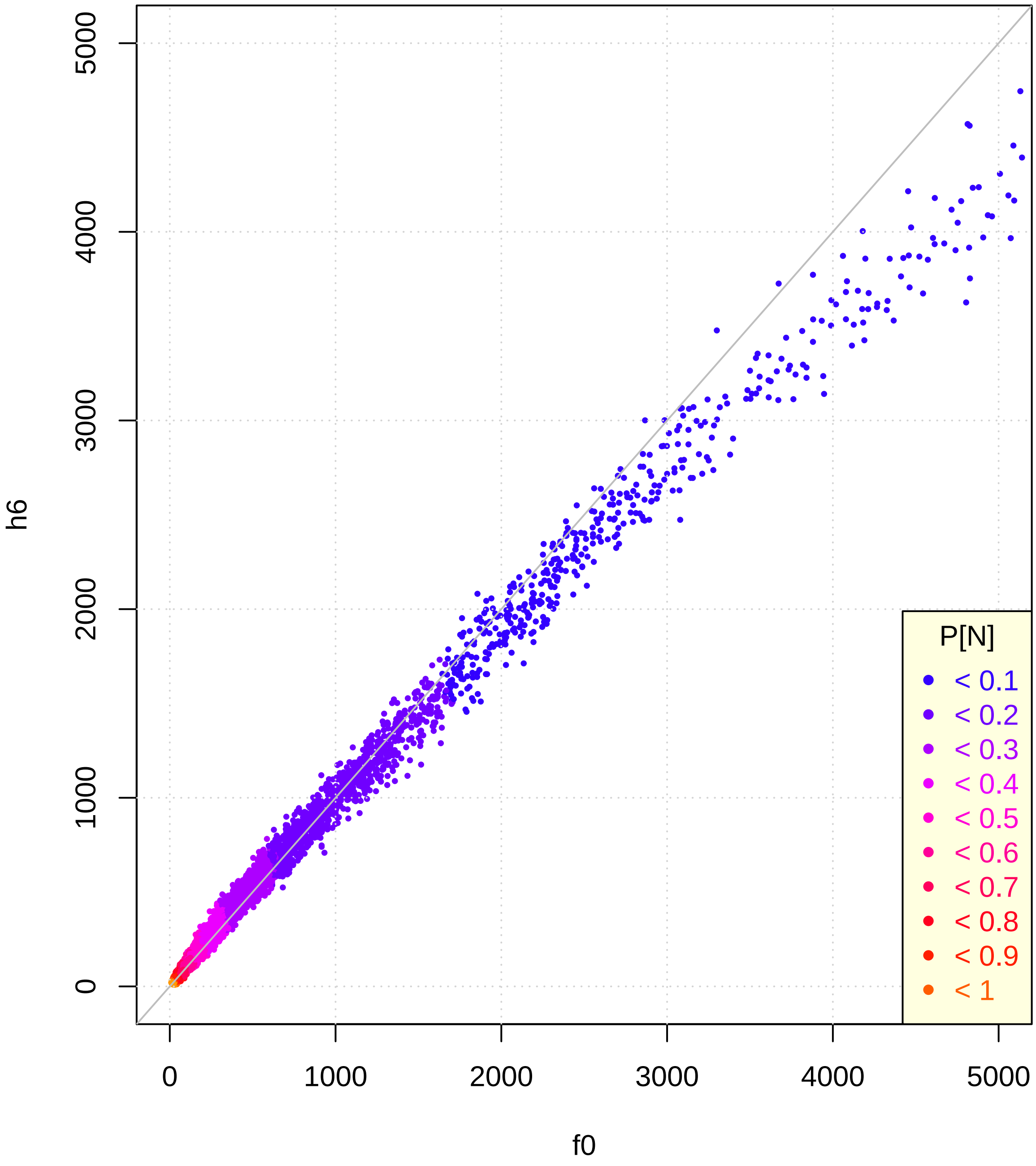

FIGURE C-12. Scatterplot of f_0 *versus* $\hat{f}_0 = h_6$, i.e., the values of f_0 estimated with the harmonic estimator 6.

C.3.1. *Testing Chao1, Medial, a_6, g_6 and h_6 in the human MPSS dataset.* The first dataset to test the estimator was presented in [7] and has been re-analyzed previously by us with regard to transcriptome diversity and specialization [9]. This dataset consist in data for $G = 22,935$ human genes expressed in 32 distinct tissues. As for the mouse dataset, we collapsed all tissues into a single vector which has $\mathbf{N} = 31,411,949$ mapped tags. This dataset is *almost complete* because it has values of $f_1 = 9, f_2 = 27$, which give a very small estimate of $f_0 = 3$ missing genes by employing Chao1 estimator. Note that the sample size of this human dataset is less than 20% the size of the sample size in the complete mouse dataset used in the previous sections, 31.4 millions in the human *versus* 160.5 millions in the mouse

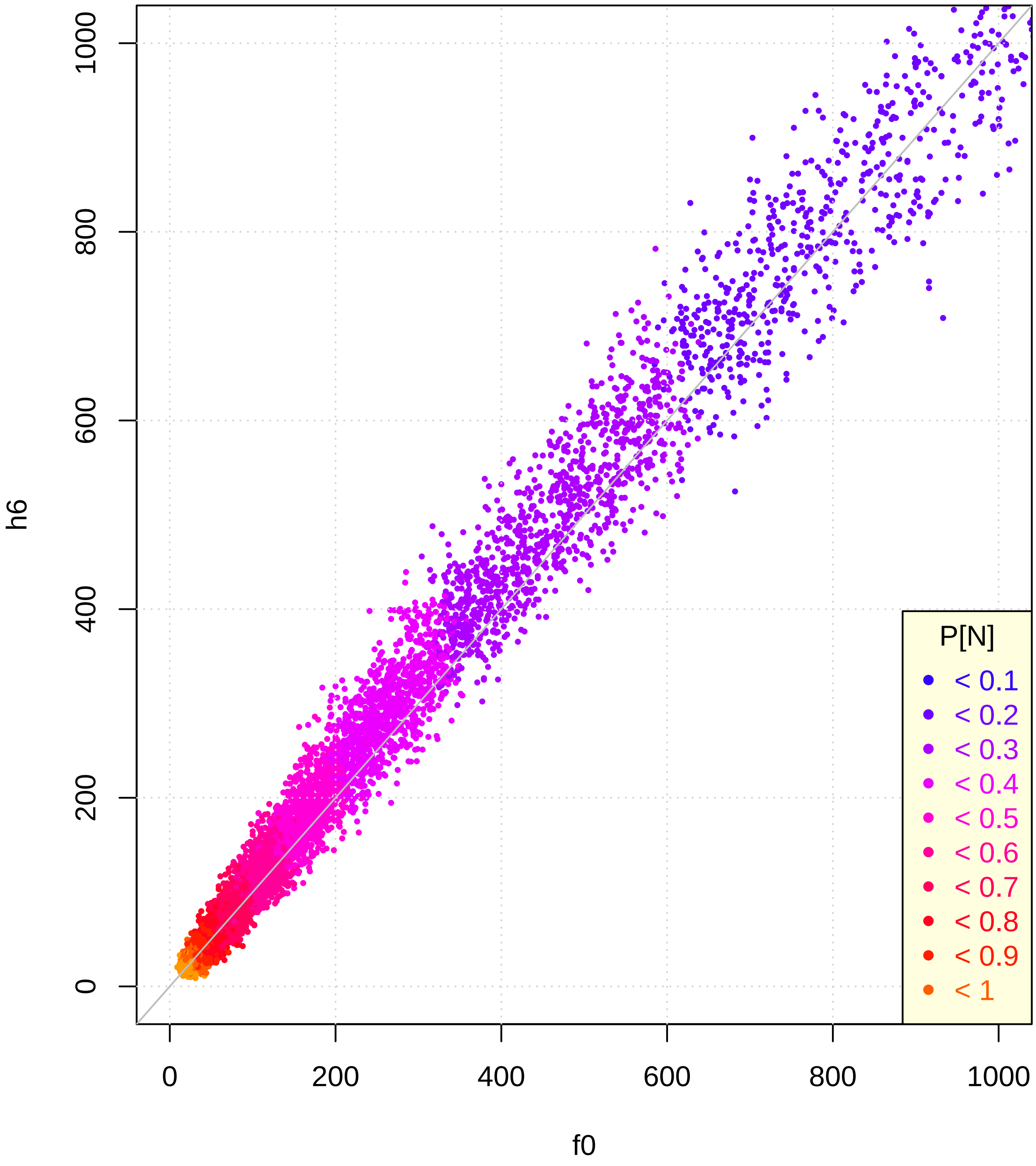

FIGURE C-13. Subplot from Figure C-12. Scatterplot of f_0 *versus* $\hat{f}_0 = h_6$, i.e., the values of f_0 estimated with the harmonic estimator 6.

dataset that was estimated as fully complete. This smaller sample size in the human dataset could cause more noise in the estimation of f_0.

As for the mouse dataset, we obtained 100,000 parametric bootstrap replicates of the data, varying the sample size in a uniform random way between proportions 0.001 and 1 of the original sample size. Table C-6 present the main statistics for the human dataset and the 100,000 parametric bootstrap replicates obtained from it using variable sample sizes, N, covering in a uniform way the rank $\mathbf{N}(0.001, 1)$.

From Table C-6 we can appreciate the fact that the analyzed human dataset is *almost* complete; the observed values of $f_1 = 9, f_2 = 27$ give a very small estimate of $\hat{f}_0 = 3$ (row *Full*). Comparing this row

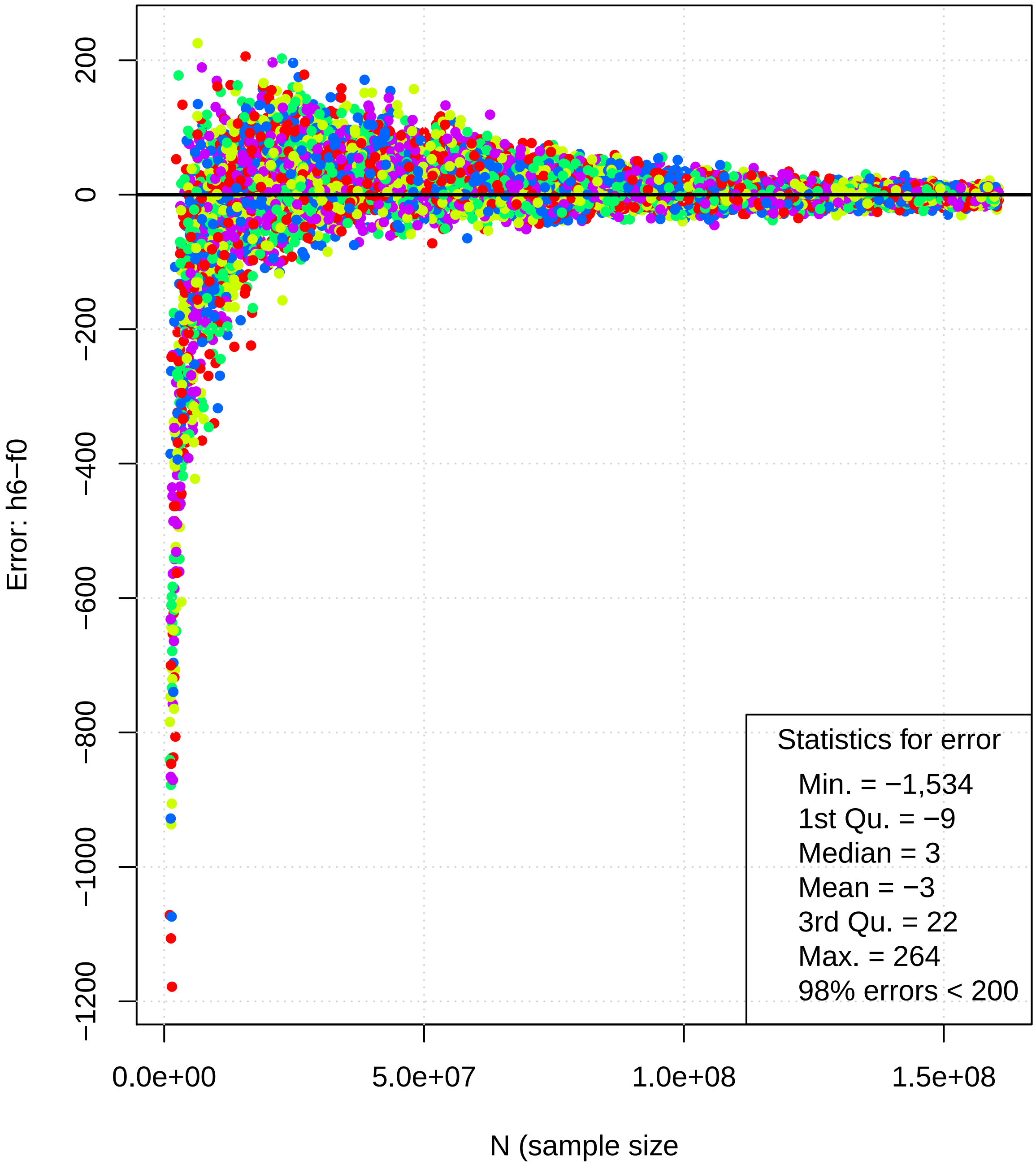

FIGURE C-14. Estimated error $h_6 - f_0$ *versus* N (sample size). Only 10% of the 100,000 points are plotted, but the statistics for error are for all 100,000 points. Points color at random.

with the corresponding *Full* row of Table C-3 (the complete mouse dataset), we can see that the values of the total number of detected genes (22,935 for humans, Table C-6 and 23,332 for mouse, Table C-3) are of comparable size; however, assuming that humans have about the same number of expressed genes than mouse, the human dataset will be lacking 23,332 - 22,935 = 397 genes. This comparison is not completely fair because the number and nature of the tissues analyzed is different for the two datasets and, the sample size is far larger (160.5 millions) for mouse compared with the one for humans (31.4 millions), i.e, the sample for mouse is more than 5 times larger. Also, the MPSS methodology employed in the human dataset is less powerful than the one employed in the mouse dataset.

TABLE C-6. Statistics for the human dataset (row *Full*) and a set of 100,000 parametric bootstrap replicates. N - sample size, g - observed number of genes (classes), $n0$ - Number of genes missed (not observed), $f_1, f_2, \cdots, f_6$ - Frequencies of frequencies.

Statistic	N	g	f_0	f_1	f_2	f_3	f_4	f_5	f_6
Full	31,411,949	22,935	0 (**3**)	9	27	15	23	439	346
Minimum	31,437	6,849	3	20	53	109	161	211	174
Mean	15,732,265	22,305	630	633	616	589	555	514	472
Median	15,751,785	22,836	99	265	423	516	537	516	478
Maximum	31,421,472	22,932	16,086	3,603	1,920	1,315	1,006	832	689
S	9,042,262	1,490	1,490	802	515	344	230	154	105

Comparing the tendencies of the statistics for the frequencies f_0 to f_6 presented for the human dataset (Table C-6) with the ones in the mouse dataset (Table C-3), we can observe that in general there is a larger change from f_i to $f_{i+1}; i = 0, 1, \cdots 5$ in the human compared with the mouse datasets; for example, the medians of the replicates in the human dataset are $f_0 = 99, f_1 = 265, f_2 = 423, f_3 = 516, f_4 = 537, f_5 = 516, f_6 = 478$, while for the mouse dataset the medians of the replicates are $f_0 = 107, f_1 = 207, f_2 = 245, f_3 = 243, f_4 = 226, f_5 = 208, f_6 = 192$. While the median number of missing genes per sample (f_0) in the two datasets are roughly the same (99 *vs.* 107), the occurrence of *rare* genes (f_1 to f_6) appears to be much larger in the human than in the mouse dataset. This could be partially explained by the huge differences in sample sizes (see above), as well as by the differences in tissues employed as well as sequencing platforms; however, the fact remains that the two datasets are fundamentally distinct in statistical properties (distributions), and thus to test the estimators obtained with the mouse dataset in the human dataset will give a solid proof of the suitability of the estimators under very different conditions, i.e., for very different RNA-seq experiments.

In Table C-7 we can observe the correlations between N, g, f_0 and f_1 with the frequencies $f_2, \cdots, f_6$ in the human replicates and compare them with the ones obtained for the same quantities in the case of the complete mouse dataset (Table C-4).

TABLE C-7. Estimated Pearson's linear correlation coefficients (r) in the set of 100,000 parametric bootstrap replicates of the human dataset. N - sample size, g - observed number of genes (classes), $n0$ - Number of genes missed (not observed), $f_1, f_2, \cdots, f_6$ - Frequencies of frequencies.

	N	g	f_0	f_1
g	0.5756	1.0000	-1.0000	-0.8684
f_0	-0.5756	-1.0000	1.0000	0.8684
f_1	-0.8303	-0.8684	0.8684	1.0000
f_2	-0.9404	-0.7151	0.7151	0.9541
f_3	-0.9818	-0.6056	0.6056	0.8823
f_4	-0.9897	-0.5253	0.5253	0.8172
f_5	-0.9808	-0.4603	0.4603	0.7626
f_6	-0.9602	-0.3978	0.3978	0.7113

Comparing Table C-7 with Table C-4 we can observe than even when the tendencies are the same; i.e., there is a negative relation between the sample size, N, and the frequencies $f_0, \cdots, f_6$ the value of $r(N, f_0)$ is larger in the mouse dataset (-0.6363) than in the human (-0.5756). Even when in both cases the correlation between sample size and frequencies of rare genes $r(N, f_i); i = 1, 2, \cdots, 6$ increases with i, these values are larger in the human (Table C-7; from -0.8303 to -0.9602) than in the mouse (Table C-4; from -0.7949 to -0.8913), i.e., the frequencies of rare genes are more sensitive to sample size in the human dataset (with smaller sample size) than in the mouse dataset. On the other hand, the correlations between f_0 and $f_1, f_2, \cdots, f_6$ are always larger (in absolute value) in the mouse compared

with the human dataset; for example, $r(f_0, f_6) = 0.8617$ in the mouse (Table C-4), and this value is less than half $r(f_0, f_6) = 0.3978$ in the human dataset (Table C-7). This indicates that there is much more lineal information about missing genes (f_0) in the case of the mouse dataset than in the case of the human. We confirm our previous conclusion about the large statistical differences in the distribution on both datasets.

Table C-8 presents the results for some of the estimators in the human dataset. Other estimators were also tried, but their statistical performance was inferior and thus they are not shown here.

TABLE C-8. Numerical comparison of different estimators for f_0 evaluated in B =100,000 bootstrap replicates of the human (almost) complete dataset using different sample sizes. The formulas, estimated standard error, $se(\hat{f}_0)$, as well as the standard error compared with the standard error of Chao1, $P[se(Ch1)]$, the estimated coefficient of determination between the estimated values and f_0, r^2 and statistics for the errors $\hat{f}_0 - f_0$ (minimum, median, mean and maximum) are presented for various estimators.

Estimator	Formula	Standard Error		r^2	Error $(\hat{f}_0 - f_0)$			
	$\hat{f}_0 =$	$se(\hat{f}_0)$	$P[se(Ch1)]$	$(\hat{f}_0, f_0)$	Min.	Median	Mean	Max.
Chao1	$f_1^2/2f_2$	929	1.0000	0.8990	-12,596	-17	-257	79
Medial	f_1^2/f_2	498	0.5360	0.8990	-9,209	58	116	1367
a_6	$7f_1^2/10X_6$	426	0.4589	0.9366	-5,242	2	148	2071
g_6	$7f_1^2/10G_6$	474	0.5105	0.9479	-3,396	3	196	2536
h_6	$6f_1^2/10H_6$	358	0.3859	0.9565	-3,661	-4	118	1988

From Table C-8 we can see that the statistical performance of the estimators a_6, g_6, h_6 is much better than the one for the Chao1 and Medial estimators in the case of the human dataset, confirming the results presented in Table C-5 for the complete mouse dataset. All three estimators, a_6, g_6, h_6, using the information from small frequencies f_1 to f_6, are better than the Chao1 and Medial estimators that only use f_1 and f_2. This can be seen in their smaller standard error ($se(\hat{f}_0)$), larger value of correlation with f_0 (r^2; determination coefficient) and smaller estimated bias, measured by the median of the error. As before (see Table C-4) the best estimator result to be the harmonic estimator of degree 6, h_6; even when it presents an estimated mean error of 118, larger than the corresponding value for the Medial estimator, 116, the median of the error is smaller, -4, than for the Medial estimator.

Figures C-15 and C-16 present the scatterplot for the values of f_0 *versus* the values estimated by Chao1, Medial and the h_6 estimators in 10,000 bootstrap replicates, selected at random from the 100,000 performed for the human dataset.

From Figures C-15 and C-16 we can see how the h_6 estimator has an overall better fit than either, the Chao1 or Medial estimators. Even if for some values of f_0, for example between $2,500 < f_0 < 5,000$ the Medial estimator appears to be less biased (and thus have small standard error in that region) the general pattern for small values of f_0 (see Figure C-16) as well as for large values $f_0 > 7,500$ (Figure C-15), h_6 is closer in average to the true value of f_0 -the grey line in both figures.

An interesting and unexpected result is that in this case (human data) h_6 changes its curvature from concave (around $f_0 < 7,500$) to convex (above $f_0 \approx 7,500$), automatically correcting the estimated value and giving values of $\hat{f}_0 = h_6$ much closer to the true value of f_0 than the Chao1 or Medial estimators, which on this rank diverge from the value of f_0, tending asymptotically to a fixed value of around 3,500 for Chao1 and 7,500 for the Medial estimator, inducing a strong bias in the estimation.

From Figure C-16 we can see that for large sample sizes (and thus small values of $\hat{f}_0 \leq 500$) the h_6 estimator is better than either, the Chao1 and Medial estimators.

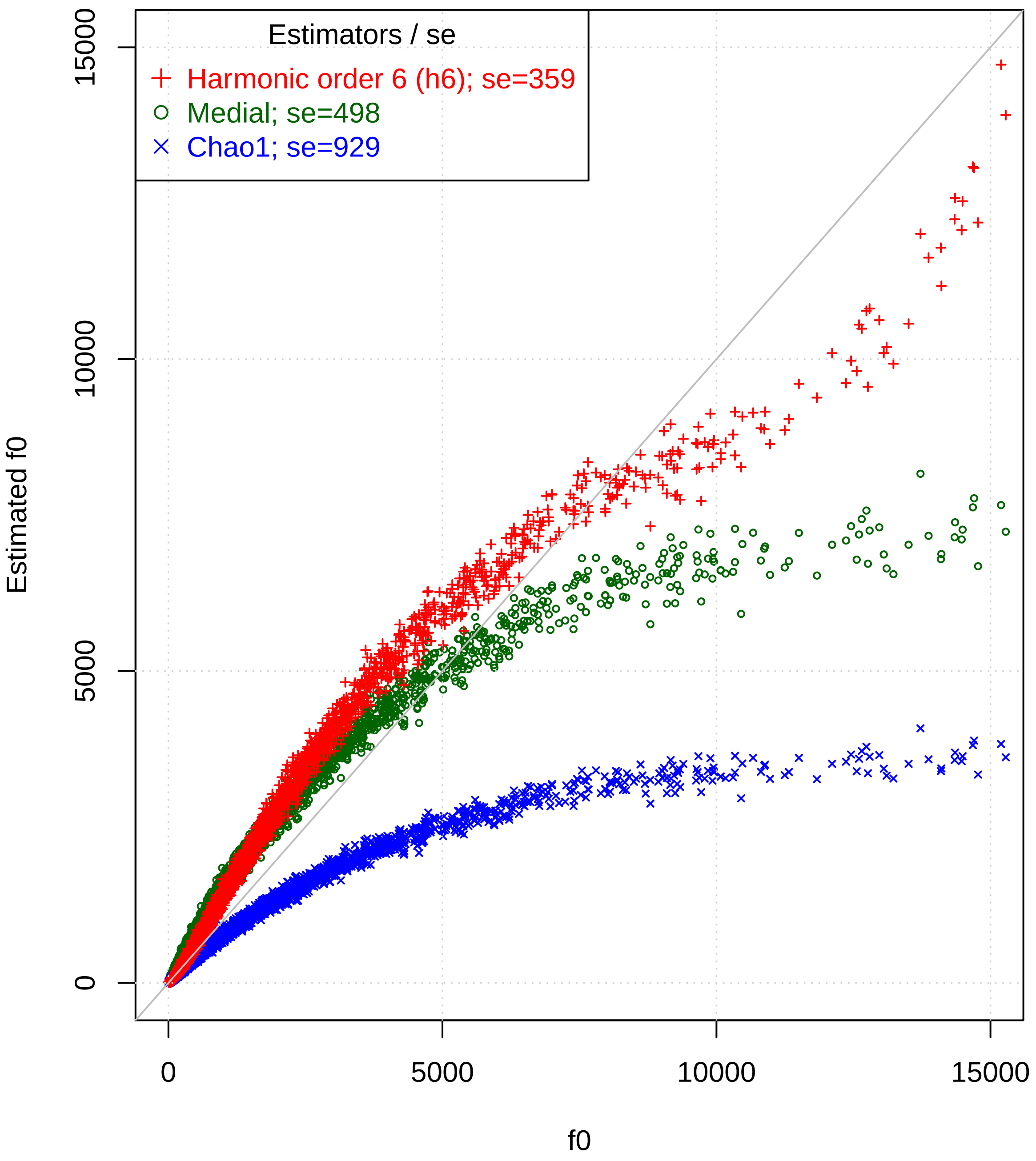

FIGURE C-15. Scatterplot of f_0 versus its estimated values in the human dataset employing three estimators: h_6, Medial and Chao1. Standard errors (se) for the three estimation in the whole dataset ($N = 100,000$), but only 10% of the 100,000 points are plotted.

In the next two sections we present the analysis of the performance of the estimators in other two almost complete datasets. Even when the degree of detail presented in the analyses is less than for the human MPSS dataset, all test were run and are available upon request.

C.3.2. *Testing Chao1, Medial and h_6 in the E-GEOD-38298 dataset (Candida albicans).* Dataset E-GEOD-38298 was downloaded from Array Express and detailed information about the experiment is available from `http://www.ebi.ac.uk/arrayexpress/experiments/E-GEOD-38298/`. This dataset consisted in counts for 4 sequenced libraries for a total of 6,205 genes. The matrix of counts was collapsed

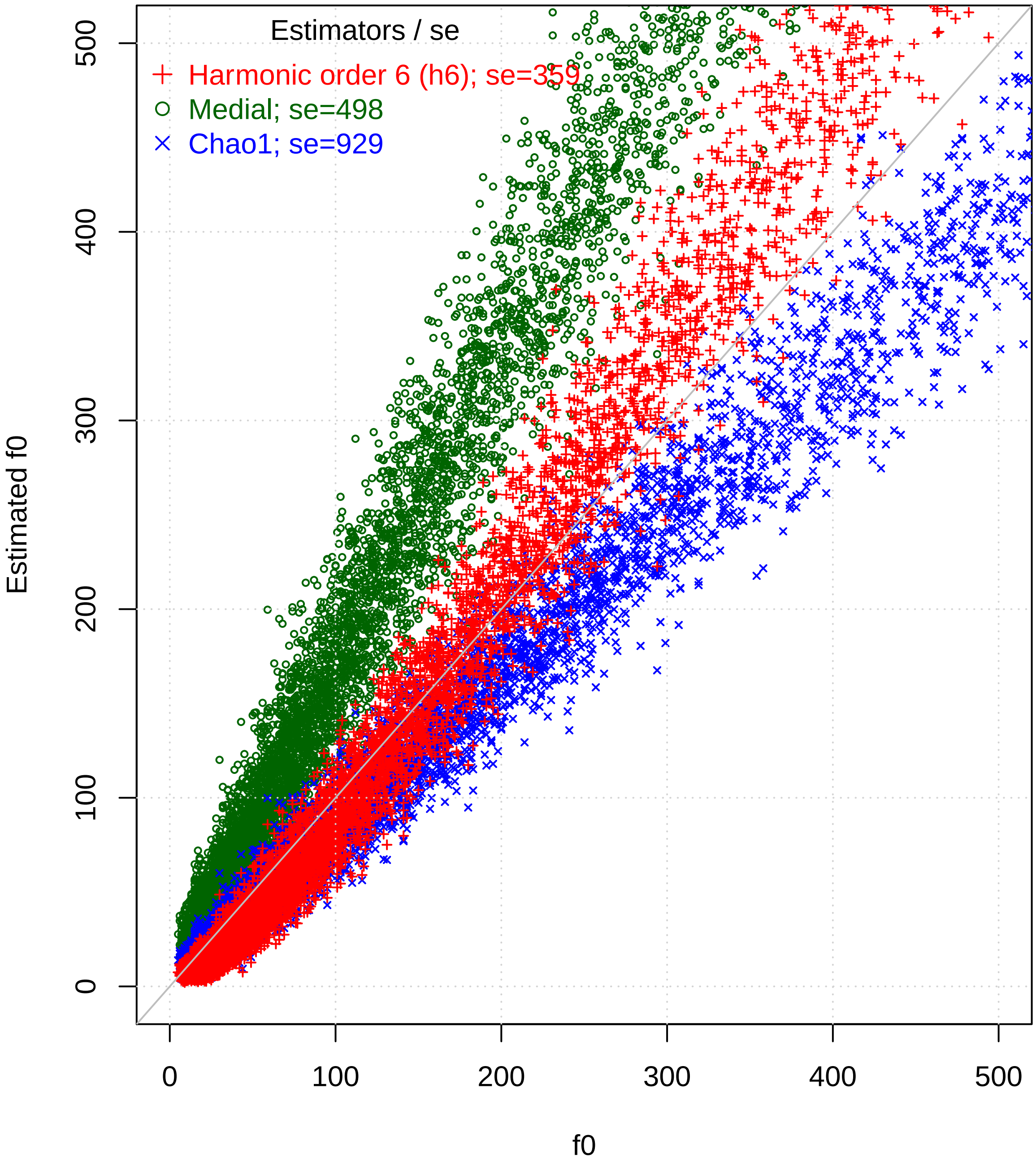

FIGURE C-16. Sub-plot of Figure C-15. Scatterplot of f_0 versus its estimated values in the human dataset employing three estimators: h_6, Medial and Chao1. Standard errors (se) for the three estimation in the whole dataset ($N = 100,000$), but only 10% of the 100,000 points are plotted.

by adding the counts of each gene in each library (adding rows). This resulted in a vector of total counts per gene with $G = 6,096$ expressed genes and a total sample size of $\mathbf{N} = 35,973,307$; almost 36 millions of gene tags in total. The values of $f_1, f_2, \cdots, f_6$ estimated in the original vector were 9, 46, 30, 2, 6 and 43 respectively giving estimates for f_0 equal to 1, 2 and 7 for the Chao1, Medial and h_6 estimators, thus this dataset can be considered *almost* complete (not many genes undetected). As before, a set of 100,000 parametric bootstrap replicates were obtained by the procedure detailed before, obtaining sample sizes from a minimum of $N = 1,008,209$ up to a maximum of $N = 35,981,426$ and a median of

$N = 18,585,829$, thus the interval $(0.02, 1)$ of proportions of sample sizes was uniformly covered. Figure C-17 presents a scatterplot of f_0 versus their estimated values using Chao1, Medial and h_6 estimators; as before, only 10% of the data obtained are plotted by clarity, but the statistics presented (standard error, *se* and median for errors) correspond to the complete set of 100,000 replicates. Errors were calculated as $f_0 - \hat{f}_0$.

In Figure C-17 we can observe that Chao1 estimator, as expected, give in general lower values than either the Medial or h_6 estimators, and this difference is larger for larger values of f_0, confirming the fact that the Chao1 estimator is a biased lower bound estimator. Also the median of the errors, $(f_0 - \hat{f}_0)$ is equal to 10 for Chao1, while it is -11 for the Medial and -3 for h_6, confirming the fact that the less biassed estimator of the three is the harmonic estimator of degree 6, h_6. In this case we can see that, even when the h_6 has the lower standard error ($se = 25$), the differences with the standard errors of the Chao1 and Medial (41 and 27, respectively) are not as large as in the case of the human dataset; still the best estimator is h_6 by the criteria of smaller bias and *se*.

C.3.3. *Testing Chao1, Medial and h_6 in the E-GEOD-46953 dataset (*Mus musculus*).* Dataset E-GEOD-46953 was downloaded from Array Express and detailed information about the experiment is available from `http://www.ebi.ac.uk/arrayexpress/experiments/E-GEOD-46953/`. This dataset consisted in counts for 4 sequenced libraries for a total of $G = 18,752$ genes with no-null expression, with a total sample size of $\mathbf{N} = 415,562,392$; more than 415.5 millions of tags. However, this dataset was not as 'complete' as the previous ones, having values of $f_1, f_2, \cdots, f_6$ in the original sample 40, 101, 29, 151, 18 and 40 respectively, giving estimates of f_0 equal to 8, 16 and 25 for the Chao1, Medial and h_6 estimators. As before, a set of 100,000 parametric bootstrap replicates were obtained by the procedure detailed before, obtaining sample sizes from a minimum of $N = 1,000,815$ up to a maximum of $N = 415,574,448$ and a median of $N = 208,201,682$, thus the interval $(0.002, 1)$ of proportion of sample sizes was uniformly covered. Figure C-18 presents a scatterplot of f_0 versus their estimated values using Chao1, Medial and h_6; even when only 10% of the data obtained are plotted by clarity, the statistics presented, standard error, *se* and median for errors, are for the complete set of 100,000 replicates. Errors were calculated as $f_0 - \hat{f}_0$.

In Figure C-18 we can observe that all three estimators severely underestimate the value of f_0 for $f_0 > 2,000$; these points correspond to relatively small sample sizes. The cases where $f_0 > 2,000$ correspond to 940 points, i.e., less than 1% of the 100,000 bootstrap replicates. These points are cases where the sample size goes from a minimum of $N = 1,001,000$ up to a maximum of $N = 4,941,000$ with a median of $N = 2,733,000$. This corroborates that for relatively small sample sizes, in this case for an interval (0.002, 0.012) in proportion of the original sample size of the data, $\mathbf{N} = 415,562,392$, none of the estimators tested is near unbiasedness. On the other hand, the best estimator of the three tested is, as in all previous cases, the harmonic estimator of degree 6, h_6. It has the smallest standard error, $se = 95$, compared with the values of standard errors for Medial and Chao1, 111 and 248 respectively. Also it is the less *biased*, if we employ as criterion the median of the errors.

C.4. **Conclusions about the validation of h_6.** The heuristic nature of the procedure to obtain and test the estimators proposed do not allows to assure that h_6 is the '*best*' of all possible estimators; indeed, we have seen that harmonic estimators of order $r > 6$ have a smaller standard error for the complete mouse sample. Also, the estimation of the numerical constant (u in equation B-1) is only approximate and thus the value of $u = 6/10$ set for h_6 here, is likely to be slightly different in other conditions, i.e., for other RNA-seq experiments. However, we have shown that h_6 performs better than the Chao1 or Medial estimators in three datasets, independent and different to the complete sample employed in the original design and testing of the estimators. This implies that even when cannot be assured that h_6

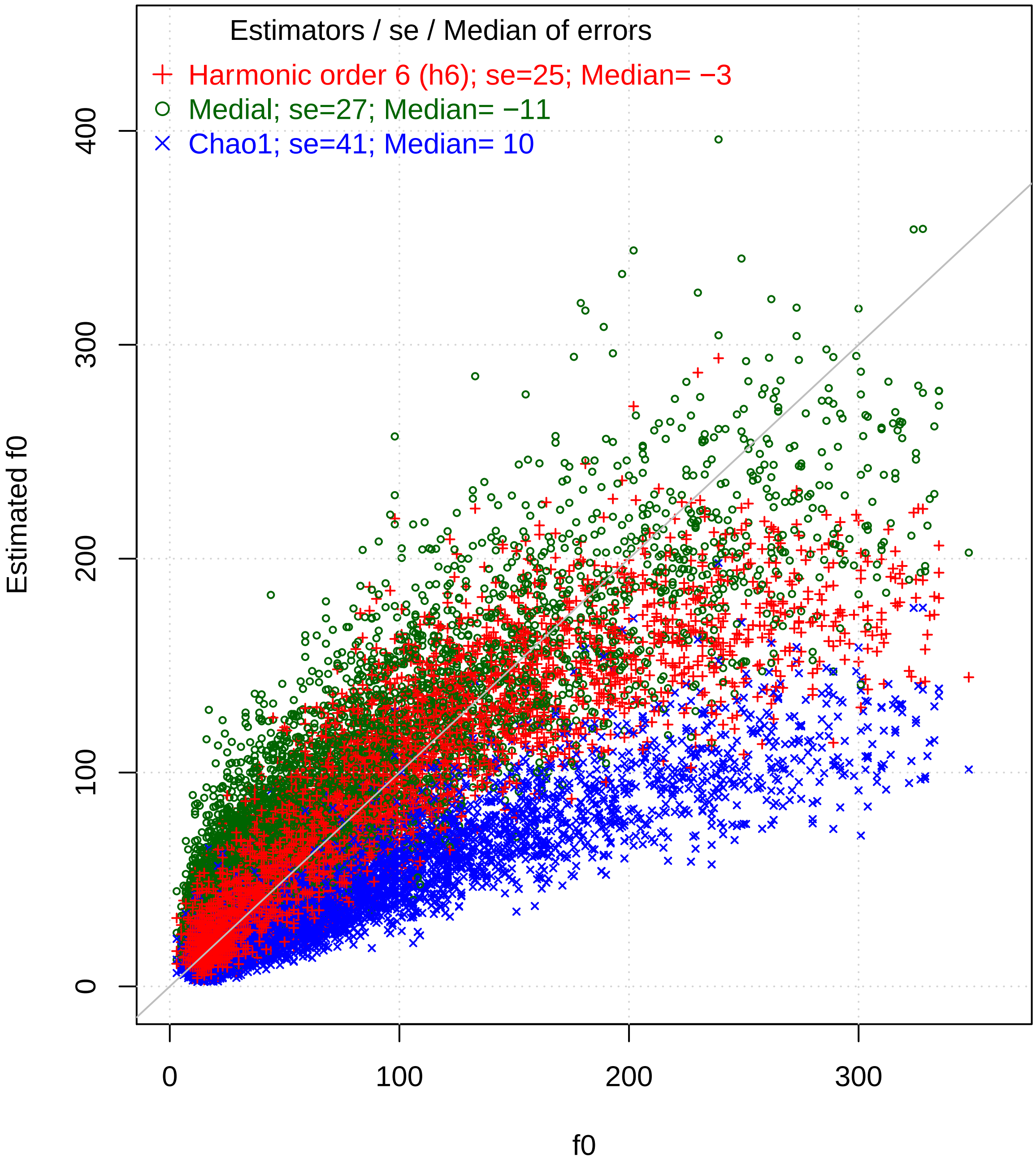

FIGURE C-17. Scatterplot of f_0 versus its estimated values in the E-GEOD-38298 dataset employing three estimators: h_6, Medial and Chao1. Standard errors (se) and median of errors for the three estimators are presented for the whole of the parametric bootstrap samples $(100, 000)$, but only 10% of the 100,000 points are plotted.

will be uniformly optimal for any RNA-seq experiment, it can be considered in practical terms a better option than other published options, mainly Chao1 and the Medial estimators[4].

Table C-9 summarizes the statistical performance of the Medial and h_6 estimators with reference to the Chao1 estimator in the four datasets studied, the complete dataset (row GSE1581) and the three

[4]We also tried a variety of functions, including some polynomial approximations and function of pairs f_1, $f_j; j = 1, 2, \cdots, f_6$. These are not presented because they gave consistently non better results than h_6.

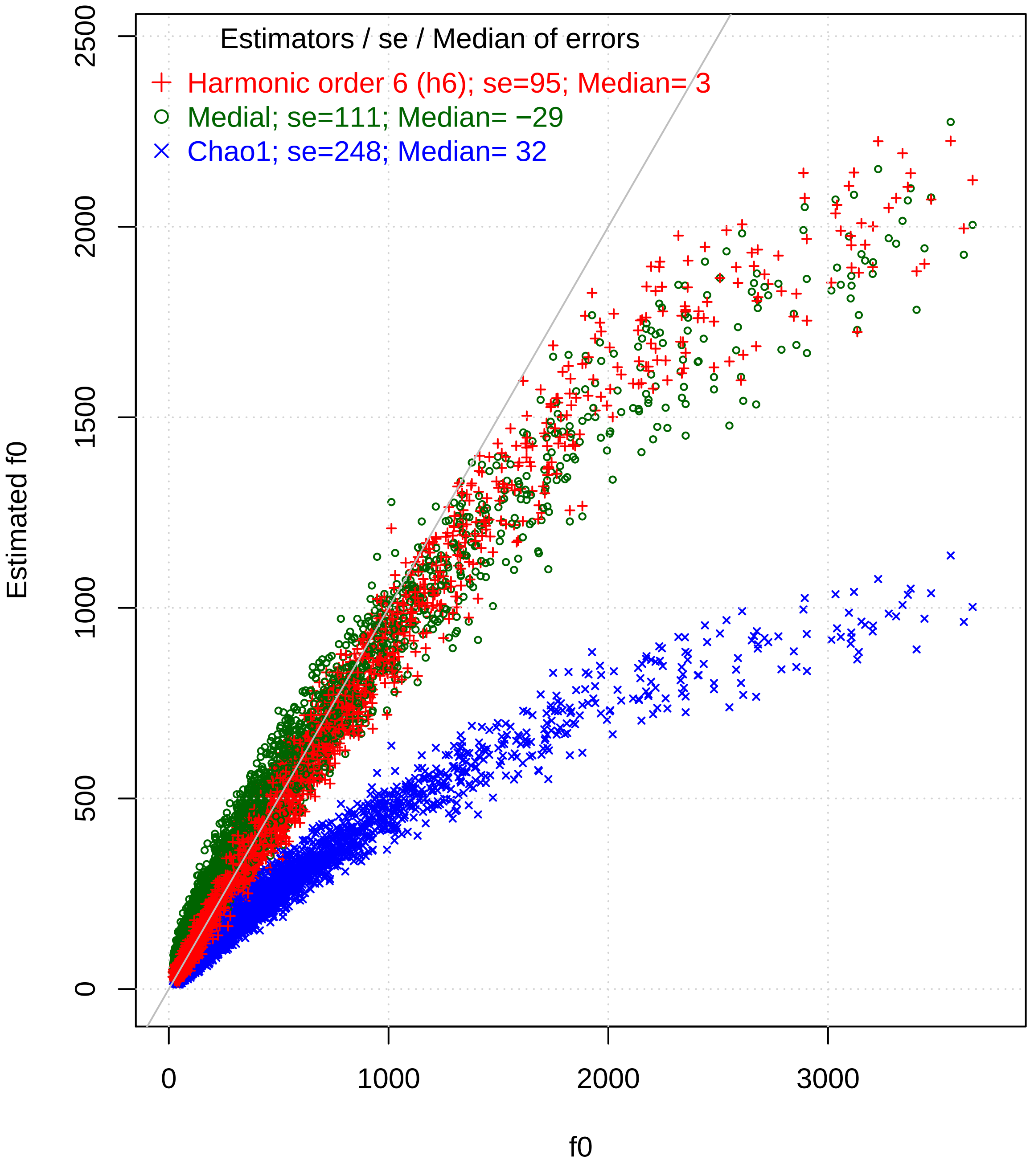

Figure C-18. Scatterplot of f_0 versus its estimated values in the E-GEOD-38298 dataset employing three estimators: h_6, Medial and Chao1. Standard errors (se) and median of errors for the three estimators are presented for the whole of the parametric bootstrap samples $(100,000)$, but only 10% of the 100,000 points are plotted.

datasets used for validation (Human MPSS, E-GEOD-38298 and E-GEOD-46953); see also Table 2 in main text.

From Table C-9 we can see that h_6 is superior as estimator of f_0 than Chao1 and Medial estimators. The estimator h_6 gives a percentage of standard error relative to the Chao1 estimator that goes from 22% in the GSE1581 up to 61% in the E-GEOD-38298 datasets. For the Medial estimator the corresponding interval goes from 37 to 66%; worst than h_6 in all cases. Note that these measures of performance are, in

Table C-9. Sample sizes in the dataset (**N**) and measures of performance for the Medial and h_6 estimators of f_0 with reference to the Chao1 estimator in the four datasets analyzed. $\%se$ - Percentage of standard error with reference to the one obtained with Chao1; %bias - Percentage of median absolute error, $\mid median(f_0 - \hat{f}_0) \mid$, with reference to the one obtained with Chao1.

		Estimator			
		Medial		h_6	
Dataset	**N**	$\%se$	%bias	$\%se$	%bias
GSE1581	160,552,086	37	255	22	14
Human (MPSS)	31,411,949	54	341	39	24
E-GEOD-38298	35,973,307	66	110	61	30
E-GEOD-46953	415,562,392	45	91	38	9

all cases, taken into account sample sizes that go from around one million -an empirically found value, up to the complete sample size of the original data (**N** in Table C-9). Using the absolute difference of the median error values (% bias in Table C-9) we can also see that h_6 has a much better central tendency than Chao1 or the Medial estimators; under some conditions, Medial appears to have a larger *bias* than even the Chao1 estimator, which is know to underestimate f_0 (values of % bias above 100% for Medial in Table C-9).

In conclusion, we can say that the behavior of the h_6 estimator is consistently better than the previously published Chao1 and Medial estimators in samples from very different contexts -human, fungus and mouse datasets, which present large differences in sample sizes as well as in the distributions of rare genes.

D. Approximate confidence intervals for f_0

As usual, having a punctual estimation of the quantity of interest, f_0 in our case, is not sufficient; we want to have a confidence interval estimation for this parameter. Given that the Chao, Medial and h_6 estimators are non-parametric, we could see if the bootstrap procedure, or some of its variants, could give satisfactory confidence intervals in this case. Unfortunately we will see that the basic plug-in principle for bootstrap [5] is not fulfilled, given the fact that incomplete samples (samples where not all expressed genes are observed) do not contain information about all relevant parameters, i.e., there is not a sufficient statistic in the incomplete sample. This in turn implies that there are not satisfactory MLE for the quantity of interest.

D.1. **Properties of bootstrap estimates of f_r.** We have used parametric bootstrap resampling on complete (or *almost* complete) samples to design and test f_0 estimators. This is justified by the fact that in complete samples we can assume that the total number of genes expressed, G, has been observed in the dataset and then the observed proportions $\hat{p}_i = y_i/\mathbf{N}$ are MLE of the corresponding parameters $\mathbf{p} = (p_1, p_2, \cdots, p_G)$, i.e., the frequencies of expression of each one of the genes. However, in incomplete samples, where we can suspect that there are missing genes by the fact that we observe a large quantity of singletons, say $f_1 \gg 0$, we do not have good (MLE) estimators for the relative frequencies of expression. As noted by Good [6] this is due to the fact that the MLE for the frequencies of all genes not *observed* in the sample are identically zero; i.e., MLE assume that the sample is complete or that there are not other genes undetected in the sample. The opposite, where $f_0 > 0$, is precisely the case of interest.

Here we briefly present some of the characteristics of the estimators for **p** that in turn affect the estimation of the values of $f_r|N$ and limit the information that can be gained about these by the bootstrap procedure.

D.1.1. *The estimators of* $\mathbf{p}$ *are biased in incomplete samples.* Order the vector of true parameters, $\mathbf{p}$, from the smallest to the largest, and index this vector by i, say

$$p_1 \leq p_2 \leq \cdots \leq p_s \leq p_{s+1} \leq p_{s+2} \leq \cdots \leq p_G$$

i.e., we have $p_i \leq p_{i+1}$ and of course $\sum_i p_i = 1$. Now assume that in a particular incomplete sample, $\mathbf{y}$, only genes corresponding to the subindexes $r > s$ are observed, thus we have $f_0 = s$ genes missing, and we have a vector of realized observations $\mathbf{y} = (y_r, y_{r+1}, \cdots, y_{G-s})$, that indexed by natural numbers j corresponds to $\mathbf{y} = (y_1, y_2, \cdots, y_g)$ with all values $y_j > 0$ and $g = G - s$, the estimated number of genes, i.e., the number of genes observed in the sample. The total sample size is $\sum_j y_j = N$ and our estimators (not MLE!) for the values of the frequencies of the observed genes is the vector $\hat{\mathbf{p}}$ with elements $\hat{p}_j = y_j/N$ and length g where $\sum_j \hat{p}_j = 1$.

It is clear in the previous example that, if we think that our estimators $\hat{p}_j$ are MLE, we will be 'underestimating' all p_i, $i \leq s$ by considering them as identically zero. On the other hand, all values of $p_{s+1}, p_{s+2}, \cdots, p_G$ estimated respectively by $\hat{p}_1, \hat{p}_2, \cdots, \hat{p}_g$ are likely to be overestimated (at least as group), because

$$\sum_{j=1}^{j=g} \hat{p}_j = 1 > \sum_{i=s+1}^{i=G} p_i = 1 - \sum_{i=1}^{i=s} p_i$$

From this we conclude that the estimation of the frequencies of expression from incomplete samples are <u>intrinsically</u> biased.

D.1.2. *Bootstrap estimates of* f_r. Now we will study some characteristics of the bootstrap estimates of the frequencies of frequencies, using the ordered vector of parameters $\mathbf{p}$ introduced above. Remember that $f_0, f_1, f_2, \cdots$ are not parameters of the distribution, but just attributes of it, that change depending on the sample size N, as functions of $\mathbf{p}$. Denote by $\mathbf{y}_b^*$ a parametric bootstrap replicate of our realized incomplete sample $\mathbf{y}$ (defined in the previous section). To obtain $\mathbf{y}_b^*$ we use as parameters the sample size N and the estimated relative frequencies $\hat{\mathbf{p}}$ and produce a realization of the Multinomial distribution using a pseudorandom generation routine, as the function `rmultinom` of R. From the realized $\mathbf{y}_b^*$ we calculate the observed value of 'missing' genes, $\hat{f}_{0b}^*$, i.e., the number of realized values of the vector $\mathbf{y}_b^*$ that are equal to zero. This procedure can be repeated a large number of times, say B, and the bootstrap procedure will give a 'good' estimation of the true value of the parameter, f_0, if and only if the mean, say

$$\bar{f}_0^* = \frac{1}{B} \sum_{b=1}^{b=B} \hat{f}_{0b}^*$$

converges to the true value of f_0 that in this case is known to be s, the number of missing genes in the *original* sample. Assume that

$$\lim_{B \to \infty} \bar{f}_0^* = s$$

However, this is absurd, because there is no information whatsoever in the realized sample $\mathbf{y}$ about the unseen number of missing genes, s. In fact, the probability that one bootstrap replicate from the realized sample $\mathbf{y}$ takes a particular value, say $P[\hat{f}_{0b}^* = x]$, depends only on the incomplete set of parameters $\{N, \hat{\mathbf{p}}\}$, where the length $\hat{\mathbf{p}}$ is $g < G$, while the probability of obtaining $P[\hat{f}_0 = x]$ in a *new* and arbitrary sample of the same size N from the original population depends on the complete set of parameters $\{N, \mathbf{p}\}$, where the length of $\mathbf{p}$ is G. We have proven by *reductio ad absurdum* that $\bar{f}_0^*$ is not a good estimator of f_0, in incomplete samples, and this can be generalized for $f_1, f_2, \cdots$ using the same argument, i.e., the incompleteness of the parameter space of a specific (incomplete) sample with reference to the true parameter space.

In tables C-3, for the complete accession GSE1581, and C-6, for the almost complete human MPSS dataset, we have seen the behavior of the bootstrap estimators for f_r in incomplete samples, when varying the sample size uniformly in a large rank. Here we will empirically investigate the statistical behavior of the bootstrap estimators for f_r, say f_r^*, in a complete dataset (the mouse accession GSE1581), when

the sample size employed in the resampling procedure, N, is the same than in the complete dataset, $\mathbf{N}$. In the case of this accession we have $G = 23,332$ and $\mathbf{N} = 160,552,086$. We obtained a large number of bootstrap replicates, $B = 100,000$, using $N = 160,552,086$ and the vector of 'true' probabilities $\mathbf{p} = \mathbf{y}/\mathbf{N}$; i.e., we assume that the observed frequencies in the complete dataset are the true frequencies of expression of the genes. This exercise has the aim of demonstrating how, even when 'large' samples of the true distribution using exhaustive bootstrap, the bootstrap estimators f_r^* are 'biased' in the sense that they do not reflect the true values of f_r in the 'population', i.e., in the complete dataset. Table D-10 shows the statistics for the bootstrap replicates while Figure D-19 shows box plots for the distributions of the bootstrap replicates.

Table D-10. Values of f_r^* (rows) in the complete sample ('true' column, accession GSE1581), statistics (Min., Median, Mean, Max. and S) and Bootstrap percentile interval at 95% confidence level (LL - Lower limit, UL - Upper limit) for $B = 100,000$ bootstrap replicates using as parameters $N = 160,552,086 = \mathbf{N}$ and the vector of 'true' probabilities $\mathbf{p} = \mathbf{y}/\mathbf{N}$.

		Bootstrap samples					CI (95%)	
f^*	'true'	Min.	Median	Mean	Max.	S	LL	UL
f_0^*	0	5	21	22	41	4	13	31
f_1^*	0	31	62	62	92	7	48	76
f_2^*	74	63	100	100	139	9	83	118
f_3^*	139	81	125	125	169	10	105	145
f_4^*	169	90	135	136	190	11	115	157
f_5^*	146	92	136	136	182	11	115	158
f_6^*	148	91	132	132	178	11	111	153
f_7^*	143	83	125	125	172	11	105	146
f_8^*	107	71	118	118	163	10	98	138
f_9^*	117	71	111	111	161	10	92	131
f_{10}^*	101	65	105	105	149	10	87	125

In Table D-10 we can see that the minimum value of f_0 in the $B = 100,000$ replicates is 5, thus none of this large number of re-samples is 'complete' by including all $G = 23,332$ expressed genes. As we have seen (section 2.2) the probability to obtain more than zero missing genes, $P[f_0 > 0|N, \mathbf{p}]$, is very large (≈ 1), even with large samples; in fact, in Supporting file 'S2 Excel' the sample size calculated for this data to have a probability of around 0.05 of no missing genes is of more than 587 millions, around 4 times more than the sample size employed in the bootstrap replicates (see also Table B-1 here). Also Table D-10 shows that, in general, the mean of the bootstrap estimates of f_r is far from the 'true' value of the complete data; for example, for f_0, f_1, f_2 and f_4 the 95% confidence intervals, obtained by the bootstrap percentile method, do not include the true value of the corresponding f_r. Also, in Figure D-19 we can see that, except for f_9 and f_{10}, the true value of f_r (red asterisk) is not within the interquartile part of the distributions. In summary, we conclude that the bootstrap procedure do not efficiently recover information from the frequency of frequencies, and this can be attributed to the incompleteness of the samples. Given that the bias or lack of precision of the bootstrap procedure is due mainly to an incomplete set of parameters in the original sample, this cannot be corrected by the usual procedures of 'bias correction' [5, 4] for bootstrap confidence intervals.

D.2. **Unsuitability of Bootstrap Percentile Confidence Intervals for h_6.** As seen in the previous section, the estimates of frequencies $\hat{f}_r^*$ do not asymptotically reflect the value of the corresponding attribute, f_r, in the sample; i.e., they are intrinsically biased due to the incompleteness of the parameters in the sample. In turn, this bias cause a bias in the estimators for f_0, as the Chao1, Medial, h_6 or, in general, in any estimator function of f_r; $r = 1, 2, \cdots,$. As mentioned, this bias in the $\hat{f}_r^*$, and consequently in the estimators, cannot be corrected employing bias correction procedures (data not shown).

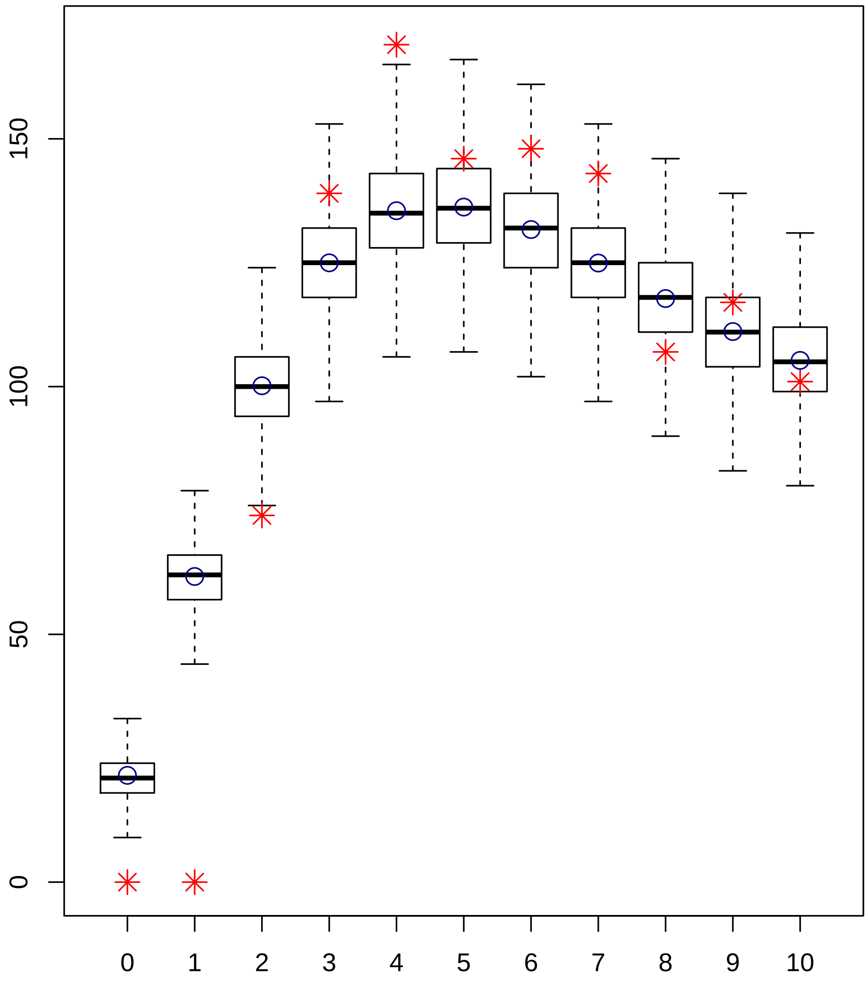

FIGURE D-19. Distributions (as box plots) for the values of $f_r^*; r = 0, 1, \cdots, 10$ (X-axis) in $B = 100,000$ bootstrap samples from the complete accession GSE1581. True values (in the original data) are presented as red asterisks, while the mean of the bootstrap replicates is shown as a blue circle.

We will see that a consequence of the bias of the estimators is that the usual bootstrap percentile confidence intervals in many practical cases do not contains the value of f_0 estimated from the sample, say $\hat{f}_0$, and this happens for the Chao1, Medial and h_6 estimators.

Table D-11 shows the statistics for the estimation of f_0 *via* the h_6 estimator in the 'total' data for each one the public accessions studied (see Analysis in main text).

In Table D-11 we can see the estimates of f_0 using the h_6 estimator in the sample, say, $\hat{h}_6$, as well as the bias, standard error and the 95% limits for the bootstrap percentile intervals (LL = Lower Limit and UL = Upper limit) plus a qualitative variable 'Out?' indicating if the confidence interval included or not the value $\hat{h}_6$ estimated from the sample, i.e., this variable is '*no*' if LL$\leq \hat{h}_6 \leq$UL, i.e., when the value of $\hat{h}_6$ is *not* out of the interval and '*yes*' when the value $\hat{h}_6$ is out of the interval.

These statistics were calculated from $B = 1,000$ bootstrap replicates, $\hat{h}_6^*$, by the Poisson distribution (and thus include variability in sample size within the rank of that distribution), using in each case as parameters for the rate of expression of each gene the values $\lambda_i = y_i;\ i = 1, 2, \cdots g$, where g is the number

Table D-11. Statistics for estimated value of missing genes *via* h_6 and $B = 1,000$ bootstrap replicates of the estimation in 'total' samples of all public accessions employed in the study. See text.

					CI		
Accession	$\hat{h}_6$	bias	$se(\hat{h}_6)$	%CV	LL	UL	Out?
GSE1581	0	19	4	-	11	28	yes
HumanMPSS	1	4	2	200	3	10	yes
E-GEOD-38298	7	8	7	100	6	31	no
Sunflower	23	-11	5	22	4	26	no
E-GEOD-48862	38	82	17	45	90	157	yes
E-GEOD-38435	39	14	8	21	37	70	no
E-GEOD-33016	47	-14	8	17	20	50	no
E-GEOD-43667	53	125	22	42	138	224	yes
E-MTAB-1178	137	-54	12	9	62	109	yes
E-GEOD-51091	289	-180	21	7	72	153	yes
E-GEOD-34914	291	-96	26	9	147	247	yes
E-GEOD-27971	383	-161	24	6	175	271	yes
E-GEOD-44171	521	-236	31	6	230	351	yes
E-GEOD-48147	1,250	-720	45	4	446	626	yes
E-GEOD-38435	1,576	-700	52	3	779	987	yes
E-GEOD-45474	1,613	-956	50	3	561	756	yes
E-GEOD-37544	1,680	-1,015	57	3	555	785	yes
E-GEOD-53024	1,760	-955	58	3	700	918	yes
E-GEOD-56890	1,761	-1,071	55	3	591	806	yes
E-GEOD-42960	1,881	-1,172	57	3	603	826	yes
E-MTAB-651	2,050	-1,216	62	3	720	957	yes
E-GEOD-29992	2,429	-1,496	67	3	813	1,072	yes
E-GEOD-29162	2,752	-1,634	66	2	991	1,253	yes
E-GEOD-16868	3,421	-2,175	76	2	1,101	1,403	yes
E-GEOD-16789	4,270	-2598	84	2	1,501	1,839	yes
E-GEOD-29163	4,295	-2,630	85	2	1,504	1,838	yes
GSE54123	4,786	-1,521	111	2	2,055	3,492	yes
E-GEOD-29134	5,403	-3351	94	2	1,865	2,233	yes
E-GEOD-33793	5,588	-2,804	111	2	2,555	2,990	yes
E-GEOD-44384	7,131	-4,584	117	2	2,329	2,788	yes

of genes observed and thus the expected sample size was $E[N] = \sum_i \lambda_i = N$, the original sample size. The bias was estimated as

$$\bar{h}_6^* - \hat{h}_6$$

where $\bar{h}_6^*$ is the mean of the bootstrap replicates of the estimator; the estimate of the standard error, $se(\hat{h}_6)$, was estimated as the standard deviation of the bootstrap replicates; a relative Coefficient of Variation in percentage, %CV, is calculated as $100 \times se(\hat{h}_6)/\hat{h}_6$ and the confidence intervals, 'CI', where calculated by the 0.025 and 0.975 sampling quantiles of the bootstrap replicates ('LL' and 'UL', respectively) [5].

From Table D-11, which is sorted in ascending order of $\hat{h}_6$, we can see that the estimated bias is large, relative to the corresponding $se(\hat{h}_6)$ and in the majority of the cases negative (24/30, 80%), indicating that usually $\bar{h}_6^* < \hat{h}_6$, a fact that can be explained by the incompleteness of the sample; i.e., we are usually *underestimating* the number of missing genes when using the bootstrap procedure. Note that the cases where the bias is positive, $\bar{h}_6^* > \hat{h}_6$, occur when the estimated number of missing genes is small; concordantly, there is a decrease in the %CV as the estimated number of missing genes increases.

The most remarkable feature of Table D-11 is the high proportion the cases where the 95% percentile confidence interval do *not* includes the value estimated from the original sample, i.e., the 26/30 (87%) of the cases where the column '*Out?*' is equal to '*yes*'. This failure of the bootstrap percentile interval to include the value estimated from the sample is also a consequence of the incompleteness of the sample; i.e., the bootstrap replicates do not have information about the frequencies of the genes missing. The only 4 cases (4/30, 13%) in which the CI includes the value estimated from the sample occur in relatively complete accessions, with a low estimated number of missing genes, $\hat{h}_6 \leq 47$.

From the evidence obtained from the analyses in these accessions (as well as the analyses performed in all individual libraries; data not shown), we conclude that the bootstrap confidence intervals from percentiles is not suitable for the estimation of CI in the case of the number of missing genes. As previously mentioned, the application of the methods to correct the bias of these intervals, as for example, the BC_a or ABC [5, 4] do not work in this case, given the lack of information in the sample about the frequencies of the missing genes; i.e., the sample (in general) do not have information about all the relevant parameters.

Given this, the only option let that we can see is to assume approximate normality for the estimators, in particular for h_6, and obtain an asymptotic and approximate CI around $\hat{h}_6$ employing the formula

(D-7) $$\hat{h}_6 \pm z_{\alpha/2} se(\hat{h}_6)$$

where $z_{\alpha/2}$ is the normal deviate at $\alpha/2$ Type I error. This is justified by the bell-shaped form of the histograms for $\hat{h}_6$ in the cases of complete samples (data not shown). However, given that the estimated of standard error based in the bootstrap, $se(\hat{h}_6)$, is likely to underestimate the true variability of the estimators, it is likely that the CI calculated by equation D-7, and presented in Supporting file 'S2 Excel' for all data analyzed will be underestimating the true length of the interval. In all datasets employed to design and validate the estimator h_6, the 95% CI calculated with equation D-7 included the value of zero, and thus also by this criterion, those can be considered as complete samples.

E. Calculating extra sample needed to estimate some of the missing genes

Having an estimate of the genes likely missing in a given sample, the researcher may want to increase the sample to have a better coverage, i.e., to estimate at least a percentage of the genes that were not already detected. This scheme is important because in many cases, having obtained a cDNA library from a transcriptome at high cost, extra sequencing runs can be performed at a fixed cost per run, decreasing the overall cost per read. In some cases it could be imperative to perform this extra sampling; for example, when the researcher need to be relatively sure that a gene or set of genes are not expressed at all in a given treatment.

Here we present and justify the use of a new estimator of the extra sample needed (m'_ψ, equation 9 in main text) that we demonstrate to be more precise and accurate than the one previously proposed in [2], m_ψ (equation 8 in main text).

E.1. **Comparing m_ψ with m'_ψ in subsamples of a complete dataset.** A problem to compare the estimators of the extra sample needed, m_ψ with m'_ψ, is that given that they use different estimators of f_0 (Chao1 and our harmonic estimator of degree 6, h_6, respectively), the number of genes wanted, say the proportion $\psi \hat{f}_0$ will be different (for the same particular sample) when employing the two alternative estimators. For example, in the study of real datasets (see Supporting file 'S2 Excel'), in 333 out of 342 cases (97%) we have a larger estimation of f_0 with h_6 than with Chao1. In Supporting file 'S2 Excel' for the Total in accession E-GEOD-16789 (id 3), the estimated values for the missing genes are 1,869 and 4,270 by Chao1 and h_6 respectively, thus setting the proportion of genes wanted at $\psi = 0.95$ we can calculate the extra sample needed to obtain $0.95 \times 1,869 = 1,776$ and $0.95 \times 4,270 = 4,057$ extra genes respectively. The corresponding values of extra sample size are $m_\psi = 1,567,695$ and $m'_\psi = 11,371,848$. As a result of the larger number of missing genes estimated with h_6 compared with Chao1 we have that in all real cases studied $m_\psi < m'_\psi$, and the average ratio of m'_ψ / m_ψ is equal to 17.5 for the real cases,

while the average ratio of the number of missing genes estimated by h_6 with reference to Chao1 is equal to 2.3 in the same cases. Given that the relation of sample size, N, and number of estimated genes, g, is not lineal, it is complex to compare which estimator, m_ψ or m'_ψ, is giving better results in the sense of having a better accuracy of prediction, or, equivalently a lower weighted squared error.

Denote as $\hat{f}_0|N$ and $g|N$ the estimated values of f_0 and G using a sample size N, respectively. Also we will write $m_\psi = m_\psi(N, Chao1)$; $m'_\psi = m_\psi(N, h_6)$ to make explicit the dependence of the extra sample function on the corresponding sample sizes and estimators. Then the errors of the estimators can be measured as the difference between the number of genes predicted by the estimation, say, $g + \psi\hat{f}_0|N$, and the expected number of genes in a new sample calculated with extra sample size, $E[G|N + m_\psi]$, thus the weighted square error of an estimator is given by

$$se(m_\psi) = \left(\frac{g + \psi\hat{f}_0|N - E[G|N + m_\psi]}{\hat{f}_0}\right)^2 \tag{E-8}$$

where we use g for the realized number of genes in the sample and G as the random variable of the number of observed genes in new samples, employing the larger sample size $N + m_\psi$. We divide the pure error (the numerator of equation 2) between the estimated value $\hat{f}_0$ to normalize the measure and make the use of distinct estimators comparable. The problem is, of course, to estimate $E[G|N + m_\psi]$ given that the distribution of G is unknown. However, having a complete sample, as the total of the mouse accession GSE1581, the value of $E[G|N + m_\psi]$ can be estimated, at least approximately, by the parametric bootstrap procedure for small samples. In this way we can substitute $E[G|N + m_\psi]$ by the realized value $\hat{g}|N + m_\psi$ where this value is obtained by the bootstrap procedure; for example, $\hat{g}|N + m_\psi$ could be the mean or median of B replicates obtained by parametric bootstrap. Here, to obtain fixed sample sizes, the multinomial instead of the Poisson distribution was employed.

More explicit forms of equation E-8 can be written for the weighted squared error of the estimation using Chao1 (m_ψ) and the h_6 estimator (m'_ψ), say

$$se(m_\psi) = se(m_\psi(N, Chao1)) \approx \left(\frac{g + \psi Chao1|N - \hat{g}|N + m_\psi(N, Chao1)}{Chao1}\right)^2$$

and

$$se(m'_\psi) = se(m'_\psi(N, h_6)) \approx \left(\frac{g + \psi\hat{h}_6|N - \hat{g}|N + m_\psi(N, \hat{h}_6)}{\hat{h}_6}\right)^2$$

where it is understood that the estimated value of $E[G|N + m_\psi]$, say $\hat{g}|N + m_\psi$, is obtained by the bootstrap procedure.

To obtain comparable values of $se(m_\psi)$ and $se(m'_\psi)$ we obtained $B = 160,000$ bootstrap pseudo replicates of the complete dataset ('total' of accession GSE1581) which has an original sample size of 160.55 million tags for a total of $G = 23,332$ genes. The bootstrap replicates were simulated varying the sample sizes from 0.5 to 10 million tags, and from these replicates we obtained 5,339 values for different statistics, including the estimated values of the standard errors of both methods to estimate the extra sample size. Table E-12 summarize these results.

In Table E-12 we can see the minimum, median, average, maximum and standard deviation (S), for the observed number of genes, g, the true value of the number of missing genes, f_0, the estimated values of f_0 by Chao1 and $\hat{h}_6$ as well as the extra sample calculated by both methods, $m_\psi(N, Chao1)$ and $m'_\psi(N, \hat{h}_6)$ as well as the estimated weighted squared errors for these estimators, $se(m_\psi(N, Chao1))$ and $se(m'_\psi(N, \hat{h}_6))$, respectively.

Form Table E-12 we can see that the observed number of genes in the samples varied between 15,777 and 21,886, i.e., between 68 and 94% of the true number of genes in the original sample ($G = 23,332$). We can also see that, as expected, the estimated number of missing genes with Chao1 was smaller -and farther away from the true value, than the corresponding value with the h_6 estimator; this is true for the

Table E-12. Statistics for the simulated values of extra sample size, m_ψ, and weighted squared errors, $se(m_\psi)$ obtained from $B = 160,000$ bootstrap replicates. N varied from 0.5 to 10 million tags and all estimated were obtained using $\psi = 0.95$

Statistic	Minimum	Median	Average	Maximum	S
g	15,777	20,206	19,805	21,886	1317
f_0	1,446	3,126	3,527	7,555	1,317
Chao1	651	1,398	1,476	2,673	396
$\hat{h}_6$	1,158	2,887	3,073	5,779	934
$m_\psi(N, Chao1)$	663	595,428	554,707	1,115,396	215,821
$m'_\psi(N, \hat{h}_6)$	72,112	4,106,925	3,881,850	5,529,171	873,663
$se(m_\psi(N, Chao1))$	0.05561	0.43831	0.45252	1.00807	0.23163
$se(m'_\psi(N, \hat{h}_6))$	0.06865	0.21799	0.22544	0.85843	0.08725

minimum, median, average and maximum, but the standard deviation, S, of the estimations is larger for h_6 than for Chao1. This is explained by the fact that Chao1 covers a smaller rank of estimated values than h_6.

On the other hand, the ratios of the extra sample sizes obtained with the h_6 and Chao methods, say $m'_\psi(N, \hat{h}_6)/m_\psi(N, Chao1)$ (rows 6 and 5 in Table E-12) are large; 108.8, 6.7, 7.0 and 5 for the minimum, median, average and maximum, respectively; i.e., the method with $\hat{h}_6$ always demands a larger extra sample size, in part because it will give more 'new' genes not detected with the original sample ($\hat{h}_6$ almost always is larger than Chao1) and also because the method employing h_6 is more accurate.

More important and conclusive, from rows 7 and 8 of Table E-12, we can see that the weighted square errors for the estimators using the Chao and h_6 estimators, $se(m_\psi(N, Chao1))$ and $se(m'_\psi(N, \hat{h}_6))$ respectively, are smaller for $se(m'_\psi(N, \hat{h}_6))$ compared with $se(m_\psi(N, Chao1))$ for the median, average, maximum and S, being larger only for the minimum. The ratios $se(m'_\psi(N, \hat{h}_6))/se(m_\psi(N, Chao1))$ are 1.23, 0.50, 0.50, 0.85 and 0.38 for the minimum, median. average, maximum and S, respectively. These simulations demonstrate that our estimator of the extra sample size have smaller errors over a large rank of sample sizes; judging by the central tendency (median and average of the square errors) we can conclude that the method employing h_6 to calculate the extra sample is around 50% more precise and reliable than the one proposed in [2] and also more robust, by having an estimated standard deviations of the weighted squared errors that represents around 38% of the one for $se(m_\psi(N, Chao1))$, $(0.08725/0.23163 \approx 0.38)$.

F. Comparing h_6 with iChao1 and other estimators

In August 28, 2014 we become aware of a recently published paper [3]. There Anne Chao and collaborators present a new estimator for the number of missing classes, '*iChao1*', that uses frequencies of frequencies f_3 and f_4 to improve the estimation of Chao1, which employs f_1 and f_2. Given that our estimator h_6 employs $f_1, f_2, \cdots, f_6$ it is important to compare the performance of iChao1 with h_6. First, a comparison using simulations with exactly the same models and parameters than in [3] and including the h_6 estimator was performed. From these comparisons we concluded that under that conditions[5] h_6 does not present significant advantages over iChao1 (results not shown, but a full report is available upon request).

Under the ecological framework h_6 does not present advantages over iChao1 because there the number of classes and sample sizes employed are not appropriate for the h_6 formulae. In many cases the 'high' frequencies f_5 or f_6, or both, are zero when the number of classes and sample sizes are small. This induces indeterminacy in the h_6 formula. However, we will see here that within the framework of RNA-seq, where

[5]Which are tailored to mimmic the conditions of estimation of the missing number of *species* in Ecology, and not missing genes in RNA-seq as h_6.

the number of classes is in the order of tens of thousands and the sample sizes are in the order of, at least, hundreds of thousands, h_6 is superior to iChao1 by having smallest bias.

The estimators compared in [3], using our notation, are

- Chao1: $\hat{G} = g + (f_1^2/2f_2)$
- iChao1 (improved Chao1): $\hat{G} = g + (f_1^2/2f_2) + (f_3/4f_4)\max(f_1 - (f_2f_3/2f_4), 0)$ (Note that this is equal to $\hat{G} = Chao1 + (f_3/4f_4)\max(f_1 - (f_2f_3/2f_4), 0)$)
- First order Jackknife (JK1): $\hat{G} = g + f_1$
- Second order Jackknife (JK2): $\hat{G} = g + 2f_1 - f_2$
- Lanumteang-Böhning (LB) [8]: $\hat{G} = g + 3f_1^3 f3/4f_2^3$

In their publications Chao and col. focus on the estimation of the total number of classes, G in our notation, which is equal to

$$\hat{G} = g + \hat{f}_0$$

where g is the observed number of classes and $\hat{f}_0$ is some estimator of the number of missing classes.

To avoid indeterminacy in the estimation of h_6 we modify the the definition of the harmonic mean to be

$$H^*(f_2, f_3, \cdots, f_6) = \frac{\sum_{i=2}^{i=6} I_{\neq 0}(f_i)}{\sum_{f_i \neq 0} 1/f_i}$$

i.e., we calculate the harmonic mean using only the values of $f_i; i = 2, 3, \cdots, 6$ which are larger than zero, and let $h_6^* = (6/10)(f_1^2)/H*(f_2, f_3, \cdots, f_6)$. We have then two putative estimators of G, say

$$\hat{G}(h_6) = g + h_6;\ \hat{G}(h_6^*) = g + h_6^*$$

Note, for example, that the estimator $\hat{G}(h_6^*)$ takes a value of $g + (6/10)f_1^2 f_2$ if $f_2 > 0$ and simultaneously $f_3 = f_4 = f_5 = f_6 = 0$. This will give a very high and surly inaccurate estimation, thus, as a rule of thumb, our estimator h_6 can be employed only in the cases where all values $f_i; i = 2, 3, \cdots, 6$ are larger than zero.

To test the estimators in the framework of RNA-seq we used the GSE1581 dataset, corresponding to the 'MPSS mouse transcriptome analysis project' . The GSE1581 dataset is complete by the criterion of $f_1 = 0$ and contains expression information for a total of 23,332 genes. To compare the performance of the G estimators we performed simulations using the multinomial distribution with probabilities equal to the relative frequencies of the genes in the GSE1581 dataset and employing sample sizes $N = 1 \times 10^3, 0.5 \times 10^4, 1 \times 10^4, 0.5 \times 10^5, 1 \times 10^5, 0.5 \times 10^6, 1 \times 10^6, 0.5 \times 10^7$, i.e., from 1,000 to 5,000,000 gene tags.

Figures F-20 and F-21 present the means and 95% percentile intervals for the estimations of $\hat{G}$ employing 6 estimators of G.

From Figure F-20 we can see that for all sample sizes larger or equal to 50,000 ($\log_{10}(0.5e5) \approx 4.7$ in the plot) all estimators underestimate the true value of G, while for values of $N < 50,000$ the estimator based in h_6, $\hat{G}(h_6^*)$, overestimate G while all the other estimators underestimate the true value $G = 23,332$, marked by a grey horizontal line in the plot. For $N \geq 50,000$ the $\hat{G}(h_6^*)$ estimator has, consistently, the smallest bias.

In Figure F-21 (a close-up of Figure F-20) we can examine the behavior of the estimators for sample sizes $1 \times 10^6 \leq N \leq 0.5 \times 10^7$. At $N = 1 \times 10^6$ none of the 95% percentile intervals include the true value of $G = 23,332$, while for $N = 0.5 \times 10^7$ (five million gene tags) the only 95% percentile interval that includes $G = 23,332$ is the one corresponding to $\hat{G}(h_6^*)$. At this point ($N = 0.5 \times 10^7$; five million gene tags) the mean of the 1000 simulations closest to the true value is the one corresponding to $\hat{G}(h_6^*)$, followed by the ones given by the JK2, LB, JK1, iChao1 and Chao1 estimators, respectively.

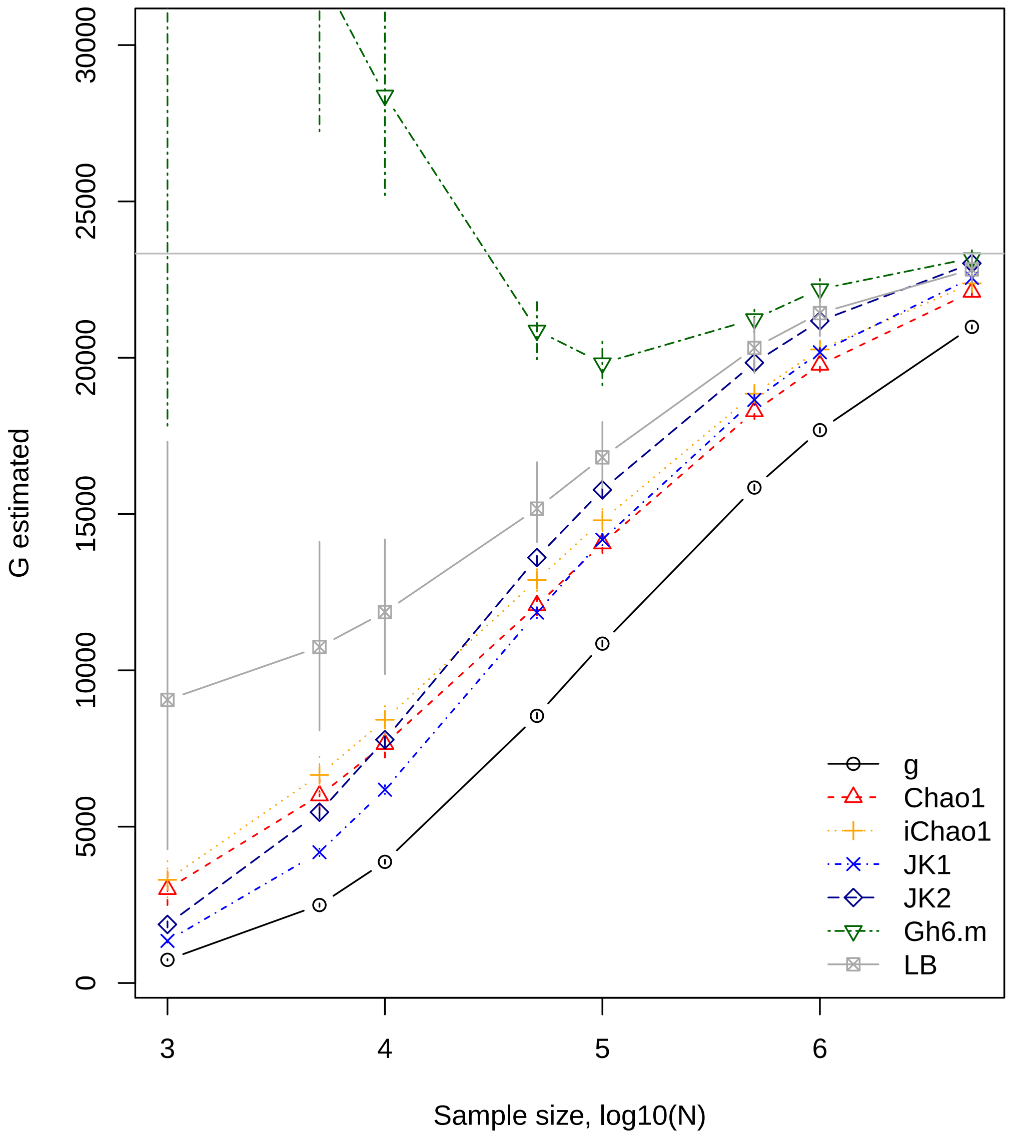

FIGURE F-20. Scatter plot for the means of estimations of $\hat{G}$ in 1000 samples of the complete RNA-seq dataset 'GSE1581' with different sample sizes given in $\log_{10}(N)$ in the X axis and corresponding to $N = 1000, 5000, 10000, 50000, 100000, 500000, 1000000, 5000000$ gene tags. Vertical lines are the 95% percentile of the distributions of the corresponding means. 'Gh6.m' denote the estimator G using h_6, that is, $\hat{G}(h_6^*)$, while other estimators are referred by name.

To make a direct comparison between the estimators Chao1, iChao1 and h_6 of f_0 we run 12,500 new simulations (datasets) with sample sizes, N, uniformly distributed between 45,000 and 5 million tags. Figure F-22 presents the scatter plot of f_0 (true value, X-axis) *versus* the estimated values, $\hat{f}$ (Y-axis), employing the estimators h_6, Chao1 and iChao1.

In Figure F-22 we can see how Chao1 and iChao1 estimators generally and consistently underestimate the true value of f_0 in the simulations performed varying the sample size. This underestimation depends on the true value of f_0 and is always larger for Chao1 compared with iChao1. In contrast, when the true value of f_0 is smaller than 4,000 the estimations of f_0 by h_6 are clos to the true value, and for cases where

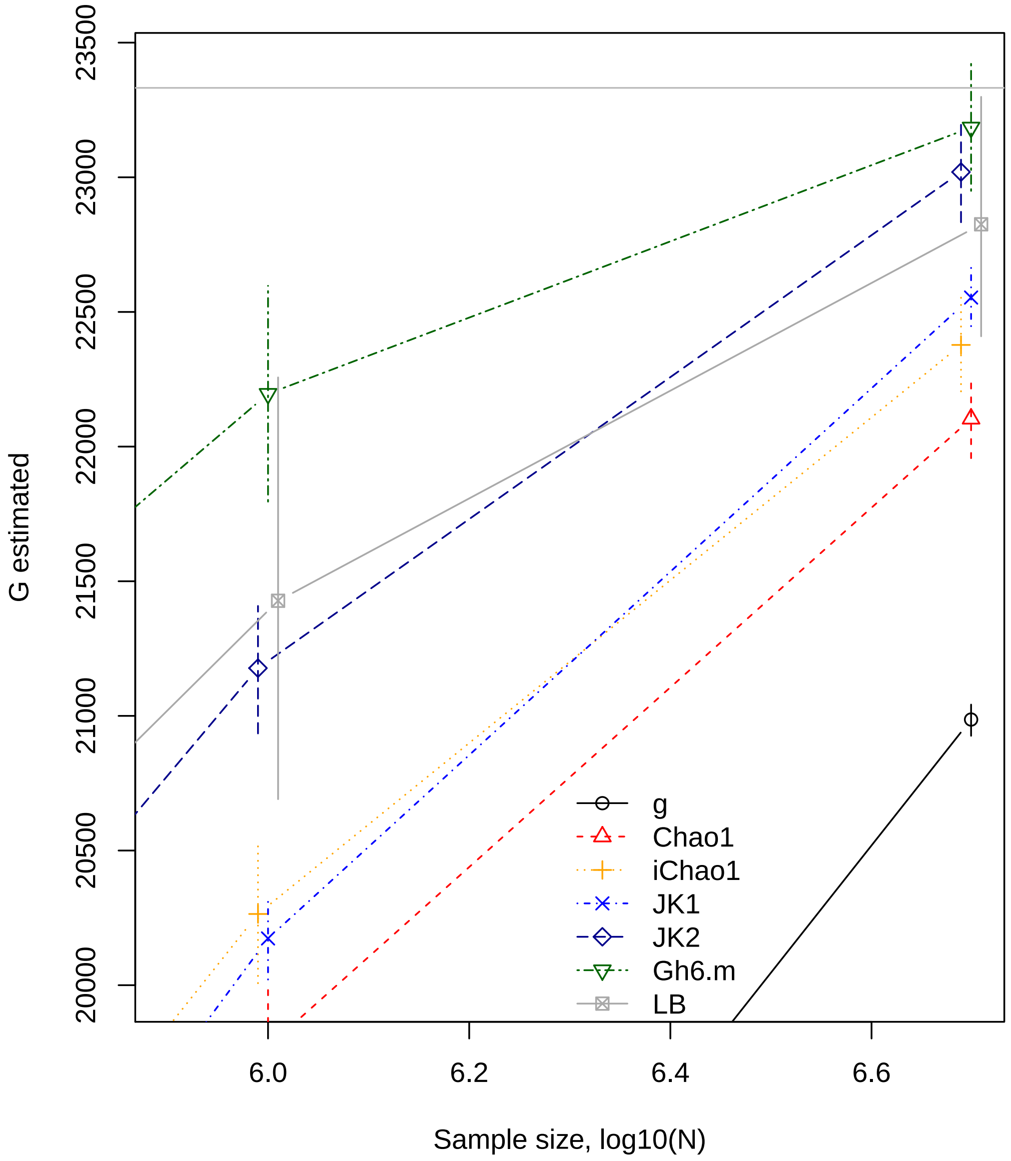

FIGURE F-21. Close-up of Figure B-1 (see that caption, here we present sample sizes N equal to 1000000, 5000000 gene tags; $\log_{10}(1000000) = 6.0, \log_{10}(5000000) = 6.7$). Values of the sample sizes of each estimator were varied $\pm 0.01 \log_{10}(N)$ to avoid overlapping in the percentile intervals.

$f_0 > 4000$ the underestimation of f_0 by h_6 are always smaller than for Chao1 and iChao1. This is more notorious for values of $f_0 > 12000$.

Figure F-23 shows the realized bias $(\hat{f}_0 - f_0)$ for the three estimators in the same simulations than the ones presented in Figure B-3. In this case the bias is presented as function of the sample size, N.

From Figure F-23 we can see that even for small samples of less than one million gene tags ($N < 1e6$) the bias of the estimations by h_6 are smaller than for Chao's estimators (Chao1 and iChao1). For samples of around 2 million tags ($N \geq 2e6$) the distribution of the estimates by h_6 include the null value of bias $(\hat{f}_0 - f_0 = 0)$, while that is not the case for Chao1 or iChao1.

From the analyses presented we conclude that h_6, is superior to the one based in the iChao1 estimator for the framework of transcriptomes sampled by RNA-seq. This is due to the large number of classes

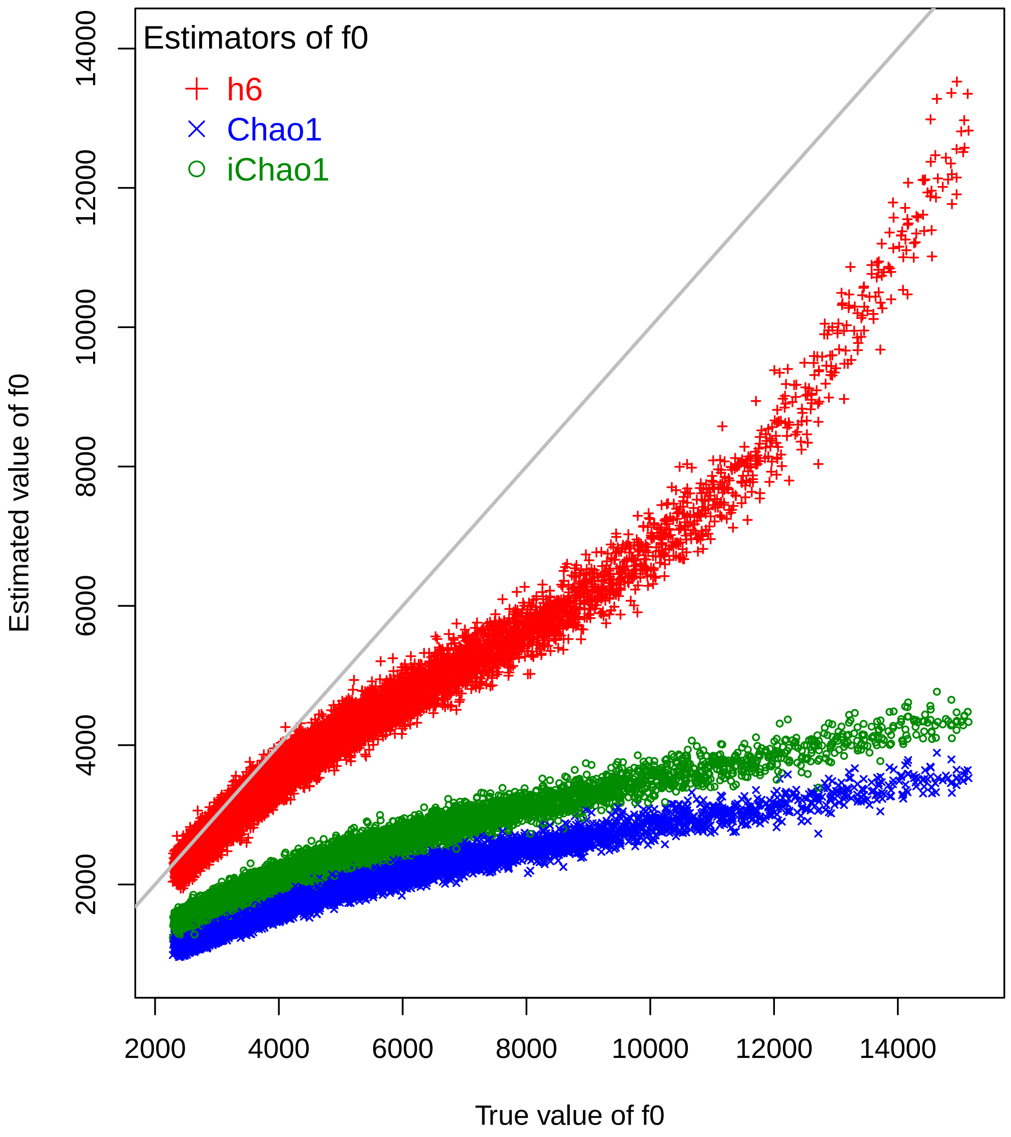

FIGURE F-22. Scatter plot for the true value of the number of missing genes, f_0 X-axis, *versus* the corresponding estimate, $\hat{f}_0$ Y-axis, estimated in each case by the three estimators, h_6, Chao1 and iChao1 in 12,500 samples of random size N uniformly distributed between 45,000 and 5 million tags. Grey line signal points $f_0 = \hat{f}_0$.

(genes), large sample sizes and particular probability structure in the transcriptomes, which is in contrast with the cases of samples in Ecology for which Chao's estimators were designed.

G. R functions

G.1. **sample.Pf0.** This function use the estimated frequencies of the genes in an RNA-seq library to estimate the probability of having $P[f_0 > 0] = \alpha$ by minimizing the square error given in equation B-3. To avoid divergence in very large samples, the search is restricted to the interval $(N/2, 20N)$.

```
sample.Pf0 <- function(x, alpha=0.05){
# sample.Pf0
# Calculates the sample size, T, needed to obtain
```

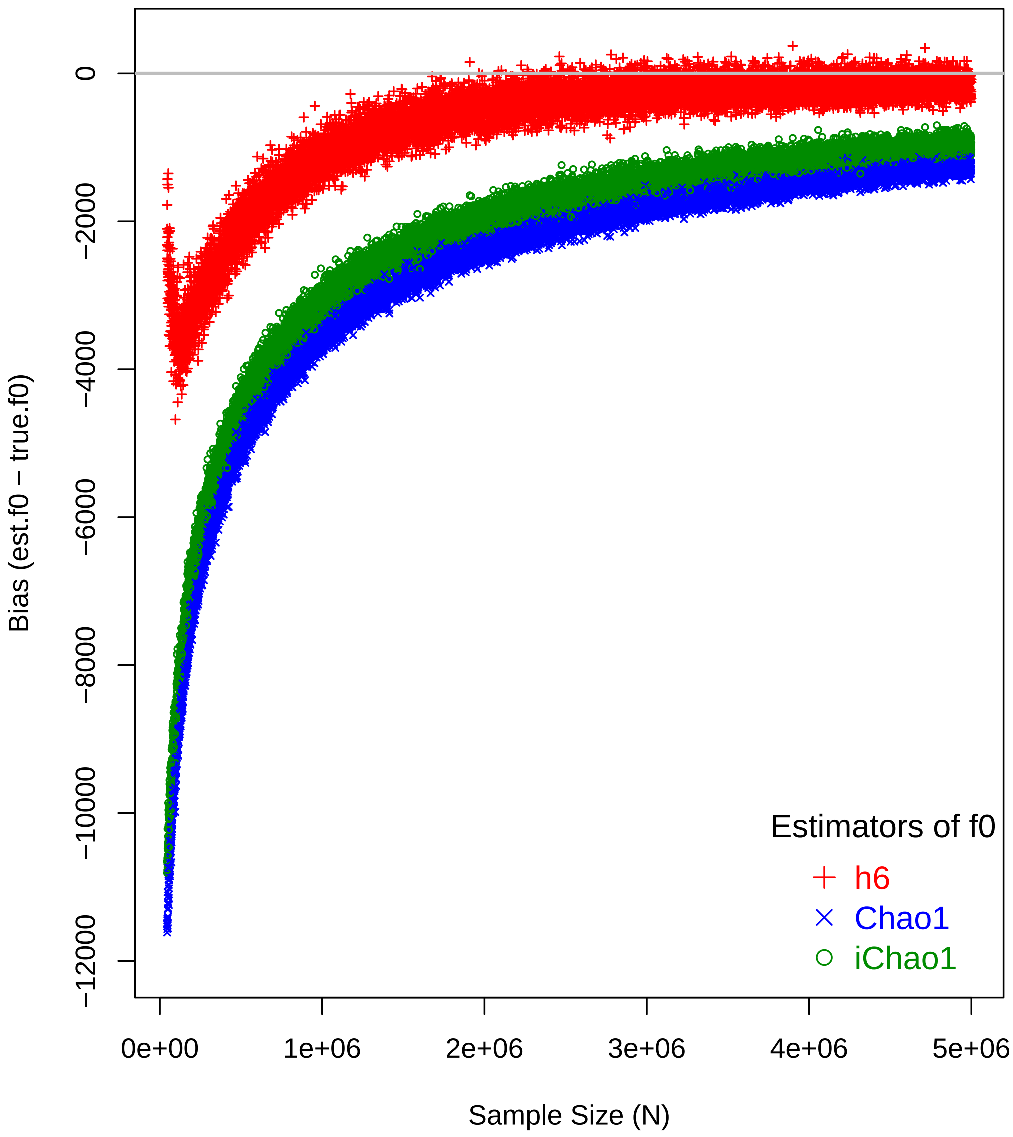

FIGURE F-23. Scatter plot for the bias ($\hat{f}_0 - f_0$) of the estimates of f_0 (Y-axis) by h_6, Chao1 and iChao1, in relation with sample size, N (X-axis), in 12,500 datasets. Sample size, N, was uniformly distributed between 45,000 and 5 million tags (same data than in Figure B-3).

```
# a probability alpha to have f0>0 (a "complete" sample)
# x - Vector of gene counts from an RNA-seq experiment.
# See: BitacoraR_462.txt
x <- x[x>0] # Takes out zeroes
# Function to be minimized:
to.opt <- function(T, prob, alpha){(1 - prod(1 - exp(-T*prob))-alpha)^2}
N <- sum(x) # Original sample size
p <- x/N # Estimated probabilities
the.min <- optimize(f = to.opt, interval = c(N/2, 20*N), prob=p, alpha=alpha, tol=1e-6)
res <- c(N, the.min$minimum, the.min$minimum/N, alpha, the.min$objective)
names(res) <- c("N", "T", "rTN", "alpha", "error")
res
```

```
}
```

G.2. **The R package '`UndetectedGenes`'.** This package codes for the estimation of f_0 *via* the h_6 estimator and also contains auxiliary functions.

The file '`UndetectedGenes_0.90.tar.gz`' contains the R package. This can be downloaded from our lab at

`http://computational.biology.langebio.cinvestav.mx/DOWNLOAD/UndetectedGenes/`

To install this package you need to type (at the system prompt and within the directory that contains the file):

```
R CMD install UndetectedGenes
```

(if you have a problem with that, please refer to your particular version of the R help for package installation)

Having installed the package and in an R command window you can make the package available by typing '`library(UndetectedGenes)`' at the R prompt.

'`UndetectedGenes`' contains a single function, '`h6`', which only compulsory input is the vector of counts in an RNA-seq experiments. See the help of this function for details ('`? h6`' at the R prompt).

References

[1] Anne Chao. Nonparametric estimation of the number of classes in a population. *Scandinavian Journal of statistics*, pages 265–270, 1984.

[2] Anne Chao, Robert K Colwell, Chih-Wei Lin, and Nicholas J Gotelli. Sufficient sampling for asymptotic minimum species richness estimators. *Ecology*, 90(4):1125–1133, 2009.

[3] Chun-Huo Chiu, Yi-Ting Wang, Bruno A Walther, and Anne Chao. An improved nonparametric lower bound of species richness via a modified good–turing frequency formula. *Biometrics*, 70(3):671–682, 2014.

[4] Anthony Christopher Davison. *Bootstrap methods and their application*, volume 1. Cambridge university press, 1997.

[5] Bradley Efron and Robert J Tibshirani. *An introduction to the bootstrap.* Chapman & Hall, New York - London, 1st. edition, 1993.

[6] I. J. Good. The Population Frequencies of Species and the Estimation of Population Parameters. *Biometrika*, 40(3):237–264, 1953.

[7] C Victor Jongeneel, Mauro Delorenzi, Christian Iseli, Daixing Zhou, Christian D Haudenschild, Irina Khrebtukova, Dmitry Kuznetsov, Brian J Stevenson, Robert L Strausberg, Andrew J G Simpson, and Thomas J Vasicek. An atlas of human gene expression from massively parallel signature sequencing (MPSS). *Genome research*, 15(7):1007–14, July 2005.

[8] Krisana Lanumteang and Dankmar Böhning. An extension of chao's estimator of population size based on the first three capture frequency counts. *Computational Statistics & Data Analysis*, 55(7):2302–2311, 2011.

[9] Octavio Martínez and M Humberto Reyes-Valdés. Defining diversity, specialization, and gene specificity in transcriptomes through information theory. *Proceedings of the National Academy of Sciences*, 105(28):9709–9714, 2008.

[10] Luis A Martínez-López, Neftalí Ochoa-Alejo, and Octavio Martínez. Dynamics of the chili pepper transcriptome during fruit development. *BMC genomics*, 15(1):143, 2014.

[11] R Core Team. *R: A Language and Environment for Statistical Computing.* R Foundation for Statistical Computing, Vienna, Austria, 2013. ISBN 3-900051-07-0.

[12] Changjiang Xu, Luzhou Xu, Fahong Yu, Weihong Tan, Leonid L Moroz, and Jian Li. Nonparametric estimation of the number of unique sequences in biological samples. *Signal Processing, IEEE Transactions on*, 54(10):3759–3767, 2006.
